# Theoretical Insights into the Biophysics of Protein Bi-stability and Evolutionary Switches

**DOI:** 10.1371/journal.pcbi.1004960

**Published:** 2016-06-02

**Authors:** Tobias Sikosek, Heinrich Krobath, Hue Sun Chan

**Affiliations:** Departments of Biochemistry and Molecular Genetics, University of Toronto, Toronto, Ontario, Canada; Iowa State University, UNITED STATES

## Abstract

Deciphering the effects of nonsynonymous mutations on protein structure is central to many areas of biomedical research and is of fundamental importance to the study of molecular evolution. Much of the investigation of protein evolution has focused on mutations that leave a protein’s folded structure essentially unchanged. However, to evolve novel folds of proteins, mutations that lead to large conformational modifications have to be involved. Unraveling the basic biophysics of such mutations is a challenge to theory, especially when only one or two amino acid substitutions cause a large-scale conformational switch. Among the few such mutational switches identified experimentally, the one between the G_A_ all-α and G_B_ α+β folds is extensively characterized; but all-atom simulations using fully transferrable potentials have not been able to account for this striking switching behavior. Here we introduce an explicit-chain model that combines structure-based native biases for multiple alternative structures with a general physical atomic force field, and apply this construct to twelve mutants spanning the sequence variation between G_A_ and G_B_. In agreement with experiment, we observe conformational switching from G_A_ to G_B_ upon a single L45Y substitution in the GA98 mutant. In line with the latent evolutionary potential concept, our model shows a gradual sequence-dependent change in fold preference in the mutants before this switch. Our analysis also indicates that a sharp G_A_/G_B_ switch may arise from the orientation dependence of aromatic π-interactions. These findings provide physical insights toward rationalizing, predicting and designing evolutionary conformational switches.

## Author Summary

The biological functions of globular proteins are intimately related to their folded structures and their associated conformational fluctuations. Evolution of new structures is an important avenue to new functions. Although many mutations do not change the folded state, experiments indicate that a single amino acid substitution can lead to a drastic change in the folded structure. The physics of this switch-like behavior remains to be elucidated. Here we develop a computational model for the relevant physical forces, showing that mutations can lead to new folds by passing through intermediate sequences where the old and new folds occur with varying probabilities. Our approach helps provide a general physical account of conformational switching in evolution and mutational effects on conformational dynamics.

## Introduction

The role of protein biophysics is increasingly recognized in the study of evolution, and the study of protein biophysics has also benefitted from evolutionary information [1–4]. Emerging from a more physical perspective of molecular evolution is the realization that natural selection can act on a nonsynonymous mutation as long as it modifies the conformational distribution, even if it leaves the folded structure of a protein unchanged and maintains the original biological function. For instance, if the mutation stabilizes a nonnative “excited” conformational state which is structurally distinct from native, this state can potentially serve an additional “promiscuous” biological function which is then subject to natural selection [5]. This effect, demonstrated experimentally [6], is a direct consequence of the ensemble nature of protein conformations and follows simply from the principle of Boltzmann distribution [7,8]. Similarly, even if the most stable structure of a protein is robust against a mutation, the protein’s functional structural dynamics can be modulated by the mutation, which should then also be subjected to natural selection [5,9]. In this way, positive selection of an excited conformational state favors mutations that gradually increase the stability of the excited state, so that it finally becomes the new dominant native structure or one of two (or more) native structures with comparable stabilities in a “bi-stable” (or “multi-stable”) protein. Protein sequences interconnected by mutations and encoding for the same folded structure form neutral networks [10]. Bi-stability was predicted to occur at the intersection of neutral networks [8,10].

Consistent with theory [7,8,11–14], some phylogenetically reconstructed ancestral proteins are bi-stable [15]. Although there is no direct measurement to date of a gradually shifting conformational equilibrium for a set of naturally occurring amino acid sequences traversing two neutral networks, recent advances in NMR spectroscopy allow mutational changes in the stability of nonnative excited states to be detected [16]. A handful of conformational switches and bi-stable sequences have now been designed in the laboratory [17–19]. Among them, the one that is most extensively characterized is the set of designed mutant sequences that span the human serum albumin-binding and IgG-binding domains of *Streptococcus* protein G [19,20]. The wildtype sequences of these proteins, termed GAwt and GBwt respectively, are of equal length (56 residues) in the experimental system. GAwt and GBwt have only 16% sequence identity with very different folded structures. GAwt folds to a three-helix bundle (3α), whereas folded GBwt is a helix packing against a four-stranded β-sheet (4β+α). By carefully selecting amino acid substitutions, Alexander et al. created mutant sequence pairs with 30%, 77%, 88%, 95%, and 98% identity while still maintaining the original different folds. A single L45Y substitution separates the pair of mutants GA98 and GB98 with 98% identity. L45Y switches the dominant fold of GA98 from that of G_A_ (3α) to that of G_B_ (4β+α) for GB98 [19,20]. As suggested by theory [7,8] and by molecular dynamics simulations of the unfolded states of the GA88/GB88 pair [21], appreciable excited-state populations for the alternative fold should be present in the GA/GB mutants with 95%, 88%, or even 77% identity. Ligand binding data provide evidence that GA98 and another mutant GB98-T25I that also adopts the 3α G_A_ fold have excited-state populations of the alternative G_B_ fold. However, GB98-T25I is the only mutant for which the alternative fold is directly observable by NMR [22], as nonnative populations lower than ~1% are currently difficult to detect experimentally. By simulating the folding energy landscapes of the mutants, the goal of the present computational analysis is to gain physical insights into the mechanism of the G_A_/G_B_ conformational switch, including how it might evolve via a gradual increase in stability of the alternate fold as the mutants approach the switch.

The most direct method of molecular simulation is to use a completely general physics-based potential. Such an approach has succeeded recently in showing that it is computationally possible for a series of mutants of a 16-residue peptide to undergo an α to β switch [14]. Owing perhaps to the limitations of molecular dynamics forcefields [23,24], folding simulations with fully transferrable potentials have not reproduced much of the switching behavior of the larger G_A_/G_B_ system [25,26], although complementary theoretical methods have made useful progress. For instance, some of the GA/GB mutants can be assigned to their correct native folds by various threading approaches [8,27] or a “confine-and-release” simulation algorithm applied to the GA88/GB88 and GA95/GB95 pairs [28], suggesting that the forcefields used in these techniques may be quite adequate. But the conformations sampled by these techniques are limited only to those very similar to the G_A_ and G_B_ folded structures [8,27], or at best include also a highly confined set of conformations between them [28]. As such, the rather restricted conformational sampling in these techniques can mask possible shortcomings of the forcefields, e.g., by missing low-energy conformations that the techniques fail to sample. Therefore, to address fundamental physics of the G_A_/G_B_ system, as for any protein folding study, it is necessary to employ self-contained explicit-chain models that extensively sample both the folded and unfolded conformations [29].

One class of self-contained models proven useful in biomolecular studies is the Gō-like explicit-chain structure-based models (SBMs). These models are native-centric in that the only contacts favored by the potential are those that exist in the known native structures [29–32]. Most SBMs studied to date are single-basin in that they target a single native structure; but dual- and multi-basin SBMs can be constructed to fold to two or more native structures. The latter approach has been employed to analyze the conformational switches between different functional states of a protein [33–36]. A prime example is the large-scale allosteric conformational transition between the open and close forms of adenylate kinase [34,37]. Recent applications of dual-basin all-atom SBMs to the G_A_/G_B_ system suggest that the conformational preferences of some of the mutants can be rationalized to an extent by their differences in steric packing [38,39]. However, the effects of nonnative interactions that are not present in either the G_A_ or G_B_ folds are not considered in these SBMs; but nonnative interactions are important for protein evolution because they may lead to detrimental aggregation [40–42]. In any event, the extent to which these dual-basin SBMs are generalizable is not clear. They have only been applied to a small number of mutants, viz., GA95/GB95 and GA98/GB98 in ref. [38] and GA98/GB98 in ref. [39]. Moreover, in some cases, it appears necessary to single out contacts involving the mutated residues for *ad hoc* treatment [38].

To delineate the utility and limitation of common physical notions in accounting for experimental G_A_/G_B_ observations, we introduce a model that combines a SBM potential with a physics-based transferrable all-atom potential. Going beyond prior efforts that considered only two or four sequences, our model is applied coherently to an extensive set of twelve GA/GB sequence variants covering the 3α and 4β+α folds. Favorable nonnative contacts are possible in our formulation because of the transferrable terms. This “hybrid” modeling approach recognizes that current knowledge of protein energetics is not sufficiently adequate—thus the need for a native-centric bias—yet at the same time posits that physical nonnative effects should manifest at least as a perturbation [43]. Within this conceptual framework, the transferrable component represents what we believe we know physically, whereas the SBM component represents the extent of our ignorance, which we should aim to eliminate in the future. To tackle the G_A_/G_B_ system, we generalize the well-studied hybrid approach for a single-basin SBM [43–50] to one based upon a dual-basin SBM [33–36,51]. The formalism is general, however, and thus should be applicable also to conformational switches other than G_A_/G_B_.

As detailed below, the G_A_/G_B_ switching predicted by our model agrees with experiment. Moreover, the robustness and physicality of our predictions are buttressed by control simulations indicating a lack of folding of decoy protein sequences with folded structures very different from that of either G_A_ or G_B_. Interestingly, refinements of the transferrable component in our potential to better account for the π-interactions of aromatic residues [52] leads to a sharper conformational switch, suggesting that incorporation of more accurate descriptions of the physical interactions can lead to tangible improvement of the model under the present framework.

## Results

### A working hybrid model for G_A_/G_B_ physics with minimal native-centricity

**Fig 1:**
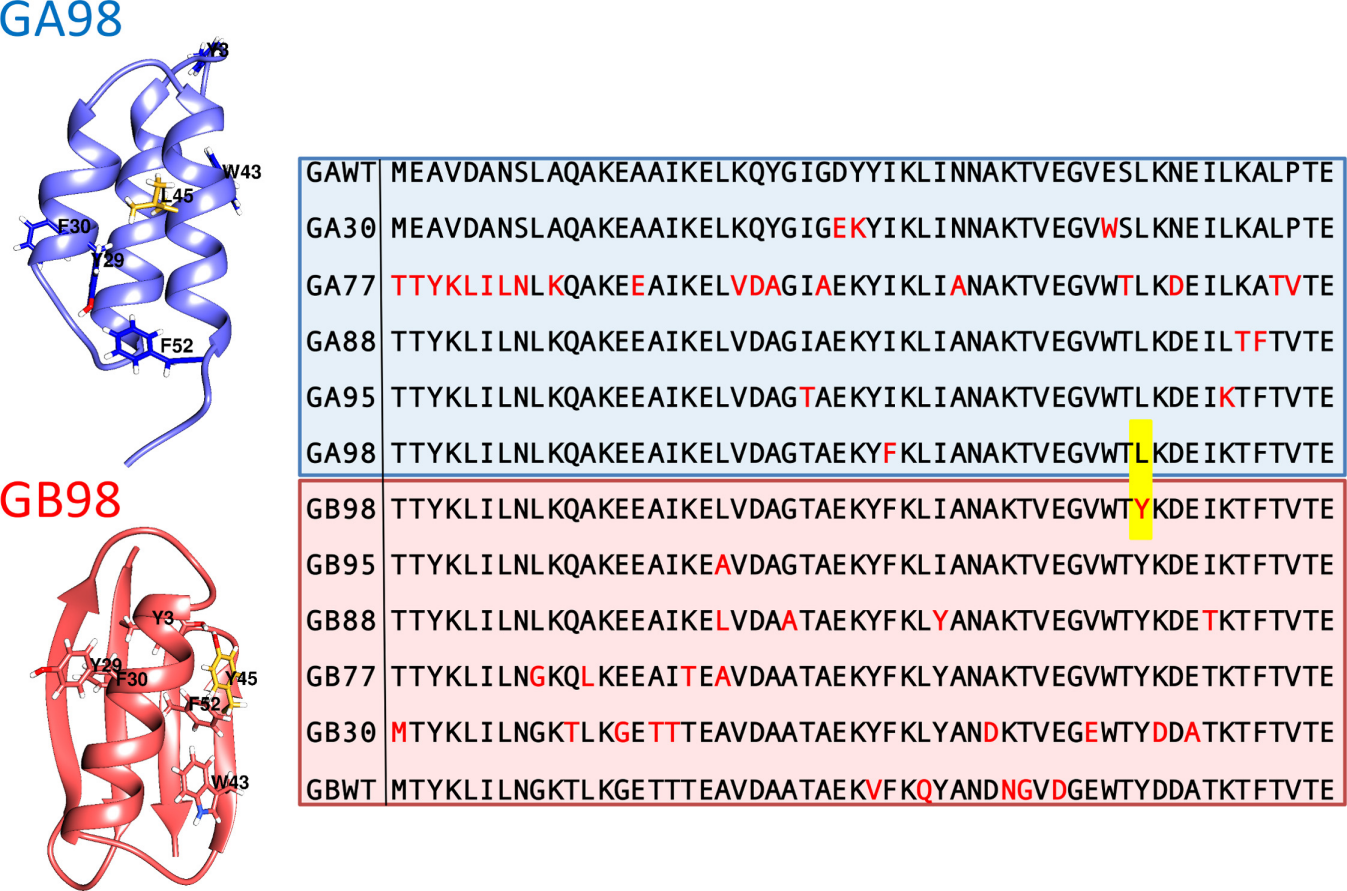
The twelve GA/GB sequence variants used in our computational investigation. Sequences range from wildtype GAwt to GBwt. Intermediate sequences are labeled by their pairwise sequence similarity, e.g. GA88 and GB88 are 88% identical [19]. From top to bottom, new amino acid substitutions are marked in red. The L45Y mutation is highlighted with yellow shading. The blue (G_A_) and red (G_B_) backgrounds indicate the experimentally observed native fold of the sequences. As an example, the ribbon diagrams show the experimental folded structures of GA98 and GB98 with residue 45 in yellow and aromatic residues depicted as sticks.

As noted above, SBMs are valuable conceptual tools; but SBMs and hybrid models are admittedly interim measures. Ultimately, one wishes to simulate biomolecular processes using a completely transferrable physical potential. With this in mind, to maximize the physical content, our hybrid model was constructed with a native-centric, structure-based component as nonspecific and as unimposing as we found technically possible. For example, in contrast to previous all-atom SBMs for G_A_/G_B_ [38,39] that enforce detailed native biases on dihedral angles and inter-atom distances [32], the SBM component of our hybrid model constrains only the C_α_-C_α_ distances between residues that are at least three sequence positions apart. The rest of the interactions—including local backbone preferences and sidechain excluded volume—are provided entirely by the transferable component. The SBM component of our model is sequence independent, in that the same native-centric potential applies to all GA/GB variants (**Fig 1**). In this way, the spatially coarse-grained SBM component serves merely to enable folding to the G_A_ or G_B_ native structures in an unbiased manner, all the while reducing as much as possible any artefactual memory of the sequence-structure relationship of any particular sequence. Accordingly, the differences in population in the two alternate folds for different sequences are determined solely by the physical transferable potential that admits nonnative as well as native interactions.

**Fig 2:**
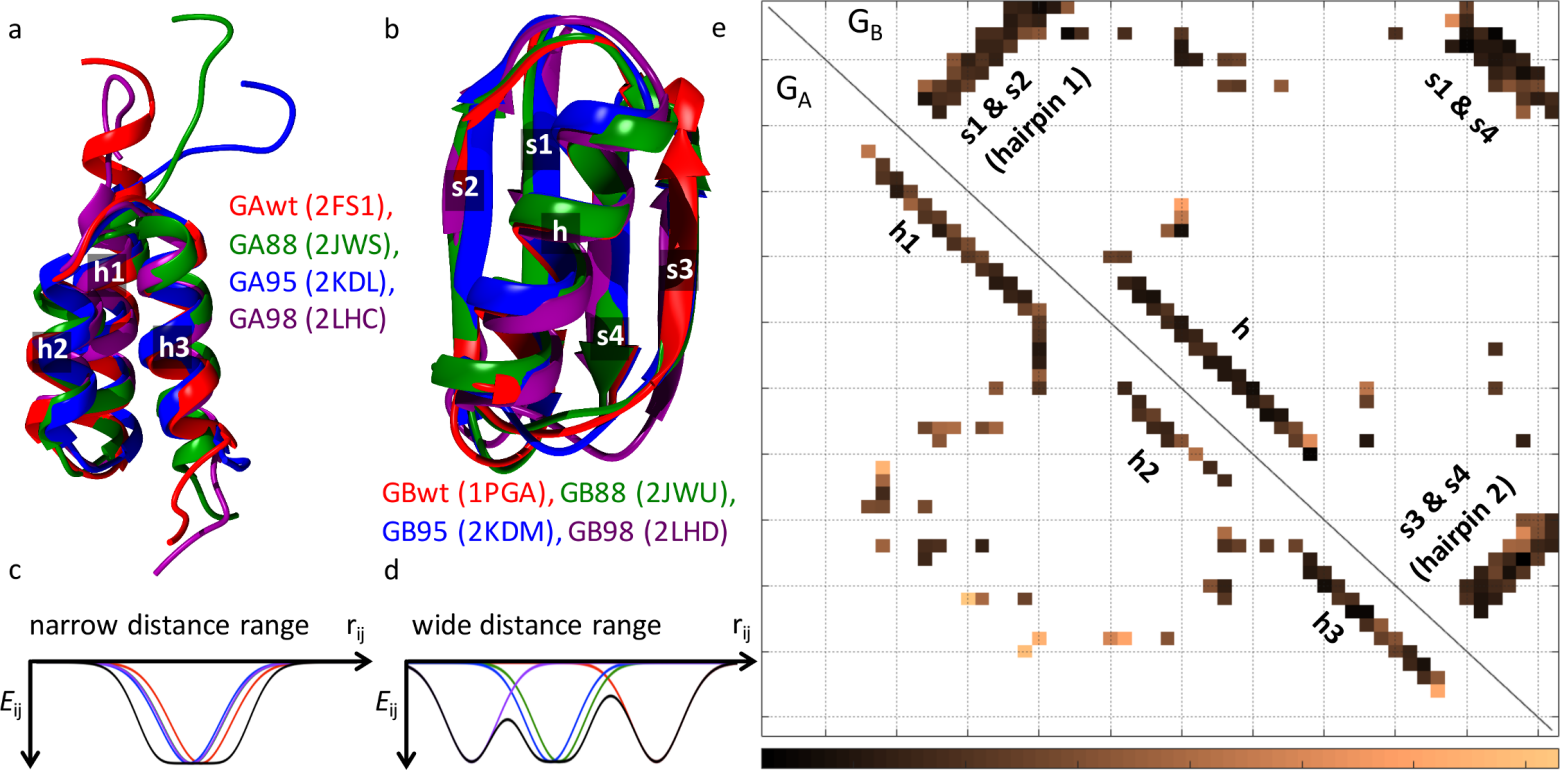
Consensus native contact maps and multi-Gaussian contact potentials for the SBM component of the hybrid model. (a,b) Four PDB structures each (IDs in parentheses) of GA (a) and GB (b) variants used to construct the contact potentials (c,d) and define the 95 G_A_ contacts and 137 G_B_ contacts in their respective consensus native contact maps (e). The numbers in (a,b) are labels for secondary structure elements (h for helix; s for sheet). (c,d) Examples of multi-Gaussian contact potentials with a given well depth ε (= ε_A_ or ε_B_). *E*_*ij*_ is the SBM contact energy between residues *i* and *j* as a function of their Cα-Cα distance *r*_*ij*_. Black curves are the consensus potentials whereas the color curves show the energy term for the individual contacts in one of the four contributing PDB structures (*Methods*). The examples here are for (c) G_A_ residue pair (42, 47) and (d) G_A_ residue pair (15, 46). (e) Consensus native contact maps for G_A_ (lower triangle) and G_B_ (upper triangle) with positions of secondary structure elements marked. The range of Cα-Cα distance *d*^(*s*)^_*ij*_ (by varying the native conformer label *s*) for every consensus native contacts *i*,*j* (color squares) is indicated by degree of shading (bottom scale). No consensus native contact is registered near the G_A_ termini because these regions are disordered in some of the PDB structures for G_A_.

As described in *Methods*, the present sequence-independent SBM component is based on the consensus C_α_-C_α_ native contact maps for G_A_ and G_B_. Each consensus map was constructed using the four PDB structures for GA or GB variants for which experimental folded structures are available (**Fig 2a and 2b**). The consensus map contains only the native contacts common to all four PDB structures. Two residues of a given PDB structure are defined to form a native contact if the closest distance between any two non-hydrogen sidechain atoms, one from each residue, does not exceed 6 Å. Here the SBM energy for each consensus residue-residue native contact is constructed as a multi-Gaussian well potential [53], wherein the position of the minimum for each of the wells is determined by the four defining PDB structures. In most cases, the individual minima fuse into a single wider well because they are in close proximity (**Fig 2c**), although in some cases they retain their distinct minima when there are larger variations in contact distances among the PDB structures (**Fig 2d**). The potentials for all contacts in the two consensus native contact maps (**Fig 2e**) are provided in **S1 Fig and S2 Fig**.

Summing the energy terms for individual consensus native G_A_ contacts gives the overall native-centric potential *E*_A_ for G_A_ and *E*_B_ for G_B_, the strengths of which are given, respectively, by ε_A_ and ε_B_ (*Methods*). A bi-stable SBM potential, *E*_SBM_, is then obtained by combining *E*_A_ and *E*_B_. The multi-Gaussian contact potentials here ensure that all native conformers used as input for the SBM potential are at an energy minimum of the same depth (ε_A_ or ε_B_) for a given fold. This approach captures the salient features of the two folds while allowing sufficient flexibility to accommodate variations in backbone and sidechain configurations among different GA/GB sequences.

To achieve an unbiased baseline sampling of the G_A_ and G_B_ folds, the SBM energy scales ε_A_ and ε_B_ are expected to be somewhat different and thus a calibration is necessary. Indeed, it has long been known from the study of single-basin SBMs that imposing a single SBM energy scale for different native structures would result in a spurious correlation between folding temperature and native contact density that is not observed experimentally [54]. For our system, the G_A_ fold was found to be only slightly more dominant in test simulations using min(*E*_A_) = min(*E*_B_) and the G_B_ fold was only slightly more dominant for ε_A_ = ε_B_. (Supporting Information **S1 Text** and **S3a and S3b Fig** and **S3c and S3d Fig**, respectively), whereas ε_A_ = 0.96ε_B_ allows for unbiased baseline sampling to produce results consistent with experiment. To minimize native-centricity as much as possible, we have examined the effect of different overall SBM interaction strengths and arrived at a workable value of ε_B_ = −0.37 (**S1 Text**, **S4 Fig** and **S5 Fig**). This strength corresponds to a weak native bias relative to the transferrable component, yet strong enough to guide folding. Under ε_B_ = −0.37, on average only less than one third (18.9/60.2 = 0.31) of the stabilization of GB98 is contributed by the SBM component *E*_SBM_ (**S6 Fig**). The rest (69%) is contributed by the transferrable *E*_trans_. Further analyses in **S1 Text** and **S7 Fig**–**S11 Fig**, including Hamiltonian replica exchange simulations (**S9 Fig** and **S10 Fig**), indicate that the GA98/GB98 switching behavior is robust over values of ε_B_ ranging from −0.30 to −0.50 (**S7 Fig** and **S8 Fig**), and that folding and switching are observed only when neither *E*_SBM_ nor *E*_trans_ vanishes (**S11 Fig**). We adopt for *E*_trans_ the implicit-solvent all-atom potential developed at Lund University (available as PROFASI), which accounts for backbone, non-bonded excluded-volume, hydrogen-bonding, charged and hydrophobic side chain interactions in a physical manner [55,56].

### The evolving dual-basin free energy landscapes of GA/GB variants

**Fig 3:**
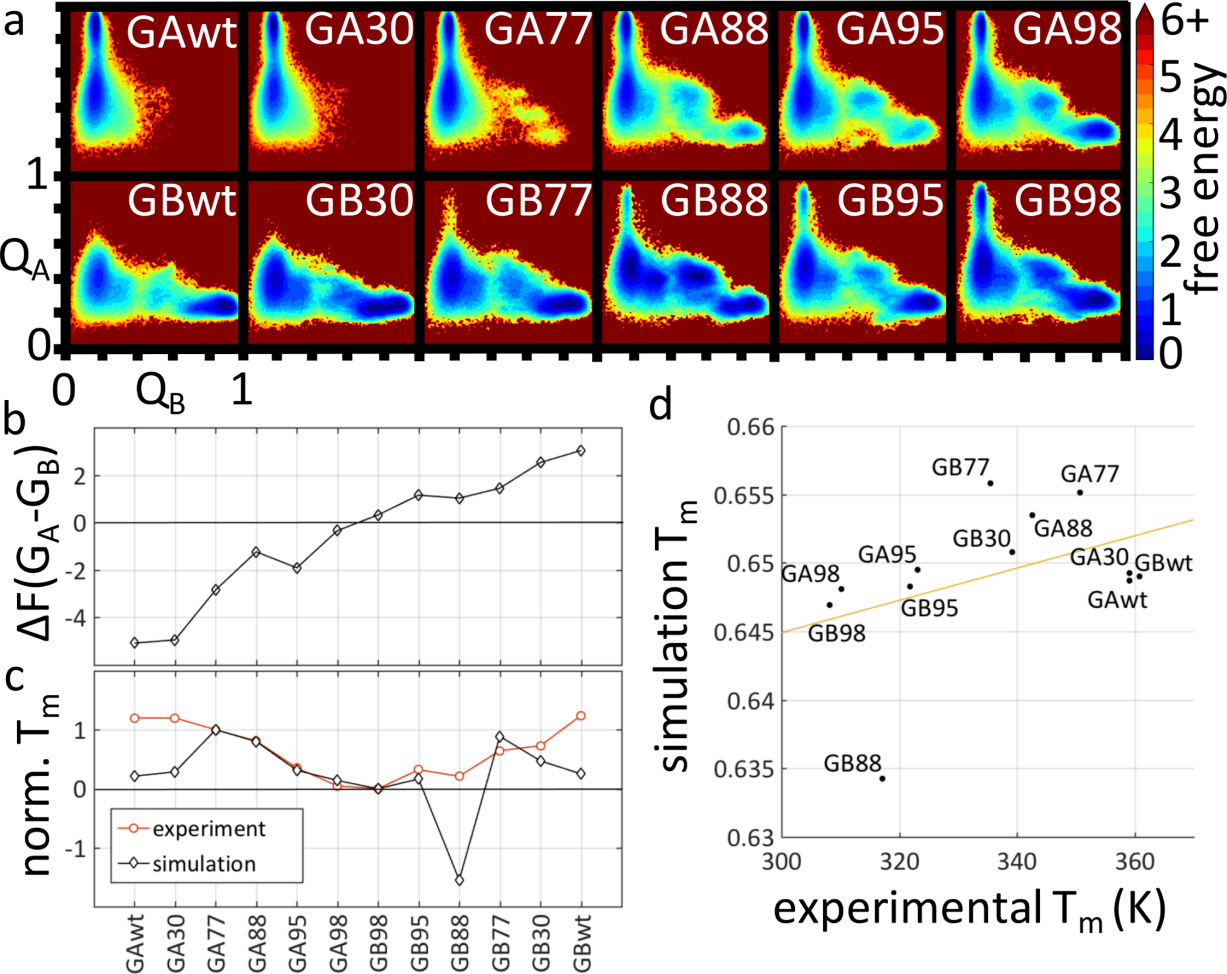
Rationalization of the G_A_/G_B_ switch and model prediction of incremental stabilization of the alternate fold. (a) Free energy landscapes as a function of the progress variables Q_A_ and Q_B_ are simulated in our hybrid model (ε_B_ = −0.37). The Q_A_/Q_B_ scale (bottom-left axes for GBwt) is identical for all GA/GB variants. Free energy, in units of *k*_B_*T*, is the negative natural logarithm of the sampled population (*Methods*). For each sequence, this quantity is computed for points on a ~100×100 grid at the sequence’s melting temperature *T*_m_. The free energies for the grid points are plotted according to the color code on the right, with the lowest free energy on the grid normalized to zero for each sequence. Note that all resulting free energy values ≥ 6 are shown in the same color. (b) Free energy differences ΔF(G_A_-G_B_). (c) Comparing sequence-dependent *T*_m_s from experiment and simulation, each normalized to the range defined by GA77 (set to 1) and GB98 (set to 0). The *T*_m_ values in (c) are in an arbitrary unit for a non-absolute temperature scale. (d) Scatter plot between absolute melting temperatures in simulation (model unit) and in experiment (in K). The experimental *T*_m_s used in the comparison in (c) and (d) are from refs. [19,20].

With a SBM component providing minimally necessary restriction on the accessible conformational space, the transferable component of our hybrid model modulates the stability of the native and unfolded populations. Using the progress variables Q_A_ and Q_B_ and the simulation procedure described in *Methods*, the present modeling setup correctly identifies the native basin of 12 sequence variants of the G_A_/G_B_ system (**Fig 3a**). The variables Q_A_≡*E*_A_/95ε_A_ and Q_B_≡*E*_B_/137ε_B_ are continuum versions of the discrete native contact fraction Q commonly used in protein folding studies [57,58]. For the energy landscapes in **Fig 3a**, the G_A_ and G_B_ native basins are situated, respectively, at Q_A_≈0.9, Q_B_≈0.15 and Q_A_≈0.3, Q_B_≈0.85; whereas the basin for the common unfolded state is centered at Q_A_≈0.4, Q_B_≈0.15. The dual native-bias of the SBM notwithstanding, **Fig 3a** shows that the transferable component is sufficiently strong to capture the physical mutational effects, resulting in significant shifts in populations and, in the case of GAwt, GBwt and GA30/GB30, virtual depopulation of the entire alternate native basin.

We computed a free energy difference ΔF(G_A_-G_B_) ≡ −ln(*P*_A_/*P*_B_) between the G_A_ and G_B_ folds for all the sequence variants (**Fig 3b**), where *P*_A_ and *P*_B_ are the populations of the two native basins defined, respectively, by Q_A_ ≥ 0.6, Q_B_ < 0.6 and Q_B_ ≥ 0.6, Q_A_ < 0.6. Thus, a negative ΔF(G_A_-G_B_) favors G_A_ whereas a positive ΔF(G_A_-G_B_) favors G_B_. The replica-exchange simulation results in **Fig 3b** show that the single L45Y mutation from GA98 to GB98 entails a small yet appreciable shift in favor of G_B_, a robust finding corroborated by constant-temperature simulations (**S12 Fig**). The aromatic Y45 partakes in a hydrophobic cluster in G_B_ but apparently contributes little to stability in G_A_ [22]. In the present transferrable potential, the hydrophobicity-based non-bonded energy term is mostly responsible for favoring this Y45-containing hydrophobic G_B_ cluster because the strength of the term scales with the number of contacting nonpolar atoms, and aromatics provide large contact areas [55]. The three mutations separating GA95 and GB95 result in a more notable population shift. In addition to L45Y, the other two amino acid substitutions are I30F leading from GA95 to GA98 and L20A leading from GB98 to GB95. Notably, the phenylalanine substitution of I30F fits into the hydrophobic core of both G_A_ (partially buried) and G_B_ (almost fully buried).

As the sequence separation between the pair is further widened (GA88/GB88, GA77/GB77, GA30/GB30, and GAwt/GBwt differ by 7, 13, 39, and 47 mutations respectively, **Fig 1**), the G_A_/G_B_ free energy difference increases. The value of ΔF(G_A_-G_B_) increases rather smoothly from GAwt to GBwt as expected. The only exception is the step from GA88 to GA95, for which there is a decrease in G_B_ propensity instead of the expected increase (**Fig 3b**). As mentioned above, for the GA30/GB30 pair, and the GAwt/GBwt pair that shares only 16% of their amino acids, the preference for the dominant native structure is so strong that only the fringe but not the bottom of the alternate native basin was sampled (**Fig 3a**). These free energy shifts are echoed by the balance between transition frequencies to and from the native basins along Monte Carlo simulation trajectories. Using a three-state division of the Q_A_/Q_B_ energy landscape into unfolded (U), G_A_, and G_B_ regions, a gradual shift from U↔G_A_ to U↔G_B_ transitions is concomitant with the sequence variation from GAwt to GBwt (**S1 Text** and **S13 Fig**).

We also compared the experimental and simulated melting temperatures of the GA/GB variants (**Fig 3c and 3d**). Because the model potential in the present hybrid G_A_/G_B_ model lacks cooperativity-enhancing desolvation barriers [59,60] and neglects temperature dependence in the solvent-mediated interactions [29,61,62], simulated and experimental *T*_m_s are not directly comparable. For example, as suggested by related kinetic trends in other protein folding models [29], insufficient folding cooperativity in the present hybrid model likely caused the simulated *T*_m_ range to be narrower than that observed experimentally (the *T*_m_ ratios of GB98 over GA77 is 0.99 for simulation and 0.88 for experiment; see **Fig 3d**). Nonetheless, for the sequence variants from GA77 to GB77, the correlation between simulated and experimental *T*_m_ is reasonably good. The consistency in *T*_m_ trend for seven of these eight variants is apparent in the comparison using a normalized non-absolute temperature scale (**Fig 3c**) as well as in the scatter plot for absolute temperatures (**Fig 3d**). The steady drop in experimental *T*_m_ from GA77 to GB98 was captured very well by simulation (**Fig 3c**). The outlier GB88 is known to be very unstable experimentally (*T*_m_ ≈ 44°C). Curiously, this effect is also reflected in our model, albeit to an exaggerated degree.

### Putative folding pathways of G_A_/G_B_ bi-stable sequences

**Fig 4:**
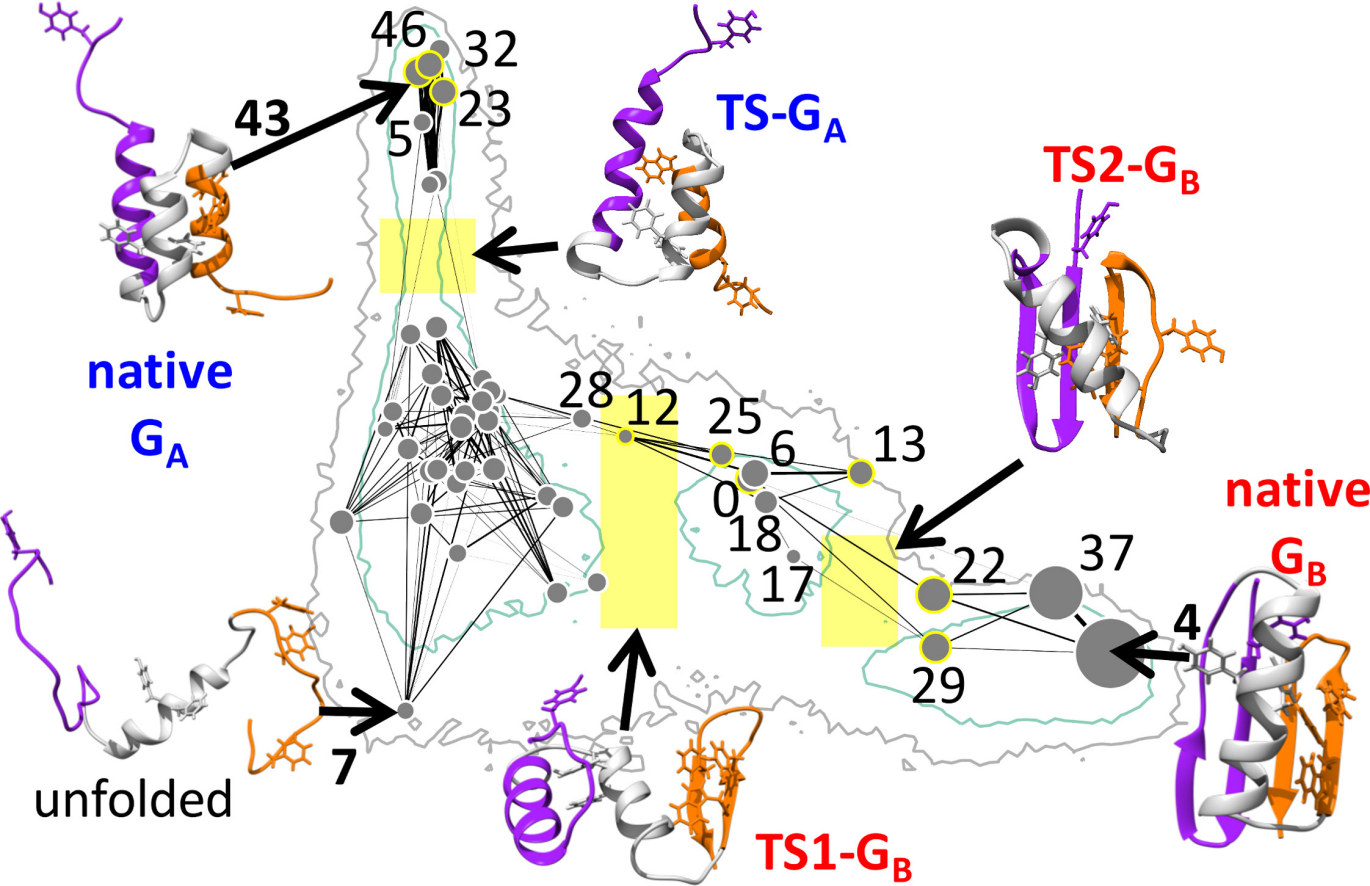
Clustering analysis of GA98 and GB98 suggests putative folding trajectories. 50*k*-means clusters were obtained from a combined pool of 40,000 randomly sampled GA98 and GB98 conformations (*Methods*). The positions of their centroids are indicated on the Q_A_/Q_B_ plane. Each centroid is represented by a grey filled circle of size commensurate with the number of conformations in the given cluster. Included in the background, as a positional reference, is the GA98 free energy landscape (**Fig 3a**). The clusters are numbered arbitrarily from 0 to 49. In the interest of readability, only number labels for selected clusters deemed to be important for the sequences’ folding pathways are shown. The centroid conformation is depicted for some clusters. A pair of centroid positions is connected by a line if their distance measure RMSD_sm_ ≤ 5.75 Å (*Methods*). Increased thickness/darkness of the connecting lines indicates that the connected centroid conformations are structurally more similar with smaller RMSD_sm_. Conformations in the yellow boxes are taken to constitute a single putative transition state TS-G_A_ for G_A_ folding and two putative transition states TS1-G_B_ and TS2-G_B_ for G_B_ folding. The TS-G_A_, TS1-G_B_, and TS2-G_B_ regions encompass, respectively, 452, 834, and 805 of the 40,000 sampled conformations. The centroid structure of each putative transition state (TS) is exhibited to illustrate the structural characteristics of the TSs; but it is important to note that the putative TS ensembles are structurally diverse (**S14 Fig**). This property is reflected by the fact that the conformations in each TS ensemble belong to multiple clusters. The three clusters (nos. 43, 46, 23) that contribute most conformations to TS-G_A_ account for only 38% of the TS-G_A_ ensemble. The three clusters that contribute most to TS1-G_B_ and TS2-G_B_ (nos. 0, 25, 12 and nos. 22, 29, 13) account for 36% and 64% of their respective ensembles. We mark each of these most TS-related clusters by a yellow ring around the grey circle representing the cluster’s centroid position. In the conformational drawings, parts of the chain corresponding to β-hairpin 1 (residues 1–20) and β-hairpin 2 (residues 42–56) in the native G_B_ fold are highlighted, respectively, in purple and orange. Shown as sticks are aromatic residues to be considered further below for possible participation in π-interactions. The positions of these aromatics also serve to highlight the locations of the main hydrophobic regions in the folded and partially folded conformations shown.

Combined structure-based clustering of the simulated GA98 and GB98 conformations allows for an analysis of likely kinetic events during bi-stable folding (*Methods*). The centroid positions of 50 conformational clusters on the Q_A_/Q_B_ landscapes are shown in **Fig 4** together with the outlines of the bi-stable GA98 free energy landscape, which is quite similar to that of GB98 (**Fig 3a**). The size of a cluster is the number of sampled conformations that are within a certain degree of structural similarity among themselves. Each centroid conformation is a representative of all the conformations in a given cluster. **Fig 4** shows that the centroid positions cover most accessible regions of the free energy landscape. Naturally, the unfolded state harbors the majority of clusters because unfolded conformations are structurally most diverse. The most extended conformations are positioned in the bottom-left region with small Q_A_ and Q_B_ values as expected (cluster no. 7). Under our model potential, there is a significant bias in favor of helical structures instead of unstructured coils in the unfolded ensemble.

As has been demonstrated, kinetic information can be gleaned from features on low-dimensional free energy landscapes determined solely by equilibrium sampling of one or two progress variables [63–65]. In using Q_A_/Q_B_ landscapes for kinetic inference, we are following this tradition. It should be noted, however, that not all kinetic properties, especially those related to kinetic trapping, are deducible from low-dimensional landscapes [45,50]. For instance, not all structurally similar conformations based on the superposition-map measure and indicated by connecting lines in **Fig 4** are readily accessible to one another kinetically. Therefore, here we qualify the “transition state” and “intermediate states” suggested by free energy landscape features as “putative”. With this caveat in view, we identify the conformations around the 0.66 < Q_A_ < 0.74, 0.12 < Q_B_ < 0.22 bottleneck region as the putative transition state for G_A_ folding. Likewise, we identify the conformations around the two bottleneck regions around 0.3 < Q_A_ < 0.55, 0.35 < Q_B_ < 0.43 and 0.28 < Q_A_ < 0.4, 0.58 < Q_B_ < 0.66 as two putative transition states for G_B_ folding (yellow boxes in **Fig 4**), and the local-minima region between the latter two transition states as a putative G_B_ intermediate state.

Along the Q_A_ direction at Q_B_ ≈ 0.15, a simple folding transition via a compact transition state TS-G_A_ is apparent in **Fig 4**. This putative process starts from an extended, mostly disordered state (cluster no. 7). Subsequently, more helices form and the chain first collapses into a loose arrangement of three helices around TS-G_A_ and then proceeds to form the ordered native G_A_ structure, with cluster no. 43 and adjacent clusters differing only by their disordered termini.

Folding along Q_B_ at Q_A_ ≈ 0.35 is more complex. **Fig 4** suggests that the second (C-terminal) β-hairpin is formed upon reaching the first G_B_ transition state TS1-G_B_, but at this stage the rest of the protein chain is still relatively open. The G_B_ intermediate state that follows consists mainly of a variety of conformations with the second β-hairpin aligned with the N-terminal β-strand. TS1-G_B_ encompasses more conformational diversity than the single centroid conformation might convey. When we partition the conformational ensemble in this region into two or more clusters (**S14 Fig**), alternative pathways across this transition region appear possible. One of the alternate pathways may entail a “mirrored” version of the second β-hairpin collapsing and accumulating as an “off-pathway” intermediate (see, e.g., the centroid conformation of cluster no. 12 in **S15 Fig**). As such, conformations with this topology likely constitute a kinetic trap that requires significant unfolding before folding to the G_B_ native state can proceed.

Direct transition from an “on-pathway” intermediate to native G_B_ is expected for those conformations with native-like orientation of the terminal secondary structure elements. To reach the second putative G_B_ transition state TS2-G_B_, excess helical structure needs to be converted into the fourth β-strand. The chain then proceeds to sample different near-native orientations of the central helix relative to the β-sheet, and attempt packing of the hydrophobic core before finally arriving at the G_B_ native state (cluster no. 4).

### Possible molecular basis for the L45Y-induced structure switch

A detailed analysis of the population shift caused by the L45Y mutation in the conformational clusters in **Fig 4** indicates that L45Y can start biasing in favor of the G_B_ structure even when the folding is in its early stage (**S1 Text** and **S15 Fig**). In this process, the aromatic-aromatic Y45-F52 interaction, which is more frequent in GB98 than in GA98, is seen as playing a significant role in the G_B_-favoring effect of L45Y (**S16 Fig**).

### Control simulations of sequence decoys and alternative switches

**Fig 5:**
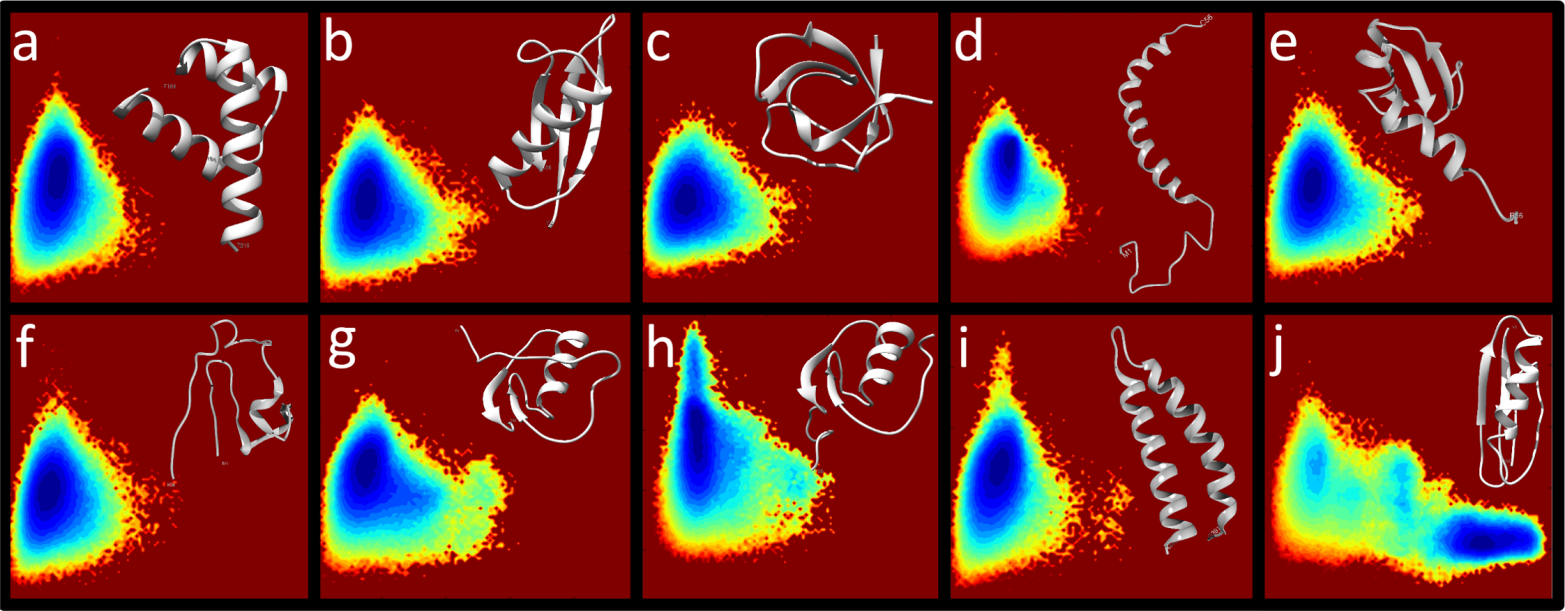
Control simulations of decoy sequences. Free energy as a function of Q_A_ and Q_B_ of various non-GA/GB sequences each with the same number of 56 residues were simulated using a hybrid potential that combines the G_A_/G_B_ SBM and a sequence-dependent transferrable component for the given sequence. The free energy landscapes are plotted in the same style as that in **Fig 3a**. PDB structures are depicted as ribbon diagrams. (a) Hox11L1 homeodomain (PDB:3A03). (b) 50S ribosomal protein LX (PDB:4V9F, chain 6). (c) Grb2 SH3C domain (PDB:2VVK). (d) Peripheral stalk subunit H of the methanogenic A1AO ATP synthase (PDB:2K6I). (e) N-terminal domain of ribosomal protein L9 (PDB:1CQU). (f) Rubredoxin type protein from Mycobacterium ulcerans (PDB:2M4Y). (g) Pancreatic secretory trypsin inhibitor (Kazal type) variant 3 (PDB:1HPT). (h) Serine protease inhibitor infestin 4 (PDB:2ERW). (i) Ral binding domain of RLIP76 (PDB:2KWH). (j) Modified 56-residue version of protein L (PDB:2PTL; see Methods).

As a test of the robustness of our hybrid model, we challenged it by several other sequences from the PDB that have the same 56-residue chain length as the GA/GB sequences but with native folds different from either G_A_ or G_B_. The same G_A_/G_B_ SBM was applied with each sequence’s Lund potential used as the transferrable component. The goal is to ascertain whether these decoy sequences would mistakenly adopt the G_A_ or G_B_ fold. Seven of the decoy sequences tested behaved reassuringly. Despite the G_A_/G_B_ SBM, they did not populate either of the G_A_/G_B_ native basin, even though some of their native conformations have secondary structures similar to those of G_A_ or G_B_ (**Fig 5a–5g**). This result shows that *E*_trans_ can override *E*_SBM_, underscoring that the transferrable physical potential plays a highly significant, if not dominant, role in our model.

Among the decoys tested, serine protease inhibitor infestin 4 is an interesting exception because its native structure is not similar to G_A_ but it populates the G_A_ basin (**Fig 5h**); but the bulk of its conformations remain unfolded. In this regard, depopulation of both native basins is remarkable for the double helical Ral binding domain because its helical secondary structures are similar though not identical to that of G_A_ (**Fig 5i**).

Finally, to test whether our model can fold a non-GA/GB sequence if its native fold is essentially identical to either G_A_ or G_B_, we considered a modified 56-residue version of Protein L (*Methods*). Protein L has only ~ 16% sequence identity with GBwt but adopts the overall G_B_ fold experimentally. Reassuringly, our simulation shows that the modified Protein L sequence is compatible with the G_B_ basin but not the G_A_ basin (**Fig 5j**).

**Fig 6:**
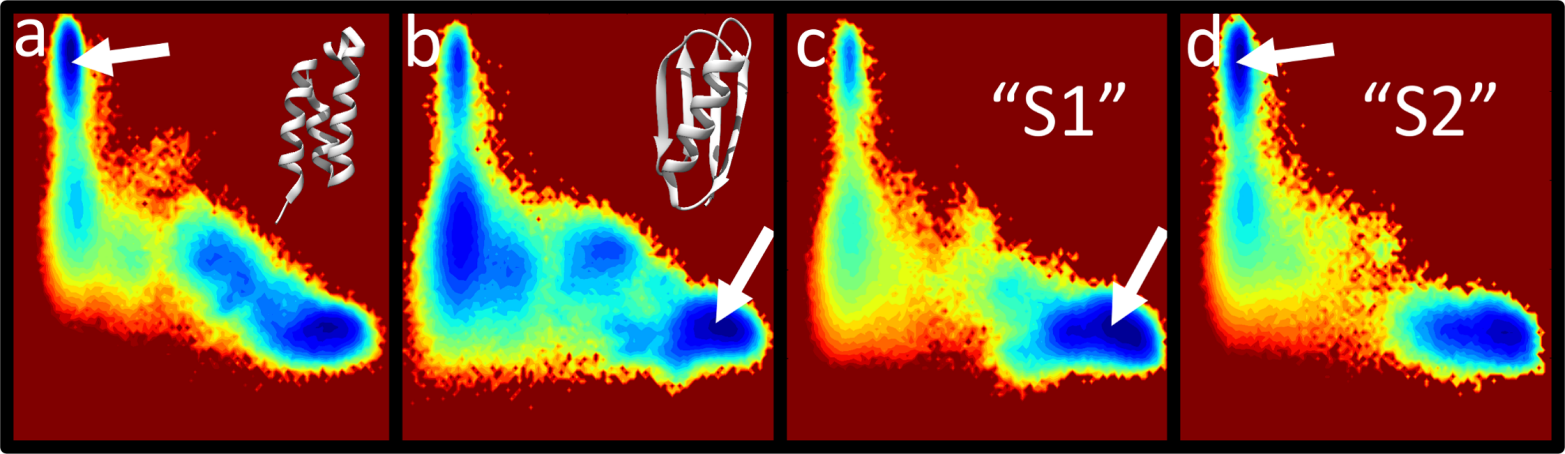
Free energy landscapes of additional switch sequences in the G_A_/G_B_ system. Plotted in the same style as that in **Fig 5**. (a) GB98-T25I (PDB:2LHG). (b) GB98-T25I,L20A (PDB:2LHE). PDB structures in (a) and (b) are depicted by the ribbon diagrams. (c) Predicted switch sequence “S1” prefers G_B_ whereas (d) sequence “S2” prefers G_A_ [66]. The arrows mark the global minimum in each of the energy landscapes.

Apart from decoys, we also challenged our formulation with an alternative structure switch in the G_A_/G_B_ system discovered more recently. Experiments indicate that the T25I mutant of GB98 reverts back to the helical structure of the G_A_ folds, but with an additional L20A mutation can be restored to the G_B_ fold [22]. Our simulations show a high degree of bi-stability for these two sequences as for GA98 and GB98. Nonetheless, we also found a small free energy difference that is consistent with the experimentally observed native structures of these two variants (**Fig 6a and 6b**). Another pair of possible GA/GB switch sequences that came to our attention was proposed recently [66], but the predicted switching behavior has not been confirmed by experiment or investigated by explicit-chain modeling. Our simulations here are in agreement with the predictions in finding that sequence “S2” prefers the G_A_ fold while “S1” prefers the G_B_ fold (**Fig 6c and 6d**). The free energy differences for these two alternative switch mutations are provided in **S17 Fig**. Our results suggest that GB98-T25I,L20A and S1 favor G_B_ via different mechanisms. GB98-T25I,L20A predominantly stabilizes the entire unfolded state and parts of the G_B_ native state yet leaving the native G_A_ basin appreciably populated (**Fig 6b**), whereas the S2 to S1 mutation P54V clearly destabilizes the G_A_ fold (**Fig 6c**).

### Sharpness of conformational switch can be enhanced by π-π interactions among aromatic residues

**Fig 7:**
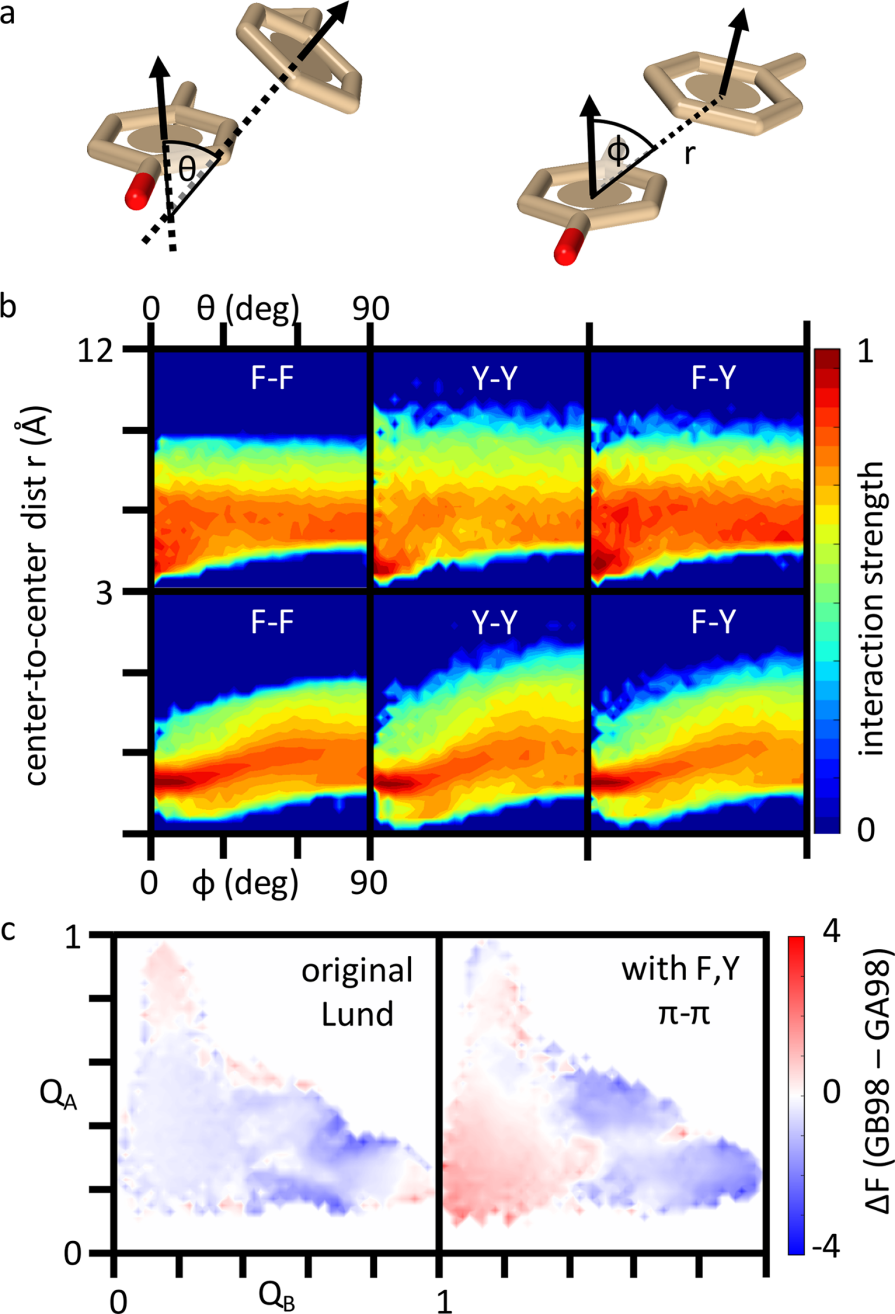
Effect of a rudimentary π-π potential for Phe (F) and Tyr (Y) residues on the G_A_/G_B_ switch. (a) Three geometric variables are used to characterize the relative position and orientation of a pair of aromatic rings in F or Y: center-to-center distance *r* (spatial separation between the centers of the two rings), planar tilt angle *θ*, and center dislocation angle *φ*. (b) PDB statistics for F-F, Y-Y, and F-Y contacts were used to derive an interaction strength of the π-π potential as a function of *r*, *θ*, and *φ*. The vertical variable here corresponds to |*E*_ππ_(*r*, *θ*, *φ*)/*ε*_ππ_| defined in *Methods*. (c) Difference landscape for the L45Y mutation. Free energy of GB98 minus free energy of GA98 as a function of Q_A_ and Q_B_ computed in our hybrid model with two different transferrable components: the original Lund potential (left), and the modified potential (right) that incorporates the F,Y potential with interaction strengths given in (b) and *ε*_ππ_ = 1.5.

The analysis of the L45Y mutation in **S15 Fig** reveals that a major part of its stabilizing effect on the G_B_ fold is through enabling the aromatic-aromatic Y45-F52 interaction in GB98. In view of this observation and the general importance of π-related interactions in biomolecular processes [49,67], we constructed a rudimentary π-π interaction potential for F and Y residues (*Methods*). Our goal here is to explore how an orientation-dependent interaction between aromatics that goes beyond simple hydrophobic effects may affect the behavior of the G_A_/G_B_ conformational switch, although a comprehensive study of aromatic interactions is beyond the scope of this work. By using three geometric variables for two neighboring aromatic rings (**Fig 7a**), we derived an empirical π-π potential [68] for F and Y from PDB statistics (**Fig 7b**). When this π-π potential replaces the simpler hydrophobic interactions among F and Y residues in the original Lund potential, the effect of L45Y is affected appreciably (**Fig 7c**).

We define a difference landscape for the original Lund potential (**Fig 7c**, left) as the difference between the GA98 and GB98 panels in **Fig 3a**. The difference landscape for the modified transferrable potential (**Fig 7c**, right) is similarly defined using the Q_A_/Q_B_ landscapes of GA98 and GB98 in **S18 Fig** that incorporates our π-π potential. In the Lund potential (**Fig 7c**, left), L45Y stabilizes the unfolded and G_B_ intermediate states rather homogeneously (stabilization indicated by blue coloring). The G_A_ native basin is destabilized (red coloring), but so are parts of the G_B_ native basin. In contrast, with the π-π potential (**Fig 7c**, right), L45Y has a stronger impact. It now destabilizes most of the unfolded state and parts of the G_A_ native basin whereas the stabilization focuses more on the intermediate and native basins of G_B_. Although the present π-π potential is rudimentary, this comparison suggests that orientation-dependent π-π interactions likely play a significant role in the experimental sharpness of the G_A_/G_B_ conformational switch.

## Discussion

To recapitulate, we showed that a coarse-grained Cα SBM in combination with an all-atom transferable potential correctly identifies the native state of an extensive set of GA and GB sequence variants. As shown above, our hybrid model is well suited for exclusively selecting the correct native state for GA/GB pairs of up to 77% identity. At higher sequence similarity, both folds were populated in our simulations; but a clear preference consistent with experiment was observed.

### Further comparison with experiment

Beside this overall success, two findings from our investigation are of experimental relevance: (i) existence of an equilibrium intermediate for G_B_ folding (**Fig 3a**, GB panels); and (ii) a critical role of the second β-hairpin in the G_B_ folding pathway (**Fig 4 and S15 Fig**). On both counts, our model results are in general agreement with experimental findings (see below), lending additional credence to our contention that the present hybrid model is capable of capturing essential physics of G_A_/G_B_ bi-stability and the GA98/GB98 conformational switch.

Firstly, our prediction of a GBwt (also called protein G or GB1) intermediate is in line with several [69–72] though not all [73] simulation studies. Experimental evidence for a G_B_ folding intermediate was presented, but there is no clear consensus yet regarding the existence and/or nature of a G_B_ intermediate–unlike the generally recognized two-state nature of G_A_ folding. Two early experiments oncluded that GBwt folding is two-state [74,75]. In contrast, another early continuous-flow ultrarapid mixing experiments on GBwt suggested a native-like intermediate [76], but this conclusion was disputed [77]. A later FRET study also found an intermediate near the urea denaturation midpoint of GBwt [78]. A subsequent equilibrium GBwt unfolding experiment showed two-state behavior; but the kinetic chevron rollover was indicative of an intermediate [79]. The latter finding is in line with a recent experimental and molecular dynamics study showing that GBwt folding is three-state [80]. As for GB variants, one study found that GB88 and GA88 are two-state folders [21]. However, an investigation on a different set of variants GA30/GB30, GA77/GB77, and GA88/GB88 supported three- and two-state folding, respectively, for all GB and GA variants [81]. Taken together, recent evidence appears to be somewhat more preponderant on the existence, rather than non-existence, of a G_B_ folding intermediate; and is unequivocally affirmative of the two-state nature of G_A_ folding. This trend is reflected by our simulated free energy landscapes in **Fig 3a**.

Secondly, **Fig 4** and **S15 Fig** suggest that the second β-hairpin is critical and more important than the first β-hairpin in G_B_ folding. Although this finding was deduced from an analysis of GA98 and GB98 clusters, it is likely applicable to other GB variants, including GBwt, because of the similarity among their free energy landscapes (**Fig 3a**). Indeed, NMR experiments on peptides from GBwt found that, in isolation, the second β-hairpin is much more stable than both the helix and the first β-hairpin. It forms a stable, native-like β-hairpin with its three aromatic residues W43, Y45, and F52 forming a cluster stabilized by both hydrophobic and (probably π-related) polar interactions [82]. In contrast, the first hairpin was found to be mostly flexible in isolation and not native-like [83]. Hydrogen exchange experiments on the entire GBwt protein also revealed an early folding state with the second β-hairpin having the highest protection factors, whereas the helix has a lower and the first hairpin has the lowest [77,84]. Based on Φ-value analysis for a single transition state, another study also pointed to the presence of the second β-hairpin in the GBwt transition state [74]. Taken together, the experimental data summarized above provide support for a critical role of Y45-F52 in favoring early formation of the second β-hairpin and its partial collapse together with the helix, as suggested by our simulation (compare TS1-G_B_ in **Fig 4** and **S14 Fig**).

In this regard, some differences between the folding transition states of GB variants and that of GBwt were reported. In particular, Φ-value analysis [85] has found that the first transition state in GB30 is more sensitive to mutations in the second β-hairpin whereas GB88 is more sensitive in the first hairpin [81]. Nonetheless, the same set of data for GB88 is suggestive of native-like transition-state contacts, such as I6-T53, that are between strands at the two termini because some of their residues have high Φ-values (e.g., 0.48 for I6 and 0.42 for T53). If this is indeed the case, the experimental data is not inconsistent with our simulation result suggesting that the anti-parallel alignment of the termini is an early rate-limiting event for G_B_ folding (**Fig 4** and **S15 Fig**).

### Outlook

Taking all the evidence presented together, the performance of our model suggests that the remarkable G_A_/G_B_ bi-stability phenomenon can be rationalized to a significant extent by specific hydrophobic interactions, though our physical understanding is still far from complete. As discussed above, future improvement in matching theory with experiment should be sought by enhancing folding cooperativity and increasing sharpness of the conformational switch in our model. One possible direction is to incorporate desolvation barriers in the transferrable potential because this is a robust physical feature of solvent-mediated interactions that have a significant impact on folding cooperativity [29]. Another direction, which was initiated with some success here, is to devise a more accurate description of aromatic interactions [67]. In this respect, a natural next step is to extend our model π-π interactions to encompass Trp and to adopt a more comprehensive account of the relative position and orientation of interacting aromatic sidechains that goes beyond the three variables in **Fig 7**.

Despite the simplicity of the Lund potential, it has succeeded in folding several smaller proteins [55,86] and the 92-residue Top7 [87]. However, in long unbiased folding simulations using only the Lund potential with no SBM, we were unable to observe stable native-like conformations of GA/GB variants, indicating that as-yet-unknown energetic contributions, in addition to those in the Lund potential, are needed for a complete physical account. The G_A_/G_B_ system is a useful benchmark for testing forcefields and simulation techniques. Recent success in using all-atom explicit-water molecular dynamics to simulate folding of a number of small proteins is remarkable [88–90]. However, despite the notable advance and ongoing force-field improvement [23,91], no *ab initio* forcefield to date has been able to fold the GA/GB variants correctly [25].

In this context, hybrid modeling is a highly useful interim approach to gain physical insight into protein folding energetics, effects of mutations, and to assist in protein design. Owing to its reliance on SBMs, this approach is limited to proteins with known structures. Nonetheless, for many globular proteins, the native structure is either known or can be inferred through homology or sequence-based statistical models [92,93], and are therefore amenable to hybrid modeling. Common approaches to estimate mutational ΔΔ*G* [94] only consider the known native structure with little or no regard to the unfolded state and folding dynamics. Hybrid models can address this shortcoming by providing testable predictions about the mutational effects on the entire free energy landscape. Indeed, because of its computational tractability, hybrid models can facilitate efficient development and testing of physically more accurate transferrable potentials, and thus can contribute to an ultimate elimination of the current necessity for SBMs.

## Methods

### Consensus contact maps for multiple native conformers and multi-well contact potentials

As described above in Results, we derived for the native-centric SBM component of our hybrid model two consensus native contact maps that capture the general features of the G_A_ and G_B_ folds by using PDB structures for four GA sequence variants and four GB sequence variants (**Fig 2a and 2b**). The sequences and their corresponding structures (in parentheses) are GAwt (2FS1), GA88 (2JWS), GA95 (2KDL), GB98 (2LHC), GBwt (1PGA), GB88 (2JWU), GB95 (2KDM), and GB98 (2LHD). All of these PDB structures except the x-ray structure for GBwt were determined using NMR and contain multiple model structures. For simplicity, we used only the first model in each NMR PDB file in our analysis. Assuming that these consensus contact maps provide a reasonable coverage of the structural variations in the G_A_/G_B_ system, we apply these maps to sequence variants GA30, GB30, GA77, and GB77 as well, since no detailed structural data were available for the latter four sequences [20].

We introduce *E*_A_ and *E*_B_ as the individual native-centric potential energy functions for the G_A_ and G_B_ folds, respectively. *E*_A_ and *E*_B_ depend on the Cα-Cα distances *r*_*ij*_ for all residue pairs *i*,*j* that belong to the given consensus native contact map via the following Gaussian form [53]:

$$E_{A}=\varepsilon_{A}\sum_{i,j}^{n_{A}}\left[\prod_{s}^{n_{s}}\left(1-e^{-{\left(r_{ij}-d_{ij}^{\left(s\right)}\right)}^{2}/2w^{2}}\right)-1\right],$$

and a similar equation for *E*_B_ with all instances of “A” replaced by “B”. Here the summation over *i*,*j* for *E*_A_ and *E*_B_ runs over, respectively, all *n*_A_ = 95 and *n*_B_ = 137 contacts in the consensus contact maps for G_A_ and G_B_. The product over *s* takes into account the multiple native distances $d_{ij}^{\left(s\right)}$ for residue pair *i*,*j* in the *n*_s_ = 4 PDB structures contributing to the consensus map. The strength of *E*_A_ or *E*_B_ is given, respectively, by ε_A_ or ε_B_, which corresponds to the well depth for a single native contact. The *w* parameter that controls well width is set at 0.5 Å. In the present study, this formulation leads to a wide potential well for an overwhelming majority of consensus contacts. Because in most cases the native Gaussian wells for individual structures overlap considerably, we observe only minor barriers between individual Gaussian minima among all the consensus native potentials shown in **S1 Fig** and **S2 Fig**. In **Fig 2c and 2d**, examples of the consensus potential $E_{ij}=\prod_{s}^{n_{s}}\left\{1-\exp\left[-{\left(r_{ij}-d_{ij}^{\left(s\right)}\right)}^{2}/2w^{2}\right]\right\}$ for an individual contact (black curves) are provided together with the corresponding energy term $1-\exp\left[-{\left(r_{ij}-d_{ij}^{\left(s\right)}\right)}^{2}/2w^{2}\right]$ for one of the four contributing PDB structures (color curves).

The above Gaussian form of the native-centric energy function is more suitable than the Lennard-Jones (LJ) form for our present purpose. As has been noted, it is difficult to produce a viable combined energy function from multiple native-centric LJ functions for multiple structures unless the conformational diversity is approximated by a single centroid structure [95]. LJ potentials are inflexible in their well shape (width). Each inter-residue contact comes with a built-in repulsion term determined by the minimum-energy contact distance in LJ. As a result, multiple instances of the same contact at varying distances can lead to occlusion of the shorter-range contact by the repulsion of the longer-range contact if the LJ form is used instead of the Gaussian form to construct a combined energy function in accordance with the equation above (**S1 Fig** and **S2 Fig**).

### Native bias for two alternate folds and the transferrable component in the hybrid model

As outlined above, the total potential energy *E*_total_ is the sum of a native-centric component and a transferrable component, viz.,. *E*_total_ = *E*_SBM_ + *E*_trans_. The dual-basin native-centric SBM component *E*_SBM_ is constructed simply as *E*_SBM_ = *E*_A_ + *E*_B_. Aiming to increase the weight of the transferrable component in our model potential, we did not employ the more native-specific prescription of logarithmic mixing in ref. [35] for *E*_SBM_. For the transferrable component *E*_trans_, we adopt the Lund potential: *E*_trans_ = *E*_local_ + *E*_EV_ + *E*_HB_ + *E*_SC_ + *E*_HP_, where the energy terms on the right are for local backbone interactions (*E*_local_), non-bonded excluded volume (*E*_EV_), hydrogen bonds (*E*_HB_), charged (*E*_SC_) and hydrophobic (*E*_HP_) sidechain interactions. Bond lengths and bond angles are kept constant, as described by the original authors [55]. Dimensionless energy units are used in our simulations with Boltzmann constant *k*_B_ effectively set to unity.

### Monte Carlo simulations

We use a Monte Carlo (MC) [96] package [56] from Lund University to conduct parallel tempering (temperature replica exchange) MC simulations [97]. MC chain moves included backbone and side chain rotations as well as biased Gaussian steps [98]. All simulations were initialized from random chain conformations and time propagated in units of MC cycles. Each cycle consisted of a number of elementary conformational MC updates scaled to the number of rotational degrees of freedom of the simulated protein chain so that on average all degrees of freedom were perturbed once per cycle. For example, for GA98 and GB98 these numbers of degrees of freedom were 283 and 282, respectively.

Initially, parallel tempering simulations were performed over 32 replicas per simulation over a wide temperature range. This is then followed by a second simulation using a finer temperature grid around the melting (unfolding) temperature, *T*_m_, determined as the temperature at which the heat capacity function

$$C_{V}(T)=\frac{1}{k_{B}T^{2}}\left({\left\langle E_{total}{}^{2}\right\rangle }_{{}_{T}}^{}-{\left\langle E_{total}\right\rangle }_{{}_{T}}^{2}\right)$$

computed from the first set of simulations attains its maximum. Here *T* is absolute temperature of the simulation, *E*_total_ is the total energy defined above, and <…>_*T*_ denotes conformational averaging at *T*. The refined temperature grid was tuned to ascertain sufficient replica exchange acceptance probability around *T*_m_ (~99%). Replica exchange was attempted every 5,000 MC cycles, the first 30% (3.0×10^6^ MC cycles) of every trajectory was excluded from analysis. Populations simulated at different temperatures were reweighted to *T*_m_ using WHAM [99]. In select instances, 128 constant-*T* simulations at *T*_m_ with increased sampling were conducted to corroborate parallel tempering results (**S5 Fig** and **S12 Fig**). In view of the need for a high computational throughput for varying input parameters and sequences, most simulations were terminated after 10^7^ MC cycles (~2.8×10^9^ elementary MC updates). We verified that the resulting simulated ΔF(G_A_‒G_B_) for GA98 and GB98 is reasonably robust in longer simulations.

### Clustering of sampled conformations

From the replica exchange simulations around *T*_m_ for GA98 and GB98, for each sequence we randomly sampled 20,000 conformations obtained at the two sequences’ respective *T*_m_s. These conformations were combined into a single pool of 40,000 conformations for clustering analysis. Each conformation in the pool was represented as a (4×56)-dimensional vector. The first 56 and second 56 components of this vector are the distances between the Cα atoms in the given conformation and the corresponding Cα atoms, respectively, of an optimally superposed GA98 PDB structure (2LHC) and an optimally superposed GB98 PDB structure (2LHD). Similarly, the third 56 and fourth 56 components of the (4×56)-dimensional vector are the distances between the Cβ atoms in the given conformations and the corresponding Cβ atoms in the optimally superposed PDB structures, respectively, for GA98 and GB98. Structural superpositions were optimized using the MDtraj [100] implementation of Theobald’s algorithm for RMSD calculations [101]. The (4×56)-dimensional distance vectors were then clustered by the *k*-means algorithm [102] with *k* = 50 chosen as the number of clusters. Cluster centroids are defined as actual conformations situated closest to the cluster centers in the (4×56)-dimensional space.

We define a distance measure between the centroids of two conformational clusters as the Cartesian distance between the centroids’ (4×56)-dimensional vectors normalized by (4×56)^1/2^. We refer to this distance measure as RMSD_sm_ because it is the root mean square difference of the centroids’ superposition maps, RMSD_sm_. The latter is defined for any pair of conformations *C*_*μ*_ and *C*_*ν*_ as

$$RMSD_{sm}\left(C_{\mu },C_{\nu }\right)=\sqrt{\frac{1}{4\times 56}{\sum_{i=1}^{4\times 56}\left(d_{i}^{\left(\mu \right)}-d_{i}^{\left(\nu \right)}\right)}^{2}}$$

where $d_{i}^{\left(\mu \right)}$ and $d_{i}^{\left(\nu \right)}$ are the components of the (4×56)-dimensional vectors representing, respectively, conformations *C*_*μ*_ and *C*_*ν*_. RMSD_sm_ was first used in the general clustering for all conformations. For **Fig 4**, the general definition was applied to pairs of cluster centroids, wherein only pairs with RMSD_sm_ ≤ 5.75 Å are shown by connecting lines. This threshold was chosen solely for the presentational purpose of not obstructing the visualization in **Fig 4** yet providing as much information as possible about the structural relationships between clusters that share a reasonable degree of geometric similarity.

### Design of a 56-residue version of protein L for a control simulation

The sequence of the modified version of protein L in **Fig 5** was obtained by first structurally aligning its PDB structure (2PTL) with that of GB1 (1PGA) and then removing the unaligned N- and C-terminal tails. Internal loop residues 12, 40, 41, and 42 were also removed and a glycine was inserted between residues 23 and 24. This procedure led to the following sequence used in **Fig 5**: VTIKANLIFANSTQTAEFKGTFAEKATSEAYAYADTLKKEYTVDVADKGYTLNIKF.

### Rudimentary statistical potential for π-π interactions among Phe and Tyr residues

Interactions between aromatic residues in the Lund potential are treated only by its hydrophobic side chain potential [55]. To explore possible π-interactions that are not hydrophobic in nature but are nonetheless known to play significant structural roles in biomolecules [49,67,103,104], we modified the Lund potential for Phe and Tyr, replacing their contact-area-dependent hydrophobic interactions by an orientation-dependent potential. This rudimentary π-π potential is parametrized by three geometric variables *r*,*θ*,*φ* characterizing the relative position and orientation of two aromatic rings (**Fig 7a**). There is one Trp in the GA/GB sequences (W43); but for simplicity we restrict our exploration to Phe and Tyr, leaving the treatment of the geometrically more complex Trp to future studies. The present π-π interaction is derived as a statistical potential from a PDB data set obtained through the PDB-SELECT [105] repository at http://swift.cmbi.ru.nl/gv/select/index.html. The sequence similarity cut-off was 30%, R-factor cutoff was 0.21, and resolution cut-off was 2.0 Å. The dataset contained 9,796 protein crystal structures (created on January 26, 2013). For all the observed F-F, Y-Y, and F-Y contact pairs in this data set, the number of occurrences *P*(*r*,*θ*,*φ*) of *r*,*θ*,*φ* were distributed into bins of size 0.3 Å for *r* between *r* = 3 Å and 12 Å and bins of size 3° for *θ*,*φ* between *θ*,*φ* = 0° and 90°. Based on this statistics and following Procacci and coworkers [68], we define a rudimentary π-π interaction energy *E*_ππ_(*r*,*θ*,*φ*) = −*ε*_ππ_{1+ln[*P*(*r*,*θ*,*φ*)/*P*_max_]/|ln(*P*_min_/*P*_max_)|} for each of the three residue type pair F-F, Y-Y, or F-Y, where *P*_max_ and *P*_min_ are, respectively, the maximum and minimum non-zero values of *P*(*r*,*θ*,*φ*) among all the bins for a given pair. We further set *E*_ππ_ = 0 for all *r*,*θ*,*φ* bins that received zero entry from the PDB data set. In this way, for *ε*_ππ_ > 0, the present π-π potential is an attractive interaction that varies between *E*_ππ_ = −*ε*_ππ_ and 0 (**Fig 7b**). Here we use *ε*_ππ_ = 1.5 for all three residue type pairs.

## Supporting Information

S1 Fig Multi-well Gaussian contact potentials for 95 consensus G_A_ native contacts. The contacts are numbered arbitrarily from 1 to 95. Residue pairs of the contacts are in parentheses. Here contact energy *E*_*ij*_ (in units of ε) is a function of Cα-Cα distances *r*_*ij*_ (in Å). For each contact, a single Gaussian multi-well potential derived from the corresponding Cα-Cα distances *d*^*(s)*^_*ij*_ in the PDB structures of four GA sequences is in blue. For comparison, four separate Lennard-Jones (LJ) potentials 4ε[(*d*^*(s)*^_*ij*_/*r*_*ij*_)^12^ − (*d*^*(s)*^_*ij*_/*r*_*ij*_)^6^] with the same native Cα-Cα distances and well depth ε are shown in red, and the linear combination of the four Lennard-Jones potentials Σ_s_ ε[(*d*^*(s)*^_*ij*_/*r*_*ij*_)^12^ − (*d*^*(s)*^_*ij*_/*r*_*ij*_)^6^], each with well depths scaled down to ε/4, is in green. Details of our construction of multi-well Gaussian contact potentials are given in *Methods* of main text.
(TIFF)

S2 Fig Multi-well Gaussian contact potentials for 137 consensus G_B_ native contacts. Same as **S1 Fig** but here the potentials were derived from the known folded structures of four GB sequences.
(TIFF)

S3 Fig Effect of SBM strengths on simulated free energy landscapes in the hybrid model. Free energy as function of Q_A_ and Q_B_ was computed using replica exchange for GA98 (left) and GB98 (right) for different ratios of SBM energies for G_A_ and G_B_ at the different models’ respective *T*_m_s, with ε_B_ = −1 throughout. (a,b) The G_A_ and G_B_ SBM basins have the same minimum energy, viz., min(*E*_A_) = min(*E*_B_). (c,d) The individual native contact strengths in G_A_ and G_B_ are identical, i.e., ε_A_ = ε_B_.
(TIFF)

S4 Fig Sequence-dependent native preference ΔF(G_A_-G_B_) at the models’ respective *T*_m_s for four different SBM strengths ε_B_. Results for the ‒ε_B_ values tested are shown in different color as indicated, with ε_A_ = 0.96ε_B_ throughout. Negative or positive value along the vertical axis indicates how much the thermodynamic equilibrium is biased, respectively, toward the G_A_ or G_B_ native state.
(TIFF)

S5 Fig Free energy landscapes of bi-stable sequences from constant-temperature simulations with a weak SBM potential. Free energy as a function of Q_A_ and Q_B_ was simulated for GA98 (a) and GB98 (b) at each sequence’ respective *T*_m_ and ε_B_ = −0.25. For each sequence, 128 independent trajectories were simulated over 10^7^ Monte Carlo cycles. The free energy for each sequence was computed from the sampled population as a whole after discarding the first 30% of every trajectory.
(TIFF)

S6 Fig Contributions from different energy terms in the potential function for the hybrid model. Shown here as examples are energies averaged over sampled GB98 conformations in the unfolded state (yellow columns) and in the G_B_-folded state (green columns) simulated using ε_B_ = −0.37. The unfolded state is defined by Q_A_ ≤ 0.6 and Q_B_ ≤ 0.3, the G_B_ folded state is defined by Q_B_ > 0.7. The columns and numbers show the average total energy (*E*_total_) and its contributing averages from various energy terms, the error bars mark the ranges of energies sampled. As defined in *Methods* of main text, *E*_SBM_ is the dual SBM term *E*_A_+*E*_B_, which is seen to have a minor stabilizing contribution (negative value) compared to the sum of transferrable terms such as the torsion-related (*E*_local_), hydrogen-bonding (*E*_HB_), and hydrophobic (*E*_HP_) terms. Because of a large repulsive contribution from the transferrable excluded-volume term (*E*_EV_) and an almost neutral contribution from charged side-chain interactions (*E*_SC_), the average total energy *E*_total_ is positive. Further details are provided in *Methods* of main text and Irbäck et al., 2009 cited in **S1 Text**.
(TIFF)

S7 Fig Temperature replica exchange simulations of GA98 and GB98 under varying −ε_B_, from 0.2 to 0.5. Q_A_ vs Q_B_ free energy landscapes obtained after reweighting to the respective *T*_m_. Simulation procedure and plotting style are the same as that described for **Fig 3a** of main text.
(TIFF)

S8 Fig Dependence of the native state preference ΔF(G_A_−G_B_) and melting temperature *T*_m_ (inset) on the SBM strength −ε_B_ for the simulations of GA98 and GB98 in S7 Fig. Note that the G_A_ fold is always favored more by GA98 than by GB98, whereas the G_B_ fold is always favored more by GB98 than by GA98.
(TIFF)

S9 Fig Free energy landscapes computed using Hamiltonian replica exchange simulations of GA98 with varying −ε_B_ among replicas. Simulations were conducted at the constant temperature shown at the top. The landscapes are depicted in the same style as that in **S7 Fig**.
(TIFF)

S10 Fig Free energy landscapes computed using Hamiltonian replica exchange simulations of GB98 with varying −ε_B_ among replicas. Simulations were conducted at the constant temperature shown at the top. The landscapes are depicted in the same style as that in **S9 Fig**.
(TIFF)

S11 Fig **Free energy landscapes computed by simulations of GA98 and GB98 (a) using only the Lund potential without SBM, and (b) with all long-range interactions in the Lund potential turned off, but with the SBM on.** In (a), the Q_A_ and Q_B_ reaction coordinates were based, respectively, on the 2LHC and 2LHD PDB structures. In (b), −ε_B_ was either 0.37 (top) or 1 (bottom). A wide temperature grid was used for temperature replica exchange to sample both folded and unfolded conformations. Free energy in each panel is plotted in units of *k*_B_*T* according to the scale on the right.
(TIFF)

S12 Fig Free energy landscapes of bi-stable sequences from constant-temperature simulations. Free energy as a function of Q_A_ and Q_B_ was simulated for GA98 (a) and GB98 (b) at each sequence’s respective *T*_m_ and ε_B_ = −0.37. For each sequence, 128 independent trajectories were simulated over 10^7^ Monte Carlo cycles. The free energy for each sequence was computed from the sampled population as a whole after discarding the first 30% of every trajectory. This calculation gives ΔF(G_A_-G_B_) = −0.8 for GA98 and ΔF(G_A_-G_B_) = +0.77 for GB98.
(TIFF)

S13 Fig Transition fluxes among macroscopic states during Monte Carlo sampling. For this analysis, conformations in the Q_A_/Q_B_ energy landscapes are divided into three (a) or eight (b) macroscopic states. Transition frequencies between these states during Monte Carlo sampling were recorded. Normalized two-way transition frequencies shown here are for (c) G_A_ (folded) and U (unfolded), G_B_ (folded) and U (unfolded) in the case of three macroscopic states; (d) all neighboring states, and (e) G_A_ and its neighboring transition state as well as G_B_ and its neighboring transition state in the case of three macroscopic states (d,e). Data are provided here for all twelve GA/GB sequence variants in (a) and (c); but only for eight variants in (b) for which the transitions of interest were observable during our simulations.
(TIFF)

S14 Fig Conformational diversity in putative transition-state ensembles. Using the same conformational similarity measure for the *k*-means clustering of all accessible conformations (*Methods* of main text), a *separate* clustering of each of the three putative transition states, (a) TS-G_A_, (b) TS1-G_B_, and (c) TS2-G_B_, was performed for the (a) 453, (b) 834, and (c) 805 sampled conformations, respectively, in the yellow boxes in **Fig 4** of main text that defined these states. Each of the putative transition states was partitioned into three clusters (*k* = 3). The centroid conformations of the clusters are shown here with the percentages of conformations the clusters encompass.
(TIFF)

S15 Fig Mutation-induced population shift caused by L45Y from GA98 to GB98. Clusters of conformations presented in **Fig 4** of main text were further analyzed. The manner in which cluster size and structural elements are represented is the same as that in **Fig 4** of main text. Number labels for select clusters are provided. Here “sequence bias” (vertical axis) is defined as ln[*P*(GB98)/*P*(GA98)], where *P*(GA98) and *P*(GB98) are the fractions of conformations sampled, respectively, from GA98 and GB98 simulations for the given cluster. ln[*P*(GB98)/*P*(GA98)] is the population shift for a cluster after the L45Y mutation; whereas “fold bias” (horizontal axis), defined as ln(Q_B_/Q_A_), is the bias that exists within a given conformational cluster favoring (positive) or disfavoring (negative) G_B_ over G_A_. The native basins of G_A_ and G_B_ are depicted, respectively, by blue and red ovals. Centroid conformations are shown for the most GB98- and GA98-enriched clusters in the unfolded state (cluster nos. 28 and 12, respectively), as well as the most GB98- and GA98-enriched in the G_B_ intermediate state (cluster nos. 22 and 17, respectively; see text). Note that cluster no. 12 is likely a kinetic trap because its second β-hairpin is in a nonnative orientation, as discussed in conjunction with **Fig 4** of main text.
(TIFF)

S16 Fig Shift in unfolded-state contact frequencies caused by the L45Y mutation. The unfolded state is defined by Q_A_<0.6 and Q_B_<0.3. The criterion for the residue-residue contacts considered here are the same as that for native contacts (*Methods* of main text). Contact order ≡ |*i*–*j*| + 1 for a contact between residues *i* and *j*. P(A) and P(B) are the fractions, respectively, of GA98 and GB98 unfolded conformations with a given contact. The diameters of the circles representing the contacts are proportional to the overall fractional frequency [P(A)+P(B)]/2 of the contact. Circle color is used to distinguish contacts that are nonnative (black), native G_A_ (blue), native G_B_ (red), and native G_A_+G_B_ (magenta). The interacting residue pairs are identified for contacts with the largest frequency shifts. Results in this figure were obtained from an equal number of 1,000 conformations sampled from GA98 and GB98 simulations.
(TIFF)

S17 Fig Difference landscapes for alternate switches. (a) Free energy difference between GB98-T25I,L20A and GB98-T25I (former minus latter) as a function of Q_A_ and Q_B_. It is known experimentally that GB98-T25I,L20A adopts the G_B_ fold whereas GB98-T25I adopts the G_A_ fold. (b) The corresponding free energy difference between the predicted switch sequences “S1” (G_B_ fold) and “S2” (G_A_ fold). The free energy landscapes of “S1” and “S2” are given in **Fig 7c and 7d** of main text.
(TIFF)

S18 Fig Effect of our model π-π interaction for F, Y on the G_A_/G_B_ conformational switch. Shown here are the Q_A_/Q_B_ free energy landscapes of GA98 (a) and GB98 (b) in the modified hybrid model that incorporates the rudimentary π-π interaction defined in *Methods* of the main text in the model potential’s transferable component. Relative to the corresponding landscapes in the unmodified hybrid model (**Fig 3** of main text), the G_B_ basin of the GA98 landscape here (a) is much more depleted than that of the GB98 landscape (b).
(TIFF)

S1 Text Modeling details and related computational studies. (PDF)

## References

[1] Sikosek T, Chan HS. Biophysics of protein evolution and evolutionary protein biophysics. J R Soc Interface. 2014;11: 20140419 10.1098/rsif.2014.0419 25165599 PMC4191086

[2] Liberles DA, Teichmann SA, Bahar I, Bastolla U, Bloom JD, Bornberg-Bauer E, et al The interface of protein structure, protein biophysics, and molecular evolution. Protein Sci. 2012;21: 769–85. 10.1002/pro.2071 22528593 PMC3403413

[3] Harms MJ, Thornton JW. Evolutionary biochemistry: Revealing the historical and physical causes of protein properties. Nat Rev Genet. 2013;14: 559–71. 10.1038/nrg3540 23864121 PMC4418793

[4] Morcos F, Schafer NP, Cheng RR, Onuchic JN, Wolynes PG. Coevolutionary information, protein folding landscapes, and the thermodynamics of natural selection. Proc Natl Acad Sci USA. 2014;111: 12408–12413. 10.1073/pnas.1413575111 25114242 PMC4151759

[5] Tokuriki N, Tawfik DS. Protein dynamism and evolvability. Science. 2009;324: 203–7. 10.1126/science.1169375 19359577

[6] Amitai G, Gupta RD, Tawfik DS. Latent evolutionary potentials under the neutral mutational drift of an enzyme. HFSP J. 2007;1: 67–78. 10.2976/1.2739115/10.2976/1 19404461 PMC2645560

[7] Wroe R, Chan HS, Bornberg-Bauer E. A structural model of latent evolutionary potentials underlying neutral networks in proteins. HFSP J. 2007;1: 79–87. 10.2976/1.2739116/10.2976/1 19404462 PMC2645552

[8] Sikosek T, Bornberg-Bauer E, Chan HS. Evolutionary dynamics on protein bi-stability landscapes can potentially resolve adaptive conflicts. PLoS Comput Biol. 2012;8: e1002659 10.1371/journal.pcbi.1002659 23028272 PMC3441461

[9] Sato K, Ito Y, Yomo T, Kaneko K. On the relation between fluctuation and response in biological systems. Proc Natl Acad Sci USA. 2003;100: 14086–90. 14615583 10.1073/pnas.2334996100 PMC283550

[10] Bornberg-Bauer E, Chan HS. Modeling evolutionary landscapes: Mutational stability, topology, and superfunnels in sequence space. Proc Natl Acad Sci USA. 1999;96: 10689–10694. 10485887 10.1073/pnas.96.19.10689 PMC17944

[11] Sikosek T, Chan HS, Bornberg-Bauer E. Escape from Adaptive Conflict follows from weak functional trade-offs and mutational robustness. Proc Natl Acad Sci USA. 2012;109: 14888–93. 10.1073/pnas.1115620109 22927372 PMC3443171

[12] Cao B, Elber R. Computational exploration of the network of sequence flow between protein structures. Proteins. 2010;78: 985–1003. 10.1002/prot.22622 19899165 PMC2811751

[13] Holzgräfe C, Irbäck A, Troein C. Mutation-induced fold switching among lattice proteins. J Chem Phys. 2011;135: 195101 10.1063/1.3660691 22112098

[14] Holzgräfe C, Wallin S. Smooth functional transition along a mutational pathway with an abrupt protein fold switch. Biophys J. 2014;107: 1217–1225. 10.1016/j.bpj.2014.07.020 25185557 PMC4156676

[15] Ugalde JA, Chang BSW, Matz MV. Evolution of coral pigments recreated. Science. 2004;305: 1433 15353795 10.1126/science.1099597

[16] Bouvignies G, Vallurupalli P, Hansen DF, Correia BE, Lange O, Bah A, et al Solution structure of a minor and transiently formed state of a T4 lysozyme mutant. Nature. 2011;477: 111–4. 10.1038/nature10349 21857680 PMC3706084

[17] Cordes MHJ, Burton RE, Walsh NP, McKnight CJ, Sauer RT. An evolutionary bridge to a new protein fold. Nat Struct Biol. 2000;7: 1129–32. 11101895 10.1038/81985

[18] Meier S, Jensen PR, David CN, Chapman J, Holstein TW, Grzesiek S, et al Continuous molecular evolution of protein-domain structures by single amino acid changes. Curr Biol. 2007;17: 173–8. 17240343 10.1016/j.cub.2006.10.063

[19] Alexander PA, He Y, Chen Y, Orban J, Bryan PN. A minimal sequence code for switching protein structure and function. Proc Natl Acad Sci USA. 2009;106: 21149–54. 10.1073/pnas.0906408106 19923431 PMC2779201

[20] Alexander PA, He Y, Chen Y, Orban J, Bryan PN. The design and characterization of two proteins with 88% sequence identity but different structure and function. Proc Natl Acad Sci USA. 2007;104: 11963–8. 17609385 10.1073/pnas.0700922104 PMC1906725

[21] Morrone A, McCully ME, Bryan PN, Brunori M, Daggett V, Gianni S, et al The denatured state dictates the topology of two proteins with almost identical sequence but different native structure and function. J Biol Chem. 2011;286: 3863–72. 10.1074/jbc.M110.155911 21118804 PMC3030387

[22] He Y, Chen Y, Alexander PA, Bryan PN, Orban J. Mutational tipping points for switching protein folds and functions. Structure. 2012;20: 283–91. 10.1016/j.str.2011.11.018 22325777 PMC3278708

[23] Piana S, Klepeis JL, Shaw DE. Assessing the accuracy of physical models used in protein-folding simulations: Quantitative evidence from long molecular dynamics simulations. Curr Opin Struct Biol. 2014;24: 98–105. 10.1016/j.sbi.2013.12.006 24463371

[24] Skinner JJ, Yu W, Gichana EK, Baxa MC, Hinshaw JR, Freed KF, et al Benchmarking all-atom simulations using hydrogen exchange. Proc Natl Acad Sci USA. 2014;111: 15975–15980. 10.1073/pnas.1404213111 25349413 PMC4234613

[25] Allison JR, Bergeler M, Hansen N, van Gunsteren WF. Current computer modeling cannot explain why two highly similar sequences fold into different structures. Biochemistry. 2011;50: 10965–73. 10.1021/bi2015663 22082195

[26] Hansen N, Allison JR, Hodel FH, van Gunsteren WF. Relative free enthalpies for point mutations in two proteins with highly similar sequences but different folds. Biochemistry. 2013;52: 4962–70. 10.1021/bi400272q 23802564

[27] Chen S-H, Elber R. The energy landscape of a protein switch. Phys Chem Chem Phys. 2014;16: 6407–6421. 10.1039/c3cp55209h 24473276

[28] Roy A, Perez A, Dill KA, MacCallum JL. Computing the relative stabilities and the per-residue components in protein conformational changes. Structure. 2014;22: 168–75. 10.1016/j.str.2013.10.015 24316402 PMC3905753

[29] Chan HS, Zhang Z, Wallin S, Liu Z. Cooperativity, local-nonlocal coupling, and nonnative interactions: principles of protein folding from coarse-grained models. Annu Rev Phys Chem. 2011;62: 301–326. 10.1146/annurev-physchem-032210-103405 21453060

[30] Clementi C, Nymeyer H, Onuchic JN. Topological and energetic factors: what determines the structural details of the transition state ensemble and “en-route” intermediates for protein folding? An investigation for small globular proteins. J Mol Biol. 2000;298: 937–53. 10801360 10.1006/jmbi.2000.3693

[31] Hills RD, Brooks CL. Insights from coarse-grained Gō models for protein folding and dynamics. Int J Mol Sci. 2009;10: 889–905. 10.3390/ijms10030889 19399227 PMC2672008

[32] Whitford PC, Noel JK, Gosavi S, Schug A, Sanbonmatsu KY, Onuchic JN. An all-atom structure-based potential for proteins: bridging minimal models with all-atom empirical forcefields. Proteins. 2009;75: 430–41. 10.1002/prot.22253 18837035 PMC3439813

[33] Best RB, Chen Y-G, Hummer G. Slow protein conformational dynamics from multiple experimental structures: the helix/sheet transition of arc repressor. Structure. 2005;13: 1755–63. 16338404 10.1016/j.str.2005.08.009

[34] Whitford PC, Miyashita O, Levy Y, Onuchic JN. Conformational transitions of adenylate kinase: Switching by cracking. J Mol Biol. 2007;366: 1661–71. 17217965 10.1016/j.jmb.2006.11.085 PMC2561047

[35] Knott M, Best RB. Discriminating binding mechanisms of an intrinsically disordered protein via a multi-state coarse-grained model. J Chem Phys. 2014;140: 175102 10.1063/1.4873710 24811666 PMC4032430

[36] Camilloni C, Sutto L. Lymphotactin: how a protein can adopt two folds. J Chem Phys. 2009;131: 245105 10.1063/1.3276284 20059117

[37] Miyashita O, Onuchic JN, Wolynes PG. Nonlinear elasticity, proteinquakes, and the energy landscapes of functional transitions in proteins. Proc Natl Acad Sci USA. 2003;100: 12570–12575. 14566052 10.1073/pnas.2135471100 PMC240658

[38] Sutto L, Camilloni C. From A to B: a ride in the free energy surfaces of protein G domains suggests how new folds arise. J Chem Phys. 2012;136: 185101 10.1063/1.4712029 22583310

[39] Kouza M, Hansmann UHE. Folding simulations of the A and B domains of protein G. J Phys Chem B. 2012;116: 6645–53. 10.1021/jp210497h 22214186 PMC3337360

[40] Jahn TR, Parker MJ, Homans SW, Radford SE. Amyloid formation under physiological conditions proceeds via a native-like folding intermediate. Nat Struct Mol Biol. 2006;13: 195–201. 16491092 10.1038/nsmb1058 PMC7617781

[41] Krobath H, Estácio SG, Faísca PFN, Shakhnovich EI. Identification of a conserved aggregation-prone intermediate state in the folding pathways of Spc-SH3 amyloidogenic variants. J Mol Biol. 2012;422: 705–22. 10.1016/j.jmb.2012.06.020 22727745

[42] Estácio SG, Krobath H, Vila-Viçosa D, Machuqueiro M, Shakhnovich EI, Faísca PFN. A Simulated intermediate state for folding and aggregation provides insights into ΔN6 β2-microglobulin amyloidogenic behavior. PLoS Comput Biol. 2014;10: e1003606 10.1371/journal.pcbi.1003606 24809460 PMC4014404

[43] Zarrine-Afsar A, Wallin S, Neculai AM, Neudecker P, Howell PL, Davidson AR, et al Theoretical and experimental demonstration of the importance of specific nonnative interactions in protein folding. Proc Natl Acad Sci USA. 2008;105: 9999–10004. 10.1073/pnas.0801874105 18626019 PMC2481363

[44] Azia A, Levy Y. Nonnative electrostatic interactions can modulate protein folding: Molecular dynamics with a grain of salt. J Mol Biol. 2009;393: 527–542. 10.1016/j.jmb.2009.08.010 19683007

[45] Zhang Z, Chan HS. Competition between native topology and nonnative interactions in simple and complex folding kinetics of natural and designed proteins. Proc Natl Acad Sci USA. 2010;107: 2920–2925. 10.1073/pnas.0911844107 20133730 PMC2840274

[46] Sutto L, Mereu I, Gervasio FL. A hybrid all-atom structure-based model for protein folding and large scale conformational transitions. J Chem Theory Comput. 2011;7: 4208–4217. 10.1021/ct200547m 26598361

[47] Wang Y, Chu X, Longhi S, Roche P, Han W, Wang E, et al Multiscaled exploration of coupled folding and binding of an intrinsically disordered molecular recognition element in measles virus nucleoprotein. Proc Natl Acad Sci USA. 2013;110: E3743–E3752. 10.1073/pnas.1308381110 24043820 PMC3791790

[48] Yadahalli S, Hemanth Giri Rao V, Gosavi S. Modeling non-native interactions in designed proteins. Isr J Chem. 2014;54: 1230–1240. 10.1002/ijch.201400035

[49] Chen T, Song J, Chan HS. Theoretical perspectives on nonnative interactions and intrinsic disorder in protein folding and binding. Curr Opin Struct Biol. 2015;30: 32–42. 10.1016/j.sbi.2014.12.002 25544254

[50] Chen T, Chan HS. Native contact density and nonnative hydrophobic effects in the folding of bacterial immunity proteins. PLOS Comput Biol. 2015;11: e1004260 10.1371/journal.pcbi.1004260 26016652 PMC4446218

[51] Ramírez-Sarmiento CA, Noel JK, Valenzuela SL, Artsimovitch I. Interdomain contacts control native state switching of RfaH on a dual-funneled landscape. PLOS Comput Biol. 2015;11: e1004379 10.1371/journal.pcbi.1004379 26230837 PMC4521827

[52] Meyer EA, Castellano RK, Diederich F. Interactions with aromatic rings in chemical and biological recognition. Angew Chemie Int Ed. 2003;42: 1210–1250. 10.1002/anie.200390319 12645054

[53] Lammert H, Schug A, Onuchic JN. Robustness and generalization of structure-based models for protein folding and function. Proteins. 2009;77: 881–91. 10.1002/prot.22511 19626713

[54] Chavez LL, Onuchic JN, Clementi C. Quantifying the roughness on the free energy landscape: Entropic bottlenecks and protein folding rates. J Am Chem Soc. 2004;126: 8426–32. 15237999 10.1021/ja049510+

[55] Irbäck A, Mitternacht S, Mohanty S. An effective all-atom potential for proteins. BMC Biophys. 2009;2: 2 10.1186/1757-5036-2-2 PMC2696411 19356242

[56] Irbäck A, Mohanty S. PROFASI: A Monte Carlo simulation package for protein folding and aggregation. J Comput Chem. 2006;27: 1548–55. 16847934 10.1002/jcc.20452

[57] Sali A, Shakhnovich EI, Karplus M. Kinetics of protein folding. A lattice model study of the requirements for folding to the native state. J Mol Biol. 1994;235: 1614–36. 8107095 10.1006/jmbi.1994.1110

[58] Cho SS, Levy Y, Wolynes PG. P versus Q: Structural reaction coordinates capture protein folding on smooth landscapes. Proc Natl Acad Sci USA. 2006;103: 586–591. 16407126 10.1073/pnas.0509768103 PMC1334664

[59] Cheung MS, García AE, Onuchic JN. Protein folding mediated by solvation: water expulsion and formation of the hydrophobic core occur after the structural collapse. Proc Natl Acad Sci USA. 2002;99: 685–90. 11805324 10.1073/pnas.022387699 PMC117366

[60] Chen T, Chan HS. Effects of desolvation barriers and sidechains on local–nonlocal coupling and chevron behaviors in coarse-grained models of protein folding. Phys Chem Chem Phys. 2014;16: 6460–6479. 10.1039/c3cp54866j 24554086

[61] MacCallum JL, Moghaddam MS, Chan HS, Tieleman DP. Hydrophobic association of alpha-helices, steric dewetting, and enthalpic barriers to protein folding. Proc Natl Acad Sci USA. 2007;104: 6206–10. 17404236 10.1073/pnas.0605859104 PMC1847460

[62] Dias CL, Chan HS. Pressure-Dependent Properties of Elementary Hydrophobic Interactions: Ramifications for Activation Properties of Protein Folding. J Phys Chem B. 2014;118: 7488–7509. 10.1021/jp501935f 24933471

[63] Guo Z, Brooks CL, Boczko EM. Exploring the folding free energy surface of a three-helix bundle protein. Proc Natl Acad Sci USA. 1997;94: 10161–10166. 9294180 10.1073/pnas.94.19.10161 PMC23332

[64] García AE, Onuchic JN. Folding a protein in a computer: an atomic description of the folding/unfolding of protein A. Proc Natl Acad Sci USA. 2003;100: 13898–13903. 14623983 10.1073/pnas.2335541100 PMC283518

[65] Sutto L, Latzer J, Hegler JA, Ferreiro DU, Wolynes PG. Consequences of localized frustration for the folding mechanism of the IM7 protein. Proc Natl Acad Sci USA. 2007;104: 19825–19830. 18077415 10.1073/pnas.0709922104 PMC2148258

[66] Chen S-H, Meller J, Elber R. Comprehensive analysis of sequences of a protein switch. Protein Sci. 2016;25: 135–146. 10.1002/pro.2723 26073558 PMC4815306

[67] Salonen LM, Ellermann M, Diederich F. Aromatic rings in chemical and biological recognition: Energetics and structures. Angew Chemie—Int Ed. 2011;50: 4808–4842. 10.1002/anie.201007560 21538733

[68] Marsili S, Chelli R, Schettino V, Procacci P. Thermodynamics of stacking interactions in proteins. Phys Chem Chem Phys. 2008;10: 2673–2685. 10.1039/b718519g 18464982

[69] Brown S, Head-Gordon T. Intermediates and the folding of proteins L and G. Protein Sci. 2004;13: 958–70. 15044729 10.1110/ps.03316004 PMC2280051

[70] Fawzi NL, Chubukov V, Clark LA, Brown S, Head-Gordon T. Influence of denatured and intermediate states of folding on protein aggregation. Protein Sci. 2005;14: 993–1003. 15772307 10.1110/ps.041177505 PMC2253448

[71] Kmiecik S, Kolinski A. Folding pathway of the B1 domain of protein G explored by multiscale modeling. Biophys J. 2008;94: 726–36. 17890394 10.1529/biophysj.107.116095 PMC2186257

[72] Hubner IA, Shimada J, Shakhnovich EI. Commitment and nucleation in the protein G transition state. J Mol Biol. 2004;336: 745–761. 15095985 10.1016/j.jmb.2003.12.032

[73] Karanicolas J, Brooks CL. The origins of asymmetry in the folding transition states of protein L and protein G. Protein Sci. 2002;11: 2351–2361. 12237457 10.1110/ps.0205402 PMC2373711

[74] McCallister EL, Alm E, Baker D. Critical role of beta-hairpin formation in protein G folding. Nat Struct Biol. 2000;7: 669–673. 10932252 10.1038/77971

[75] Alexander P, Fahnestock S, Lee T, Orban J, Bryan P. Thermodynamic analysis of the folding of the streptococcal protein G IgG-binding domains B1 and B2: Why small proteins tend to have high denaturation temperatures. Biochemistry. 1992;31: 3597–3603. 1567818 10.1021/bi00129a007

[76] Park SH, Shastry MC, Roder H. Folding dynamics of the B1 domain of protein G explored by ultrarapid mixing. Nat Struct Biol. 1999;6: 943–7. 10504729 10.1038/13311

[77] Krantz BA, Mayne L, Rumbley J, Englander SW, Sosnick TR. Fast and slow intermediate accumulation and the initial barrier mechanism in protein folding. J Mol Biol. 2002;324: 359–371. 12441113 10.1016/s0022-2836(02)01029-x

[78] Chung HS, Louis JM, Eaton WA. Experimental determination of upper bound for transition path times in protein folding from single-molecule photon-by-photon trajectories. Proc Natl Acad Sci USA. 2009;106: 11837–44. 10.1073/pnas.0901178106 19584244 PMC2715475

[79] Morrone A, Giri R, Toofanny RD, Travaglini-Allocatelli C, Brunori M, Daggett V, et al GB1 is not a two-state folder: Identification and characterization of an on-pathway intermediate. Biophys J. 2011;101: 2053–2060. 10.1016/j.bpj.2011.09.013 22004760 PMC3192981

[80] Lapidus LJ, Acharya S, Schwantes CR, Wu L, Shukla D, King M, et al Complex pathways in folding of protein G explored by simulation and experiment. Biophys J. 2014;107: 947–955. 10.1016/j.bpj.2014.06.037 25140430 PMC4147789

[81] Giri R, Morrone A, Travaglini-Allocatelli C, Jemth P, Brunori M, Gianni S. Folding pathways of proteins with increasing degree of sequence identities but different structure and function. Proc Natl Acad Sci USA. 2012;109: 17772–6. 10.1073/pnas.1201794109 22652570 PMC3497760

[82] Blanco FJ, Rivas G, Serrano L. A short linear peptide that folds into a native stable beta-hairpin in aqueous solution. Nat Struct Biol. 1994;1: 584–590. 7634098 10.1038/nsb0994-584

[83] Blanco FJ, Jiménez MA, Pineda A, Rico M, Santoro J, Nieto JL. NMR solution structure of the isolated N-terminal fragment of protein-G B1 domain. Evidence of trifluoroethanol induced native-like beta-hairpin formation. Biochemistry. 1994;33: 6004–6014. 8180228 10.1021/bi00185a041

[84] Kuszewski J, Clore GM, Gronenborn AM. Fast folding of a prototypic polypeptide: the immunoglobulin binding domain of streptococcal protein G. Protein Sci. 1994;3: 1945–1952. 7703841 10.1002/pro.5560031106 PMC2142643

[85] Fersht AR, Sato S. Phi-value analysis and the nature of protein-folding transition states. Proc Natl Acad Sci USA. 2004;101: 7976–7981. 15150406 10.1073/pnas.0402684101 PMC419542

[86] Mohanty S, Meinke JH, Zimmermann O, Hansmann UHE. Simulation of Top7-CFr: A transient helix extension guides folding. Proc Natl Acad Sci USA. 2008;105: 8004–7. 10.1073/pnas.0708411105 18408166 PMC2786944

[87] Mohanty S, Meinke JH, Zimmermann O. Folding of Top7 in unbiased all-atom Monte Carlo simulations. Proteins. 2013;81: 1446–56. 10.1002/prot.24295 23553942

[88] Piana S, Lindorff-Larsen K, Shaw DE. Atomic-level description of ubiquitin folding. Proc Natl Acad Sci USA. 2013;110: 5915–5920. 10.1073/pnas.1218321110 23503848 PMC3625349

[89] Lindorff-Larsen K, Piana S, Dror RO, Shaw DE. How Fast-Folding Proteins Fold. Science. 2011;334: 517–520. 10.1126/science.1208351 22034434

[90] Bowman GR, Voelz VA, Pande VS. Taming the complexity of protein folding. Curr Opin Struct Biol. 2011;21: 4–11. 10.1016/j.sbi.2010.10.006 21081274 PMC3042729

[91] Piana S, Donchev AG, Robustelli P, Shaw DE. Water dispersion interactions strongly influence simulated structural properties of disordered protein states. J Phys Chem B. 2015;119: 5113–5123. 10.1021/jp508971m 25764013

[92] Kundrotas PJ, Zhu Z, Janin J, Vakser IA. Templates are available to model nearly all complexes of structurally characterized proteins. Proc Natl Acad Sci USA. 2012;109: 9438–41. 10.1073/pnas.1200678109 22645367 PMC3386081

[93] Kamisetty H, Ovchinnikov S, Baker D. Assessing the utility of coevolution-based residue-residue contact predictions in a sequence- and structure-rich era. Proc Natl Acad Sci USA. 2013;110: 15674–9. 10.1073/pnas.1314045110 24009338 PMC3785744

[94] Potapov V, Cohen M, Schreiber G. Assessing computational methods for predicting protein stability upon mutation: good on average but not in the details. Protein Eng Des Sel. 2009;22: 553–60. 10.1093/protein/gzp030 19561092

[95] Jiang P, Hansmann UHE. Modeling structural flexibility of proteins with Go-models. J Chem Theory Comput. 2012;8: 2127–2133. 24039551 10.1021/ct3000469 PMC3771579

[96] Metropolis N, Rosenbluth AW, Rosenbluth MN, Teller AH, Teller E. Equation of state calculations by fast computing machines. J Chem Phys. 1953;21: 1087–1092. 10.1063/1.1699114

[97] Sugita Y, Okamoto Y. Replica-exchange molecular dynamics method for protein folding. Chem Phys Lett. 1999;314: 141–151. 10.1016/S0009-2614(99)01123-9

[98] Favrin G, Irbäck A, Sjunnesson F. Monte Carlo update for chain molecules: Biased Gaussian steps in torsional space. J Chem Phys. 2001;114: 8154–8158. 10.1063/1.1364637

[99] Kumar S, Rosenberg JM, Bouzida D, Swendsen RH, Kollman PA. The weighted histogram analysis method for free-energy calculations on biomolecules. I. The method. J Comput Chem. 1992;13: 1011–1021. 10.1002/jcc.540130812

[100] McGibbon RT, Beauchamp KA, Harrigan MP, Klein C, Swails JM, Hernández CX, et al MDTraj: A modern open library for the analysis of molecular dynamics trajectories. Biophys J. 2015;109: 1528–1532. 10.1016/j.bpj.2015.08.015 26488642 PMC4623899

[101] Theobald DL. Rapid calculation of RMSDs using a quaternion-based characteristic polynomial. Acta Crystallogr Sect A Found Crystallogr. 2005;61: 478–480. 10.1107/S0108767305015266 15973002

[102] MacQueen JB. Some Methods for classification and analysis of multivariate observations. Proc Symposium on Mathematical Statistics and Probability. The Regents of the University of California; 1967. pp. 281–297.

[103] Gallivan JP, Dougherty DA. Cation-pi interactions in structural biology. Proc Natl Acad Sci USA. 1999;96: 9459–64. 10449714 10.1073/pnas.96.17.9459 PMC22230

[104] Crowley PB, Golovin A. Cation-pi interactions in protein-protein interfaces. Proteins. 2005;59: 231–9. 15726638 10.1002/prot.20417

[105] Hooft RWW, Sander C, Vriend G. Verification of protein structures: Side-chain planarity. J Appl Crystallogr. 1996;29: 714–716. 10.1107/S0021889896008631

