# Supplementary figures and images for: Theoretical Insights into the Biophysics of Protein Bi-stability and Evolutionary Switches
Source: PLoS Comput Biol. 2016 Jun 2;12(6):e1004960. doi: 10.1371/journal.pcbi.1004960 (PMC4890782; doi:10.1371/journal.pcbi.1004960)

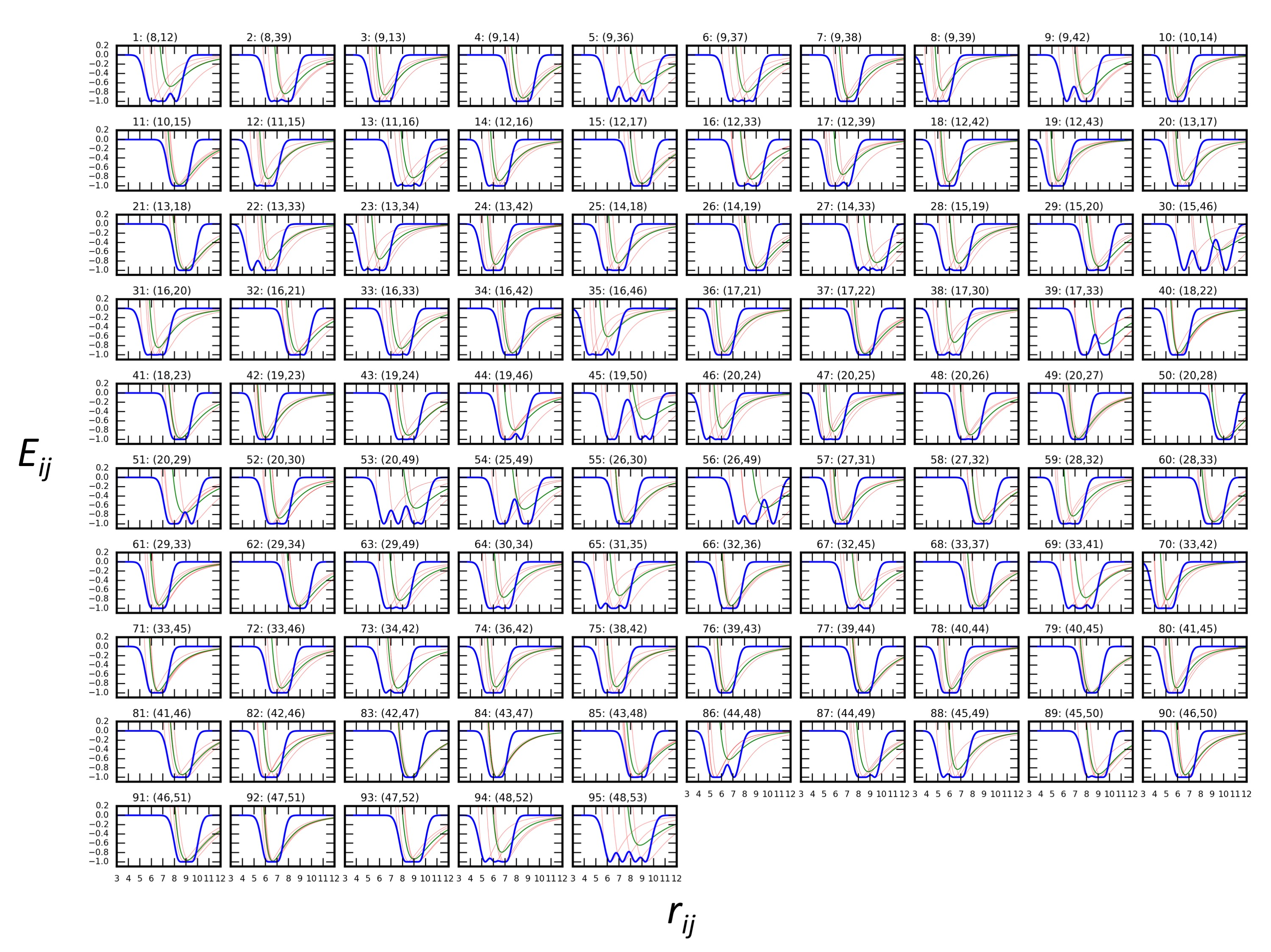

Supplement: S1 Fig — The contacts are numbered arbitrarily from 1 to 95. Residue pairs of the contacts are in parentheses. Here contact energy Eij (in units of ε) is a function of Cα-Cα distances rij (in Å). For each contact, a single Gaussian multi-well potential derived from the corresponding Cα-Cα distances d(s)ij in the PDB structures of four GA sequences is in blue. For comparison, four separate Lennard-Jones (LJ) potentials 4ε[(d(s)ij/rij)12 − (d(s)ij/rij)6] with the same native Cα-Cα distances and well depth ε are shown in red, and the linear combination of the four Lennard-Jones potentials Σs ε[(d(s)ij/rij)12 − (d(s)ij/rij)6], each with well depths scaled down to ε/4, is in green. Details of our construction of multi-well Gaussian contact potentials are given in Methods of main text. (TIFF) [file pcbi.1004960.s001.tiff]

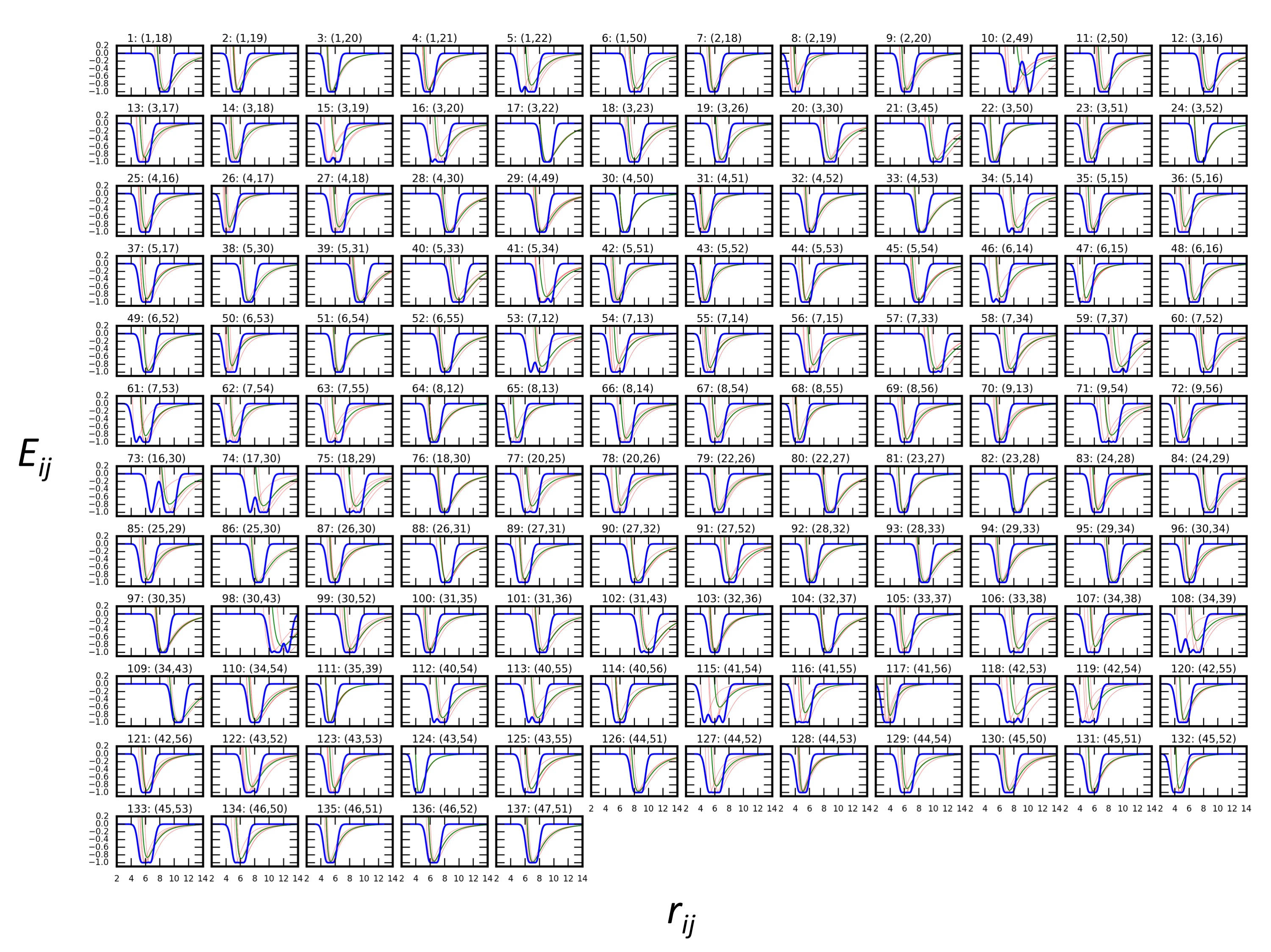

Supplement: S2 Fig — Same as S1 Fig but here the potentials were derived from the known folded structures of four GB sequences. (TIFF) [file pcbi.1004960.s002.tiff]

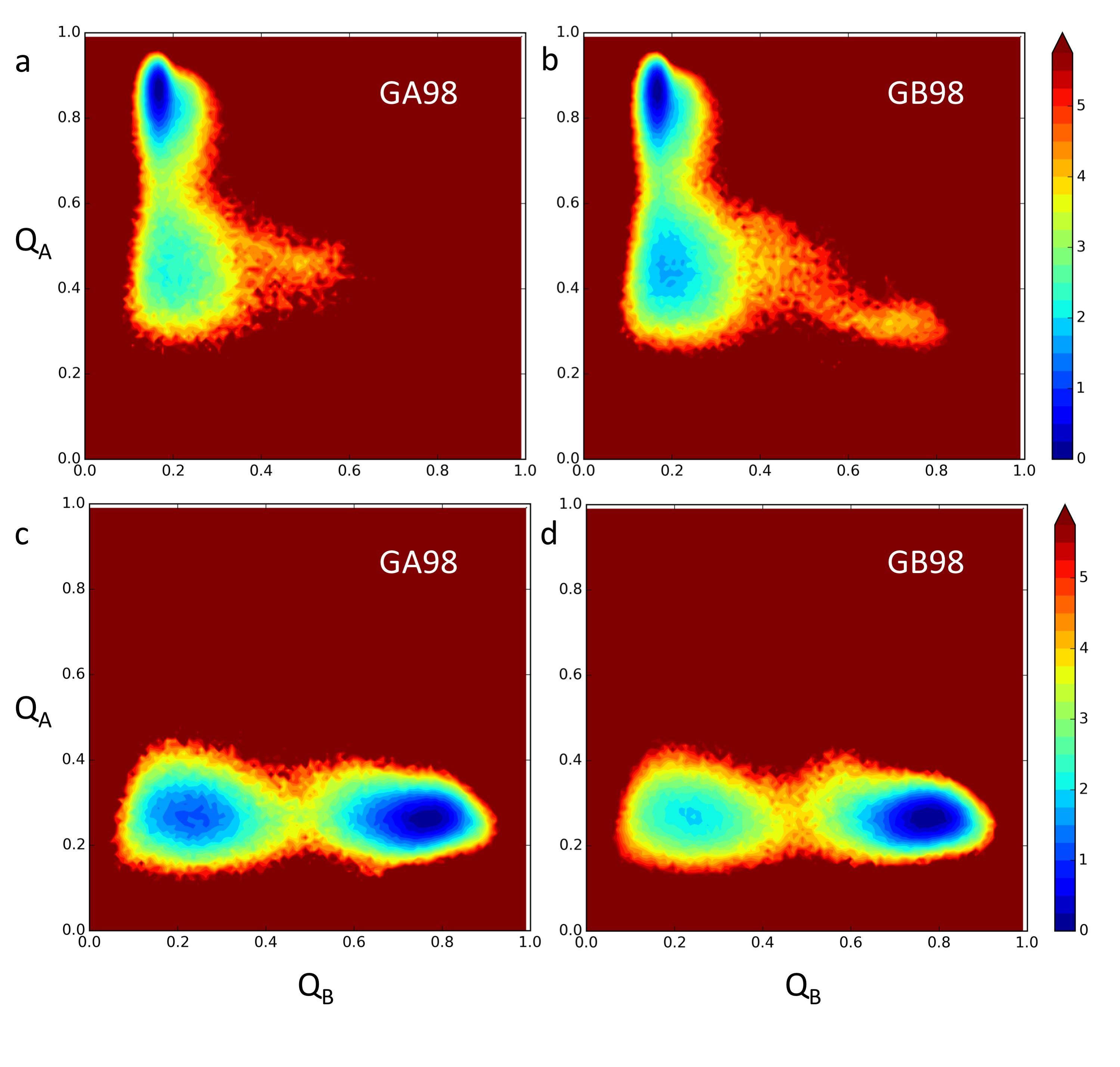

Supplement: S3 Fig — Free energy as function of QA and QB was computed using replica exchange for GA98 (left) and GB98 (right) for different ratios of SBM energies for GA and GB at the different models’ respective Tms, with εB = −1 throughout. (a,b) The GA and GB SBM basins have the same minimum energy, viz., min(EA) = min(EB). (c,d) The individual native contact strengths in GA and GB are identical, i.e., εA = εB. (TIFF) [file pcbi.1004960.s003.tiff]

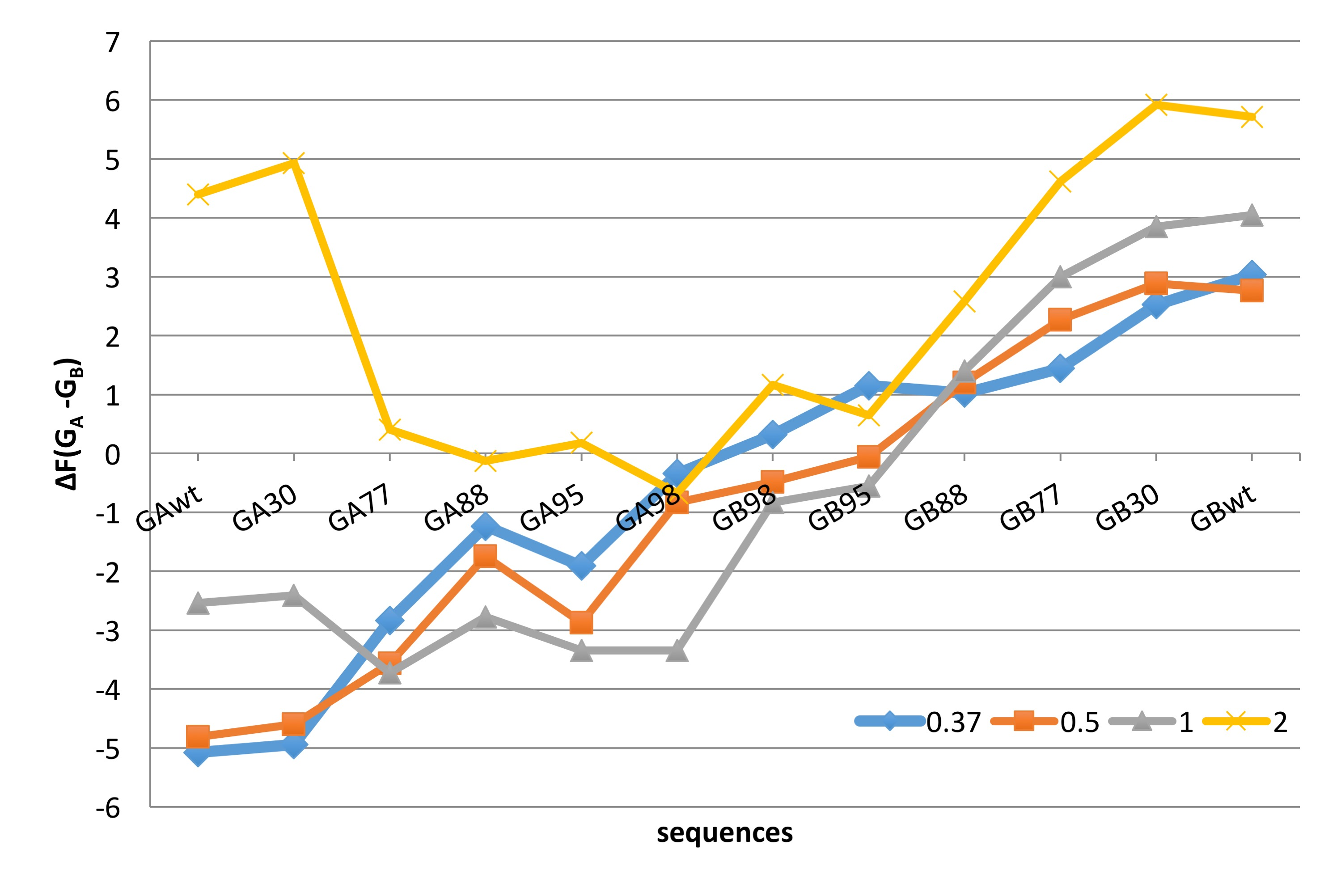

Supplement: S4 Fig — Results for the ‒εB values tested are shown in different color as indicated, with εA = 0.96εB throughout. Negative or positive value along the vertical axis indicates how much the thermodynamic equilibrium is biased, respectively, toward the GA or GB native state. (TIFF) [file pcbi.1004960.s004.tiff]

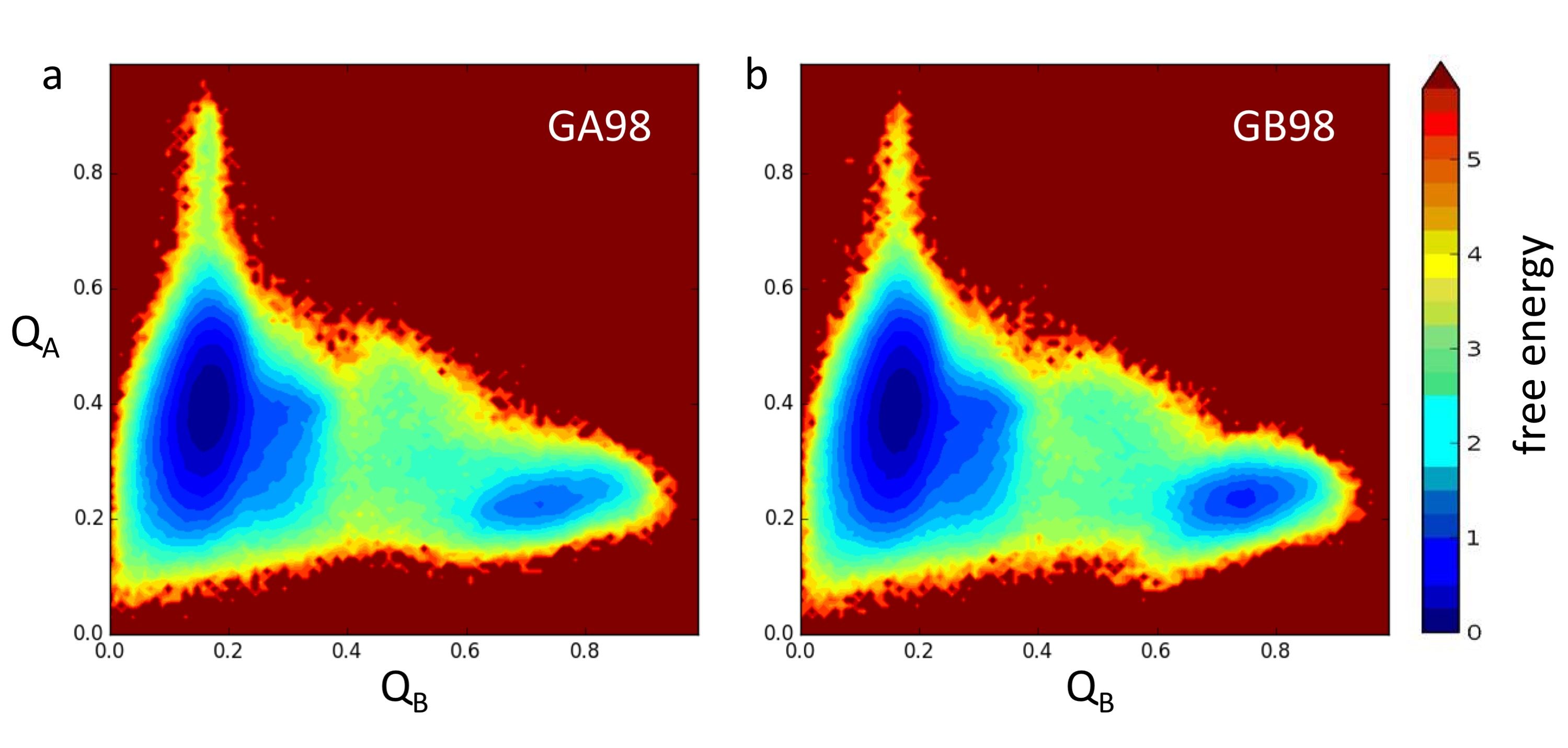

Supplement: S5 Fig — Free energy as a function of QA and QB was simulated for GA98 (a) and GB98 (b) at each sequence’ respective Tm and εB = −0.25. For each sequence, 128 independent trajectories were simulated over 107 Monte Carlo cycles. The free energy for each sequence was computed from the sampled population as a whole after discarding the first 30% of every trajectory. (TIFF) [file pcbi.1004960.s005.tiff]

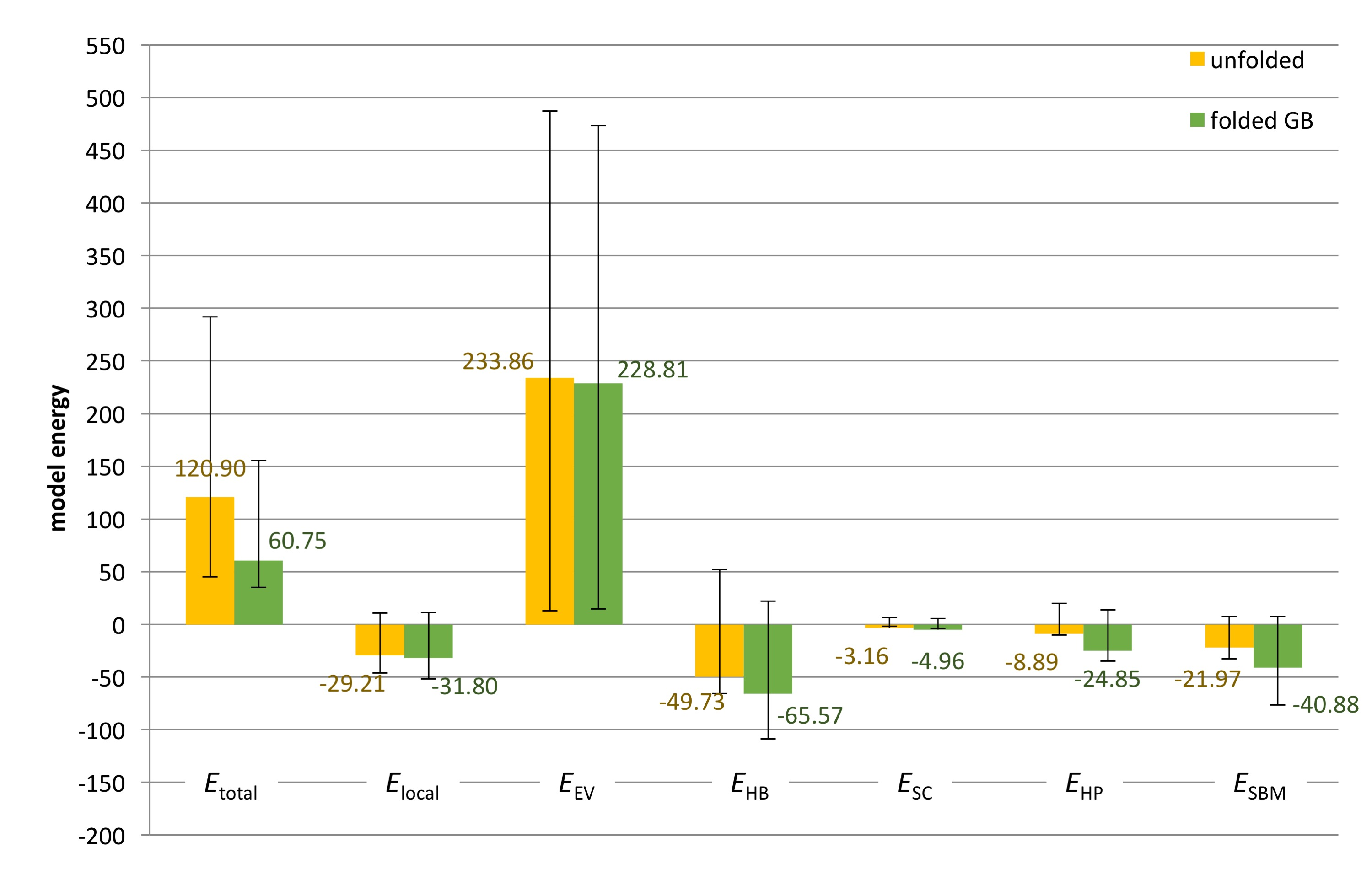

Supplement: S6 Fig — Shown here as examples are energies averaged over sampled GB98 conformations in the unfolded state (yellow columns) and in the GB-folded state (green columns) simulated using εB = −0.37. The unfolded state is defined by QA ≤ 0.6 and QB ≤ 0.3, the GB folded state is defined by QB > 0.7. The columns and numbers show the average total energy (Etotal) and its contributing averages from various energy terms, the error bars mark the ranges of energies sampled. As defined in Methods of main text, ESBM is the dual SBM term EA+EB, which is seen to have a minor stabilizing contribution (negative value) compared to the sum of transferrable terms such as the torsion-related (Elocal), hydrogen-bonding (EHB), and hydrophobic (EHP) terms. Because of a large repulsive contribution from the transferrable excluded-volume term (EEV) and an almost neutral contribution from charged side-chain interactions (ESC), the average total energy Etotal is positive. Further details are provided in Methods of main text and Irbäck et al., 2009 cited in S1 Text. (TIFF) [file pcbi.1004960.s006.tiff]

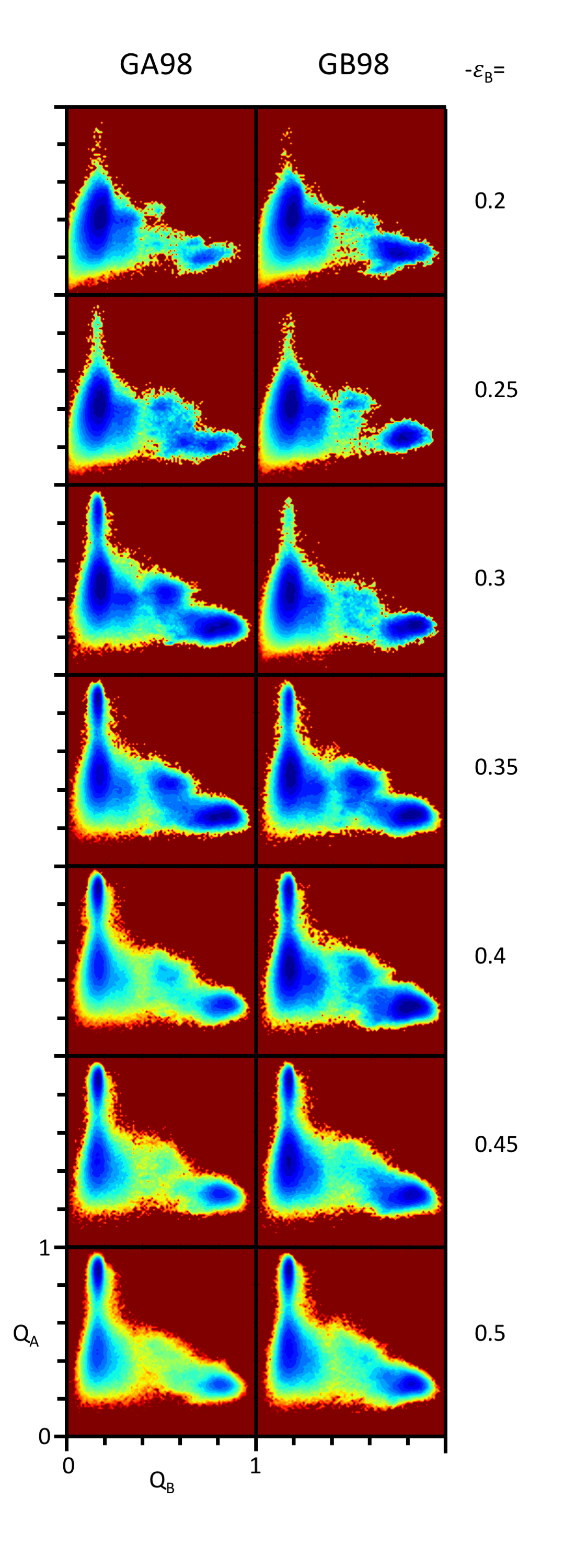

Supplement: S7 Fig — QA vs QB free energy landscapes obtained after reweighting to the respective Tm. Simulation procedure and plotting style are the same as that described for Fig 3a of main text. (TIFF) [file pcbi.1004960.s007.tiff]

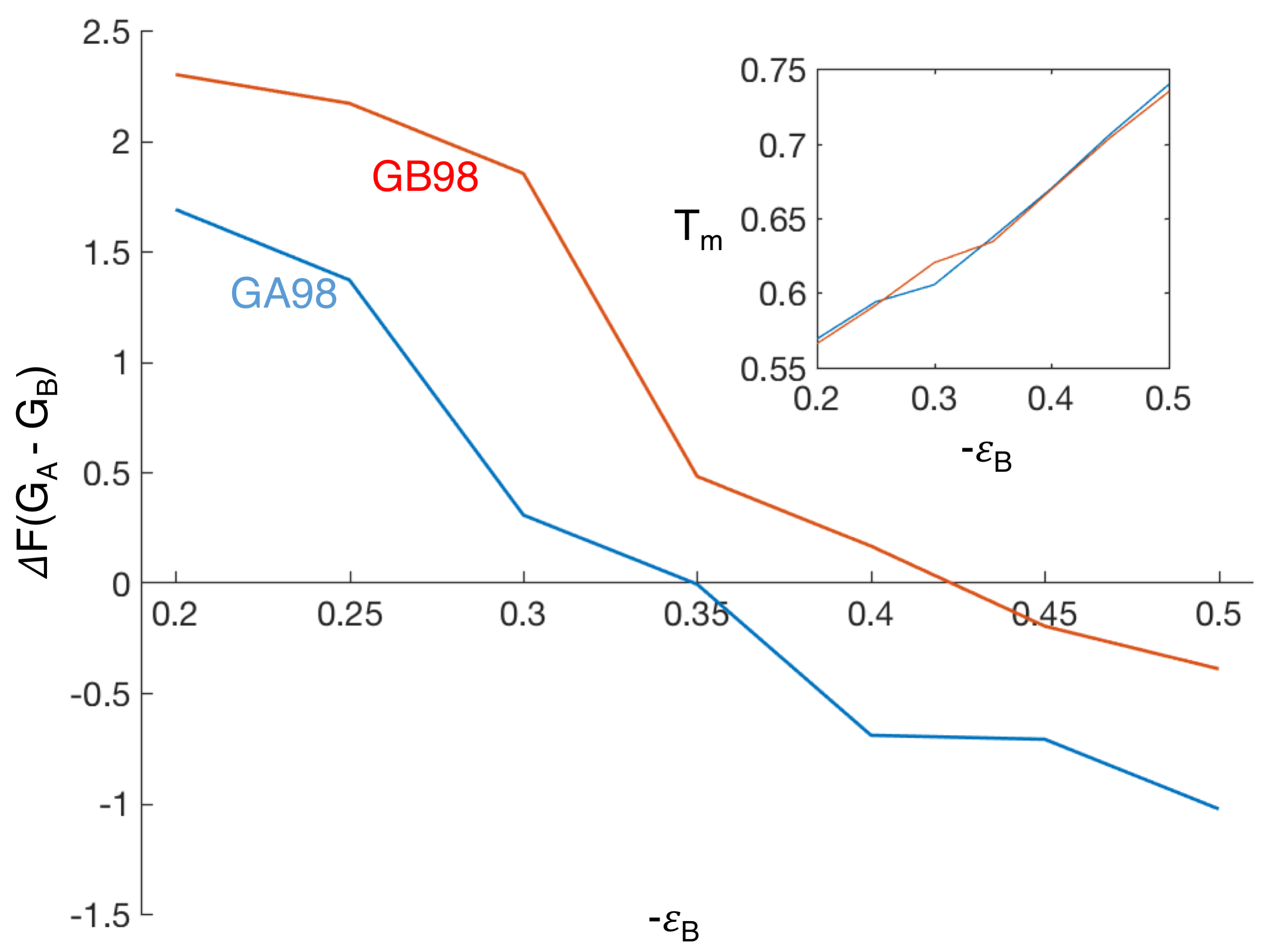

Supplement: S8 Fig — Note that the GA fold is always favored more by GA98 than by GB98, whereas the GB fold is always favored more by GB98 than by GA98. (TIFF) [file pcbi.1004960.s008.tiff]

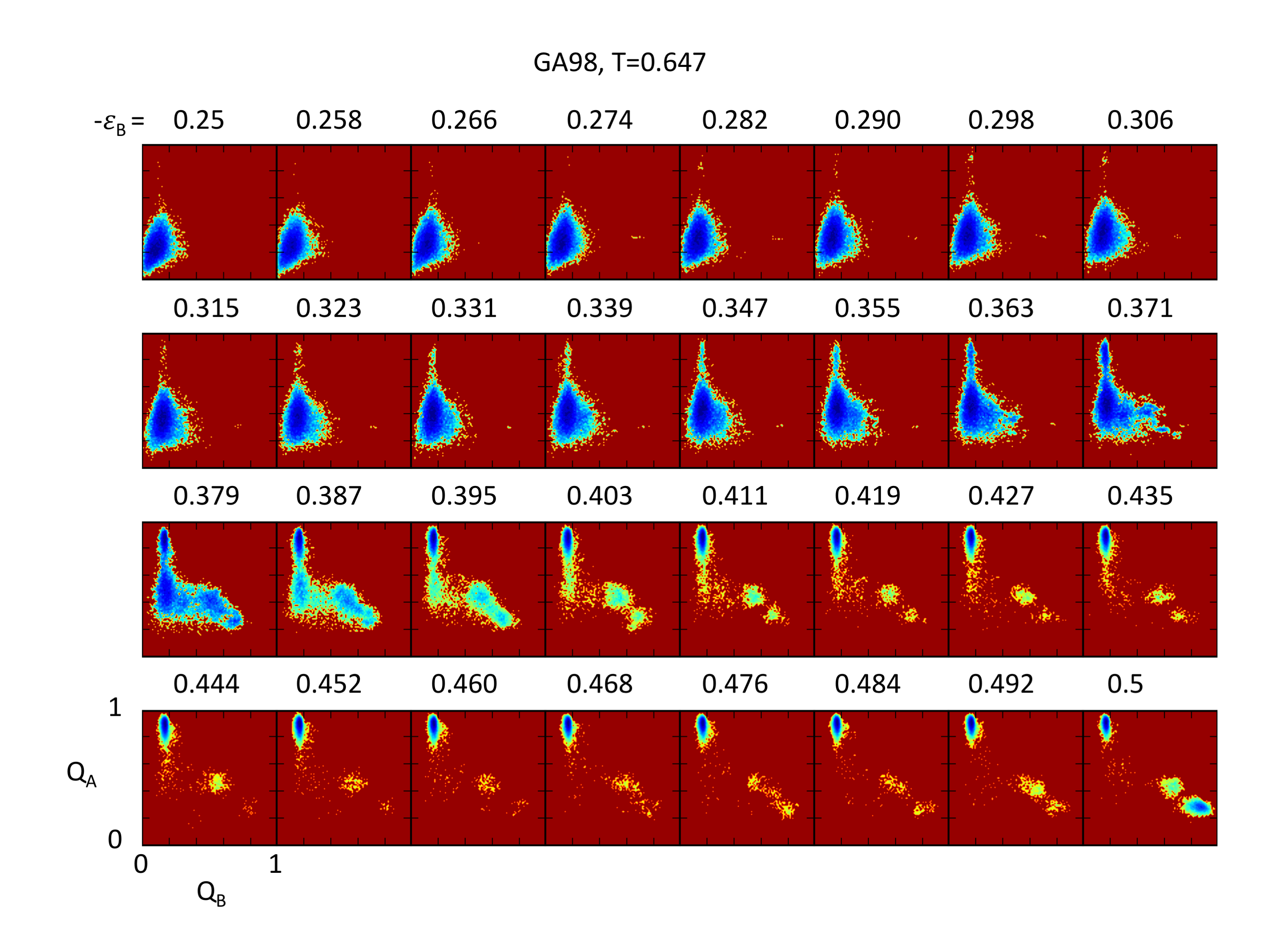

Supplement: S9 Fig — Simulations were conducted at the constant temperature shown at the top. The landscapes are depicted in the same style as that in S7 Fig. (TIFF) [file pcbi.1004960.s009.tiff]

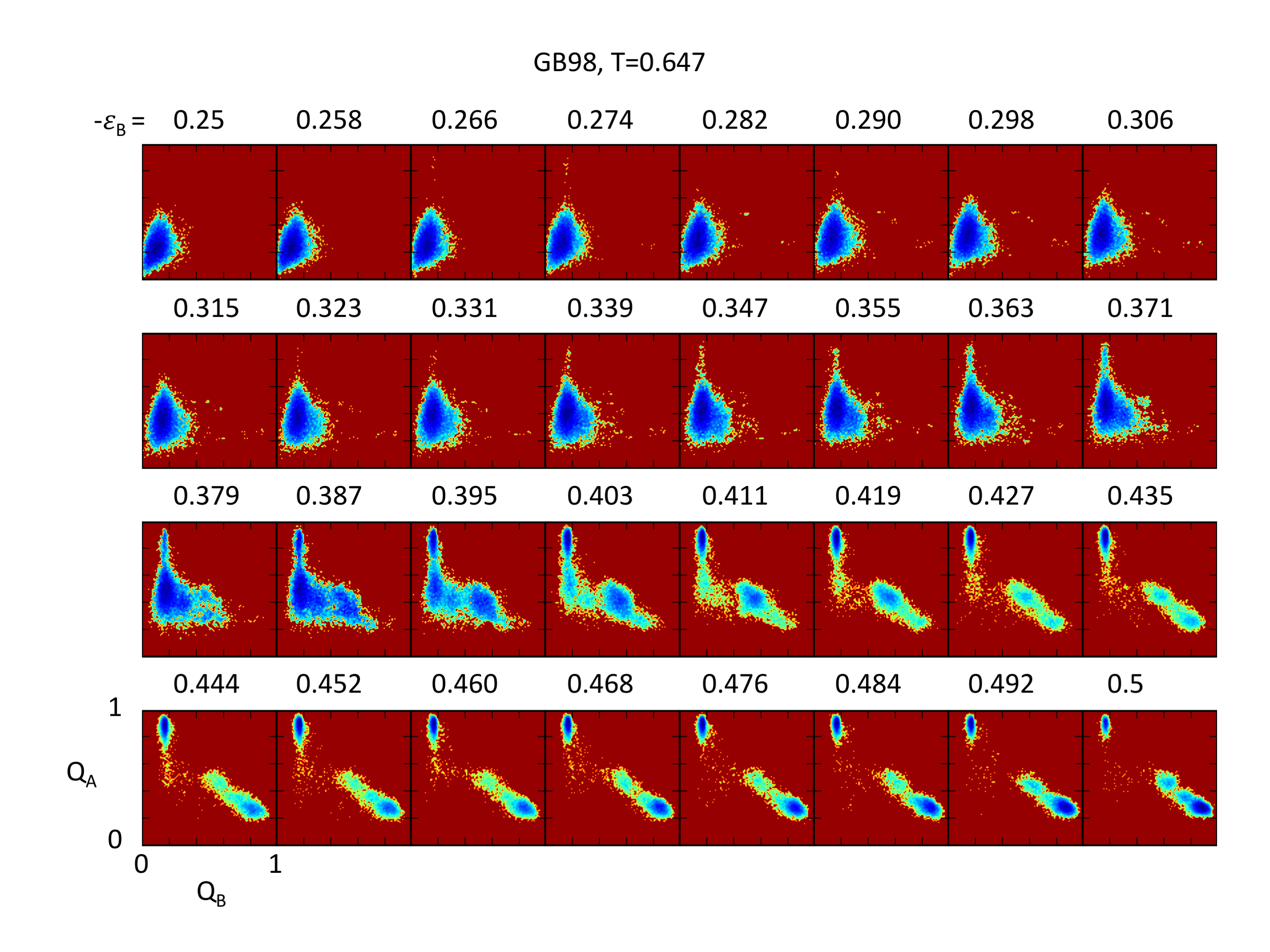

Supplement: S10 Fig — Simulations were conducted at the constant temperature shown at the top. The landscapes are depicted in the same style as that in S9 Fig. (TIFF) [file pcbi.1004960.s010.tiff]

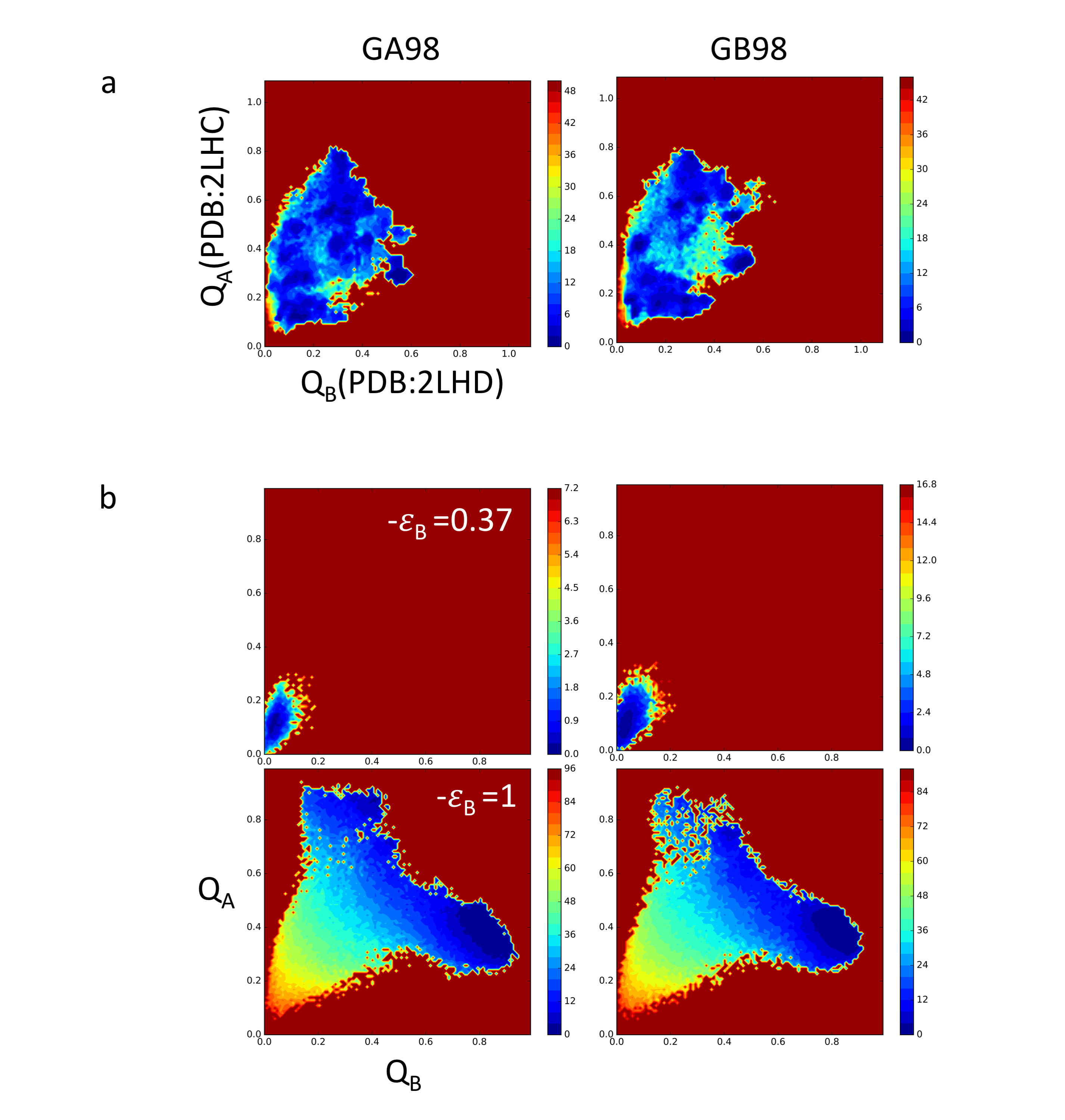

Supplement: S11 Fig — Free energy landscapes computed by simulations of GA98 and GB98 (a) using only the Lund potential without SBM, and (b) with all long-range interactions in the Lund potential turned off, but with the SBM on. In (a), the QA and QB reaction coordinates were based, respectively, on the 2LHC and 2LHD PDB structures. In (b), −εB was either 0.37 (top) or 1 (bottom). A wide temperature grid was used for temperature replica exchange to sample both folded and unfolded conformations. Free energy in each panel is plotted in units of kBT according to the scale on the right. (TIFF) [file pcbi.1004960.s011.tiff]

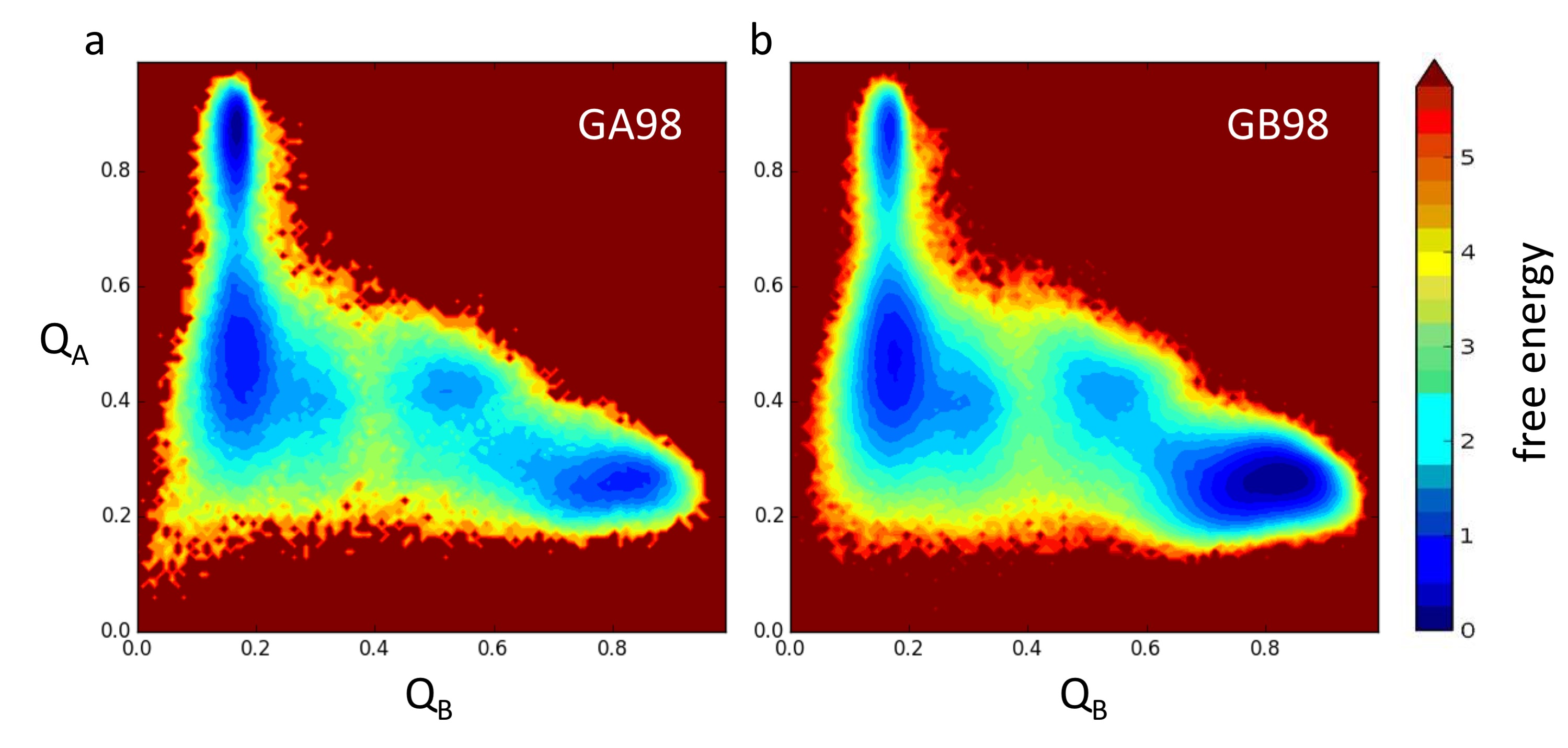

Supplement: S12 Fig — Free energy as a function of QA and QB was simulated for GA98 (a) and GB98 (b) at each sequence’s respective Tm and εB = −0.37. For each sequence, 128 independent trajectories were simulated over 107 Monte Carlo cycles. The free energy for each sequence was computed from the sampled population as a whole after discarding the first 30% of every trajectory. This calculation gives ΔF(GA-GB) = −0.8 for GA98 and ΔF(GA-GB) = +0.77 for GB98. (TIFF) [file pcbi.1004960.s012.tiff]

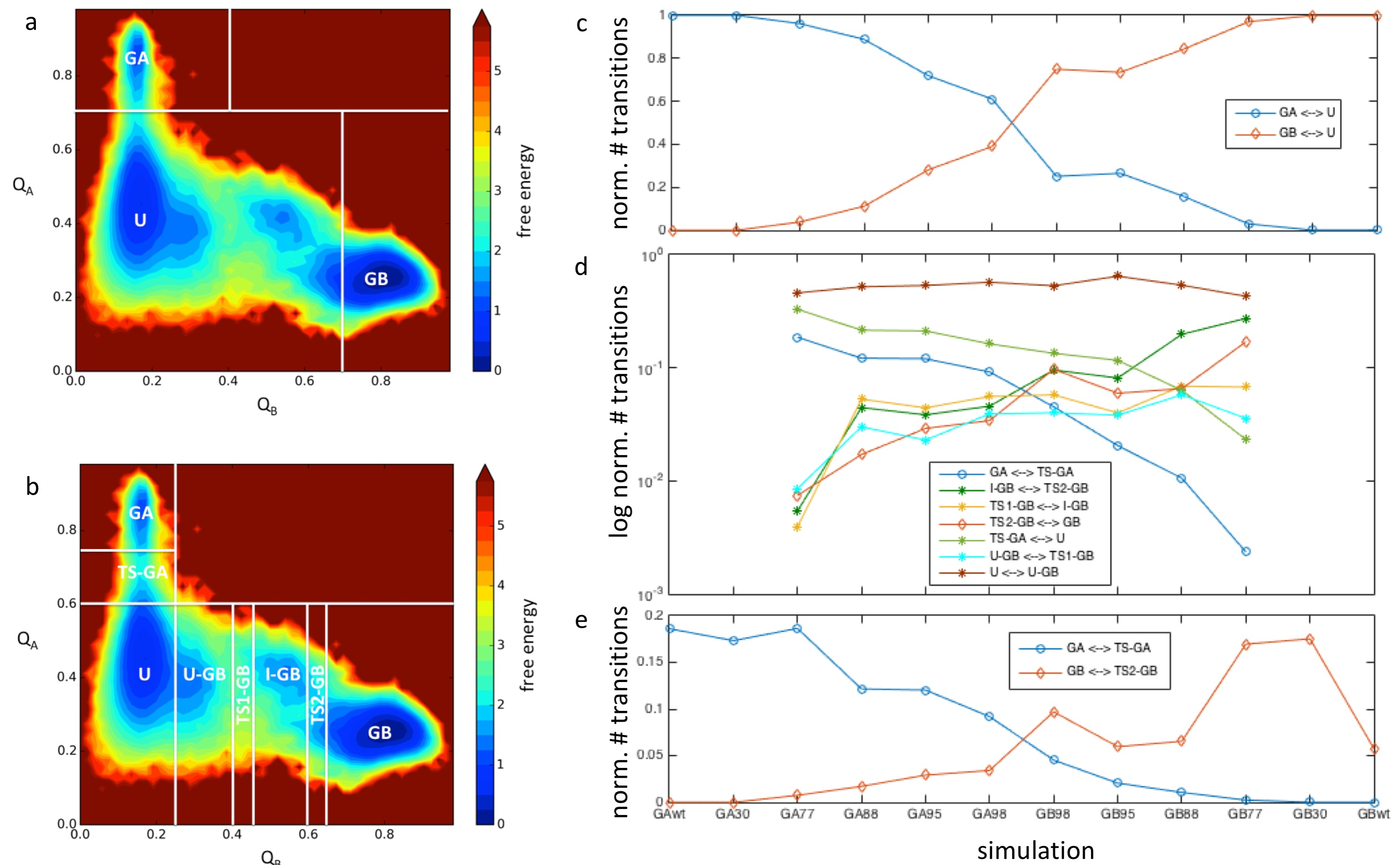

Supplement: S13 Fig — For this analysis, conformations in the QA/QB energy landscapes are divided into three (a) or eight (b) macroscopic states. Transition frequencies between these states during Monte Carlo sampling were recorded. Normalized two-way transition frequencies shown here are for (c) GA (folded) and U (unfolded), GB (folded) and U (unfolded) in the case of three macroscopic states; (d) all neighboring states, and (e) GA and its neighboring transition state as well as GB and its neighboring transition state in the case of three macroscopic states (d,e). Data are provided here for all twelve GA/GB sequence variants in (a) and (c); but only for eight variants in (b) for which the transitions of interest were observable during our simulations. (TIFF) [file pcbi.1004960.s013.tiff]

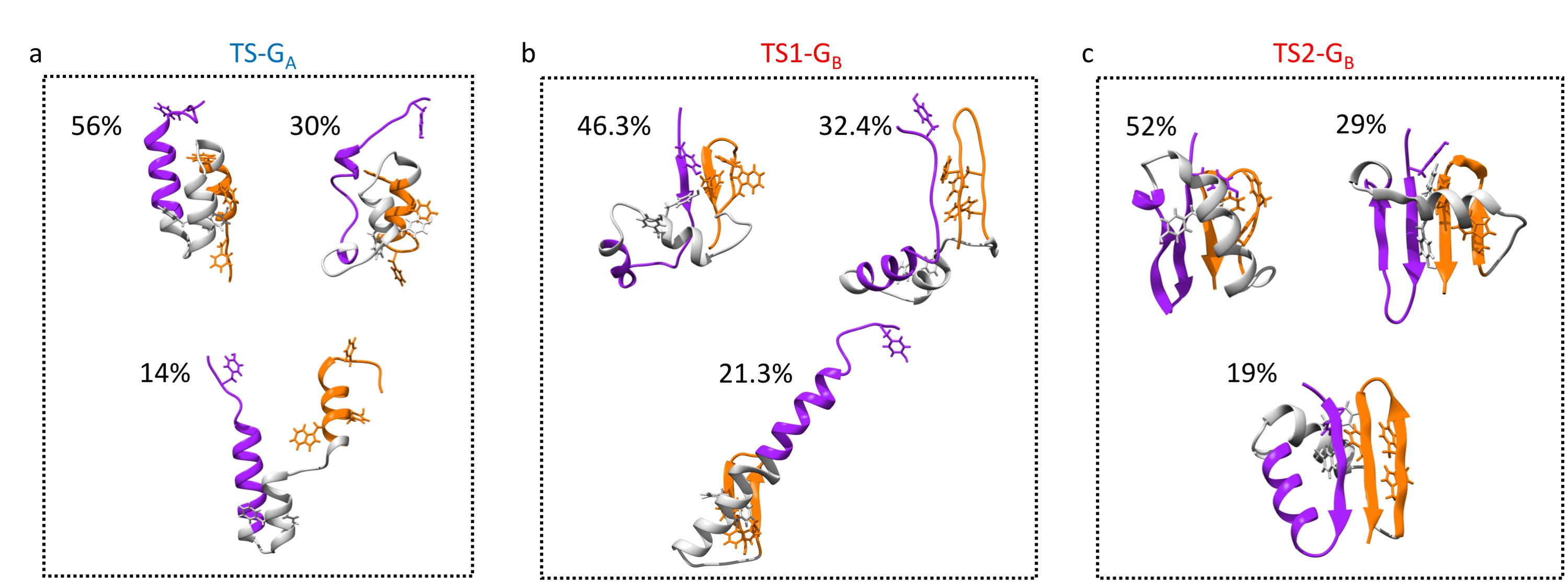

Supplement: S14 Fig — Using the same conformational similarity measure for the k-means clustering of all accessible conformations (Methods of main text), a separate clustering of each of the three putative transition states, (a) TS-GA, (b) TS1-GB, and (c) TS2-GB, was performed for the (a) 453, (b) 834, and (c) 805 sampled conformations, respectively, in the yellow boxes in Fig 4 of main text that defined these states. Each of the putative transition states was partitioned into three clusters (k = 3). The centroid conformations of the clusters are shown here with the percentages of conformations the clusters encompass. (TIFF) [file pcbi.1004960.s014.tiff]

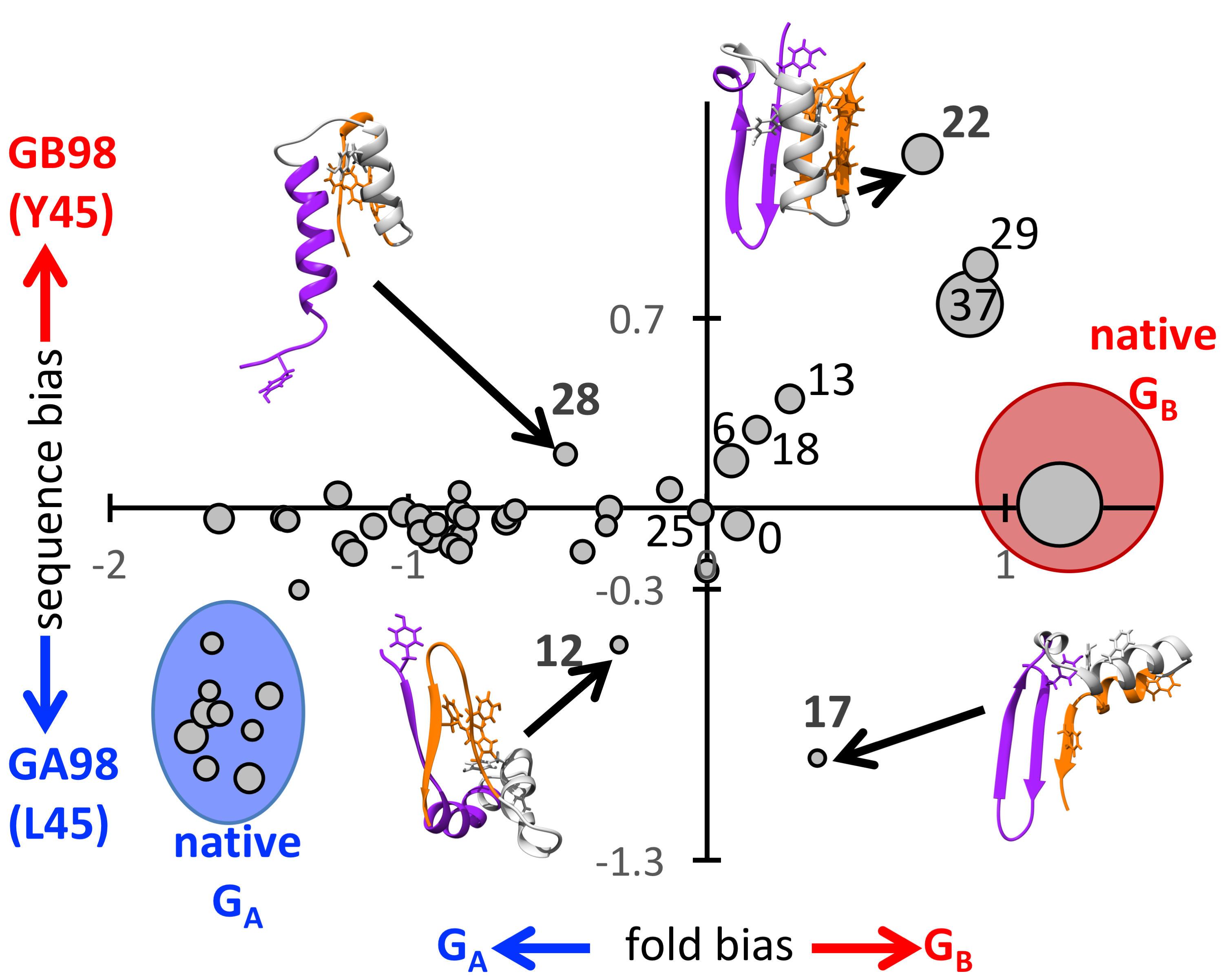

Supplement: S15 Fig — Clusters of conformations presented in Fig 4 of main text were further analyzed. The manner in which cluster size and structural elements are represented is the same as that in Fig 4 of main text. Number labels for select clusters are provided. Here “sequence bias” (vertical axis) is defined as ln[P(GB98)/P(GA98)], where P(GA98) and P(GB98) are the fractions of conformations sampled, respectively, from GA98 and GB98 simulations for the given cluster. ln[P(GB98)/P(GA98)] is the population shift for a cluster after the L45Y mutation; whereas “fold bias” (horizontal axis), defined as ln(QB/QA), is the bias that exists within a given conformational cluster favoring (positive) or disfavoring (negative) GB over GA. The native basins of GA and GB are depicted, respectively, by blue and red ovals. Centroid conformations are shown for the most GB98- and GA98-enriched clusters in the unfolded state (cluster nos. 28 and 12, respectively), as well as the most GB98- and GA98-enriched in the GB intermediate state (cluster nos. 22 and 17, respectively; see text). Note that cluster no. 12 is likely a kinetic trap because its second β-hairpin is in a nonnative orientation, as discussed in conjunction with Fig 4 of main text. (TIFF) [file pcbi.1004960.s015.tiff]

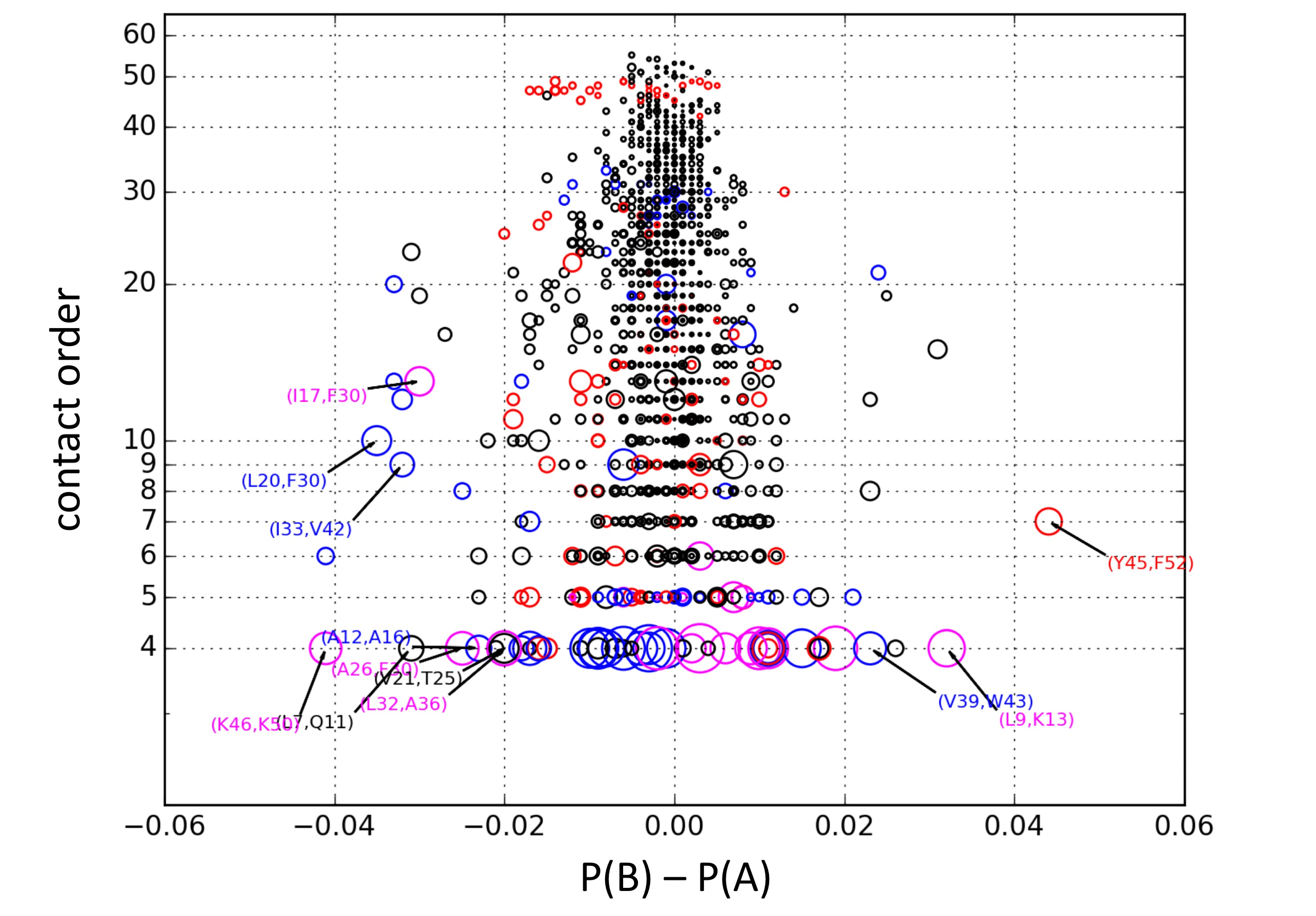

Supplement: S16 Fig — The unfolded state is defined by QA<0.6 and QB<0.3. The criterion for the residue-residue contacts considered here are the same as that for native contacts (Methods of main text). Contact order ≡ |i–j| + 1 for a contact between residues i and j. P(A) and P(B) are the fractions, respectively, of GA98 and GB98 unfolded conformations with a given contact. The diameters of the circles representing the contacts are proportional to the overall fractional frequency [P(A)+P(B)]/2 of the contact. Circle color is used to distinguish contacts that are nonnative (black), native GA (blue), native GB (red), and native GA+GB (magenta). The interacting residue pairs are identified for contacts with the largest frequency shifts. Results in this figure were obtained from an equal number of 1,000 conformations sampled from GA98 and GB98 simulations. (TIFF) [file pcbi.1004960.s016.tiff]

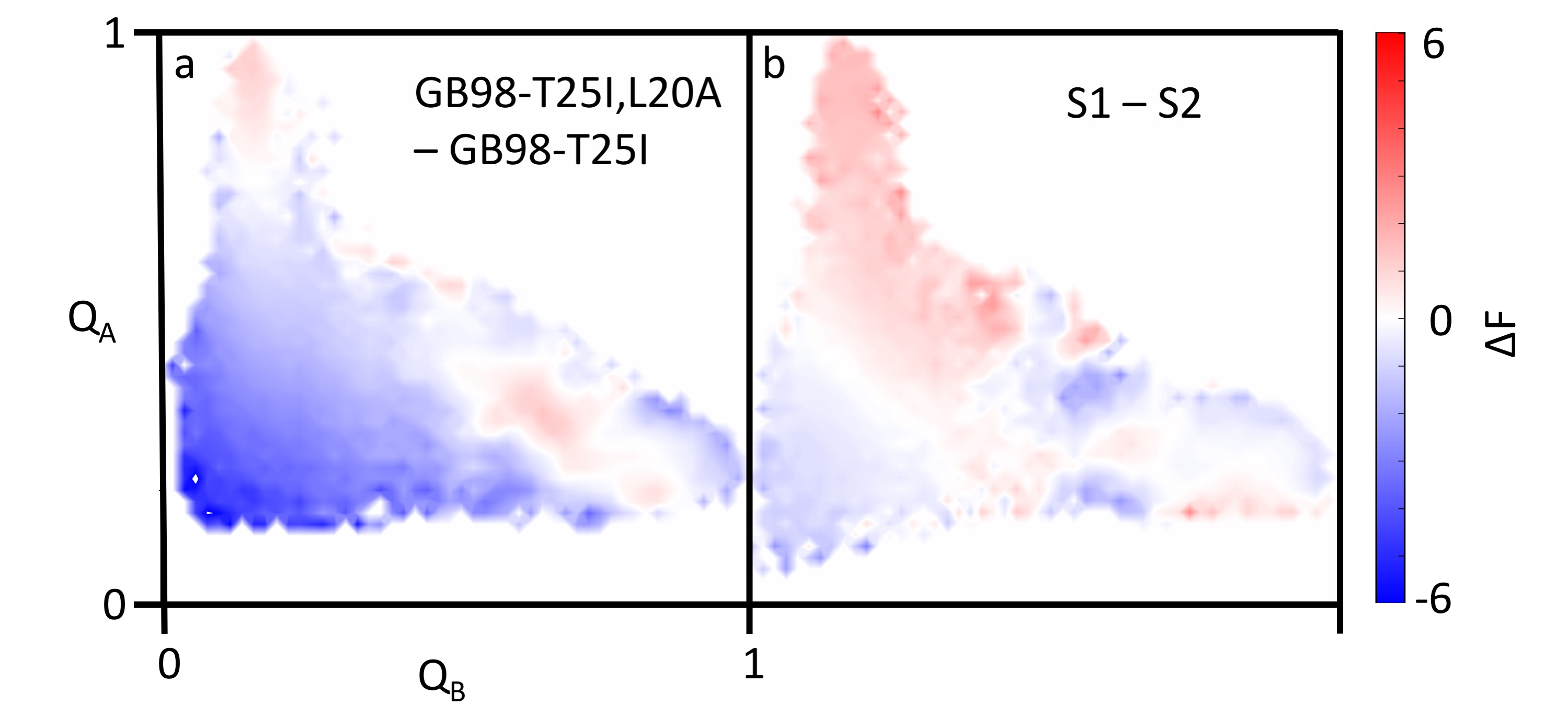

Supplement: S17 Fig — (a) Free energy difference between GB98-T25I,L20A and GB98-T25I (former minus latter) as a function of QA and QB. It is known experimentally that GB98-T25I,L20A adopts the GB fold whereas GB98-T25I adopts the GA fold. (b) The corresponding free energy difference between the predicted switch sequences “S1” (GB fold) and “S2” (GA fold). The free energy landscapes of “S1” and “S2” are given in Fig 7c and 7d of main text. (TIFF) [file pcbi.1004960.s017.tiff]

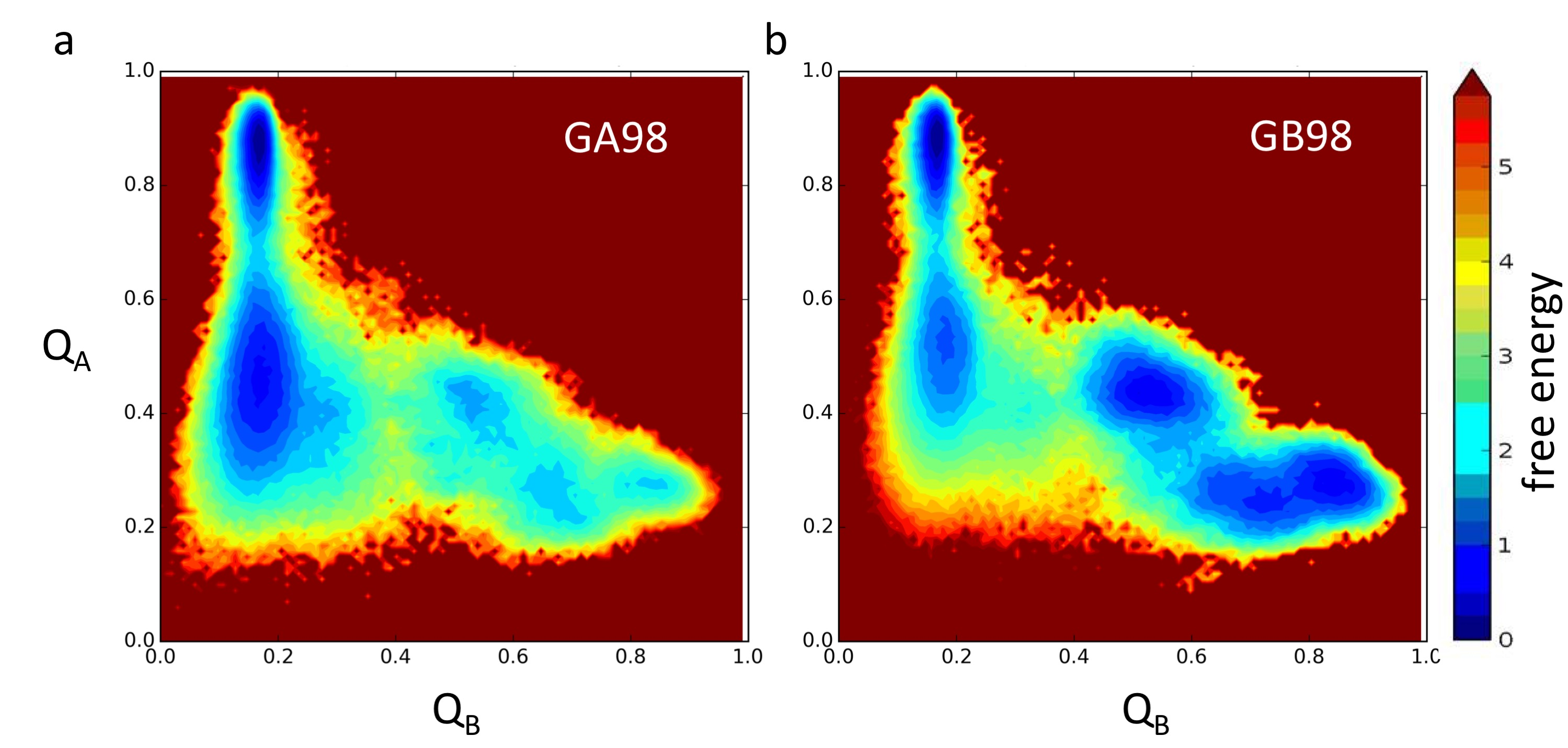

Supplement: S18 Fig — Shown here are the QA/QB free energy landscapes of GA98 (a) and GB98 (b) in the modified hybrid model that incorporates the rudimentary π-π interaction defined in Methods of the main text in the model potential’s transferable component. Relative to the corresponding landscapes in the unmodified hybrid model (Fig 3 of main text), the GB basin of the GA98 landscape here (a) is much more depleted than that of the GB98 landscape (b). (TIFF) [file pcbi.1004960.s018.tiff]
