# Supplementary material for: Theoretical Insights into the Biophysics of Protein Bi-stability and Evolutionary Switches
Source: PLoS Comput Biol. 2016 Jun 2;12(6):e1004960. doi: 10.1371/journal.pcbi.1004960 (PMC4890782; doi:10.1371/journal.pcbi.1004960)
Supplement: S1 Text — (PDF) [file pcbi.1004960.s019.pdf]

Supporting Information

Theoretical insights into the biophysics of protein bi-stability and evolutionary switches

Tobias Sikosek, Heinrich Krobath, Hue Sun Chan (DOI:10.1371/journal.pcbi.1004960)

S1 Text: Modeling details and related computational studies

Calibration of SBM potential

To construct an appropriate dual-basin SBM, we initially attempted two seemingly most straightforward schemes for linearly combining the SBM potentials for G_A and G_B (**S1 Fig** and **S2 Fig**). They entail either equalizing the total SBM energies for the two native structures, i.e., $E_A = E_B = -n_B$, where without loss of generality n_B may be identified with the number of native contacts in G_B, or setting the per-contact energy $\varepsilon_A = \varepsilon_B = -1$ (a constant one may choose to be −1 without loss of generality). In the first case, the per-contact energy of G_A is greater, which in combination with the higher conformational entropic contribution of the disordered termini in G_A leads to the population being largely concentrated in either the G_A basin or the unfolded region (**S3 Fig; a,b**). In the second case, the G_A basin is significantly shallower due to the smaller number of contacts in the G_A contact map (n_A=95 whereas n_B=137), resulting in an overpopulation of the G_B basin with virtually no population in the G_A basin (**S3 Fig; c,d**).

In view of these exploratory results, it is quite clear that a small imbalance in the two SBM strengths of ~4% in favor of G_B would populate both native basins effectively. Structurally, some degree of imbalance is expected from the higher degree of disorder at the termini of G_A compared to G_B. G_B also packs more tightly and has a higher contact order due to its β-rich fold. With these considerations, the native SBM energies (lowest-possible energies) we adopted for most of our simulations were $E_A = 0.96\varepsilon_B n_A$, $E_B = \varepsilon_B n_B$.

As discussed in the main text, we reduced the SBM potential strengths as much as possible to enhance the contributions from the sequence-specific transferable component of the hybrid model potential while maintaining sufficient native bias to enable folding to both G_A and G_B (**S4 Fig**). For instance, at ε_B=−0.25, some degree of folding to G_B was seen but folding to G_A was not observed, with the unfolded state dominant at T_m (**S5 Fig**). After exploring a range of values of ε_B between −2.0 and −0.25, the simulation results at ε_B=−0.37 were seen as most consistent with experiment. Unless stated otherwise, simulation results in the main text were obtained using ε_B=−0.37.

SBM potential strength: robustness of predicted switching behavior

While ε_B=−0.37 was chosen for extensive analysis, the general behavioral trend of our model G_A/G_B system is robust for a reasonably wide range of ε_B values. As apparent in

S4 Fig, the preferences of GA98 and GB98 for the G_A and G_B folds, respectively, are maintained over a range of different ε_B values. A more detailed analysis of this feature of our hybrid model is provided in **S7 Fig**, where free energy surfaces of GA98 and GB98 computed at their respective T_m are shown for different $-\varepsilon_B$ values, in steps of 0.05, between $-\varepsilon_B = 0.2$ and $-\varepsilon_B = 0.5$. The simulation protocol used here is the same as that in the main text. Both the G_A and G_B native basins are stabilized for $0.3 \leq -\varepsilon_B \leq 0.5$, whereas $-\varepsilon_B < 0.3$ is not sufficient to drive G_A folding. Notably, the G_B intermediate is populated significantly for $-\varepsilon_B \leq 0.4$, but is depleted for higher $-\varepsilon_B$, indicating that the G_B intermediate state predicted by our hybrid model is a consequence of the physical transferrable potential, not the Gō-like SBM. **S8 Fig** shows further that the difference in $\Delta F(G_A–G_B) = -\ln(P_A/P_B)$ between GA98 and GB98 (see main text) is approximately constant across different SBM strengths for $0.2 \leq -\varepsilon_B \leq 0.5$. Within this range, G_A is always more favored by GA98 (lower ΔF) than by GB98 (higher ΔF), irrespective of the approximate linear increase of T_m with increasing SBM strength $-\varepsilon_B$ (**S8 Fig, *inset***).

Further illustration of this robustness is provided by Hamiltonian replica exchange simulations (Fukunishi et al., 2002). In addition to the temperature replica exchange discussed in the main text --- where temperatures of different replicas are swapped, we have also conducted constant-temperature Hamiltonian replica exchange simulations, wherein different ε_B values within the range $0.25 \leq -\varepsilon_B \leq 0.50$ were swapped among 32 replicas in accordance with the Metropolis criterion. The results are shown in **S9 Fig** for GA98 and **S10 Fig** for GB98 at a constant temperature essentially equal to their T_ms for $-\varepsilon_B = 0.37$. Because the T_ms for $-\varepsilon_B < 0.37$ are lower (**S8 Fig, *inset***), the free energy landscapes in **S9 Fig** and **S10 Fig** were under unfolding conditions for $-\varepsilon_B < 0.37$. Thus only unfolded conformations are populated and hence bi-stability is not apparent, even though bi-stability is observed for SBM strengths as low as $-\varepsilon_B = 0.30$ when the free energy landscapes were computed at the T_ms of the given ε_B values (**S7 Fig**). Conversely, the free energy landscapes in **S9 Fig** and **S10 Fig** were under increasingly strong folding conditions for $-\varepsilon_B > 0.37$. Under these conditions, G_A is much more favored over G_B for GA98 (**S9 Fig**) than for GB98 (**S10 Fig**). More specifically, GA98 exhibits very little population of the G_B fold for all but the strongest $-\varepsilon_B$ (**S9 Fig**), whereas GB98 populates both the G_B native and intermediate states substantially for all $-\varepsilon_B > 0.37$ (**S10 Fig**). This trend is consistent with that observed in **S7 Fig** and **S8 Fig**.

While the present hybrid model is rather robust for $0.3 \leq -\varepsilon_B \leq 0.5$, it is instructive to note that the model does not reproduce G_A/G_B behaviors at the two extremes of no SBM ($\varepsilon_B = 0$) or no nonlocal transferrable interactions. The $\varepsilon_B = 0$ failure reiterates the fact that to date no transferrable potential by itself can account for G_A/G_B folding and switching. For $\varepsilon_B = 0$ (transferrable potential only), our model samples secondary-structure-rich conformations with a helical bias [$\max(Q_A) > \max(Q_B)$], but the G_A and G_B native states are not visited (**S11 Fig; a**). For the case in which the transferrable component of the potential is artificially limited to nonlocal atomic excluded volume repulsion and local terms that maintain a protein-like backbone geometry (i.e., nonlocal attractive interactions of the transferrable potential such as hydrogen bonding and hydrophobic interactions are not considered), no folding was observed for $-\varepsilon_B = 0.37$. Moreover, the

low Q_A and Q_B values of the sampled conformations suggest a lack of secondary structure in this model setup (**S11 Fig; b, *top***). At $-\varepsilon_B$ = 1, the G_A and G_B folds are substantially populated but so are conformations along the line connecting the two native basins (**S11 Fig b, *bottom***). Hence these free energy landscapes are not bi-stable. It follows from the control simulations in **S11 Fig** that neither the transferrable Lund potential nor the SBM are individually sufficient to produce the G_A/G_B switching results of our hybrid approach.

Transition flux analysis

To characterize the shift in native stabilities in the G_A/G_B system more comprehensively, we partitioned the Q_A/Q_B free energy landscape into either three (**S13 Fig; a**) or eight (**S13 Fig; b**) macroscopic states (lines separating the states are overlaid onto the GB98 landscape as an example in **S13 Fig**). Replica exchange simulation trajectories, where each trajectory is a temporally ordered list of conformational changes undergone by one protein chain simulated under varying temperature and recorded every 1,000 Monte Carlo cycles, were analyzed for the number of transitions between the macroscopic states. Transition frequencies were normalized for comparison between GA/GB sequence variants. In the three-state model, transition frequencies to and from the native states (G_A vs G_B) showed a gradual shift as the sequence varies from GAwt to GBwt (**S13 Fig; c**). For the eight-state model (**S13 Fig; d**), transition frequencies are plotted on a log scale (as a visual aid) and only for the eight sequences GA77 to GB77, where all states were populated to some degree. **S13 Fig (e)** highlights two of these transitions, namely the ones between the native states and their respective neighboring transition state. Here again, the transition frequencies shift along the sequence axis and crossover in between GA98 and GB98. As an internal consistency check of our simulation procedure, these results confirm our free energy calculations indicating a gradual shift in native state preference from G_A to G_B as the sequence varies from GAwt to non-wildtype GA/GB variants and then to GBwt.

Possible molecular basis for L45Y-induced structure switch

Because each cluster in **Fig.4** is a mixture of GA98 and GB98 and the initial pool of conformations before clustering contained an equal number of GA98 and GB98 conformations, any deviation from a 50/50 ratio in a cluster indicates a population shift due to the mutation. **S15 Fig** summarizes these shifts. The horizontal "fold bias" variable separates centroids of clusters of conformations similar to G_A (left) from those similar to G_B (right). The vertical "sequence bias" variable separates the relative per-cluster bias of GA98 vs GB98. Clusters with positive or negative values are enriched, respectively, in GB98 or GA98 conformers. A correlation between fold and sequence bias is seen in **S15 Fig** but there are outliers. All clusters within the G_A native basin (bottom-left, blue) are depleted by L45Y. In contrast, most clusters in the G_B intermediate basin are strongly favored by the same mutation (clusters nos. 6, 18, 13, 22; except cluster 17, see below). The same is true for clusters around TS2-G_B (cluster nos. 29 and 37). One important exception, however, is the largest, most G_B-like native cluster that lies on the horizontal axis (cluster no. 4). This result indicates that according to the transferrable potential here, L45Y favors the G_B-like native intermediates even more than the G_B native fold itself.

The centroid structures of those clusters in the unfolded state or close to TS1-G_B that are most enriched in GB98 conformations (cluster no. 28) or GA98 conformations (cluster no. 12) are shown to the left of the vertical axis in **S15 Fig**. They all feature the second β-hairpin that hosts the mutated Y45, which favorably interacts with F52 on the opposite strand. This observation on unfolded clusters suggests that L45Y likely has a G_B-biasing effect even in the early stages of folding. Interestingly, the kinetically trapped version of the second β-hairpin in the centroid of cluster no. 12 is more favored by GA98 than by GB98 sequences.

We further characterize the L45Y-induced drive toward G_B by quantifying the bias in contact statistics in the entire unfolded state (all conformations with $Q_A < 0.6$ and $Q_B < 0.3$). The aromatic-aromatic Y45-F52 interaction stands out as the one affected most by the L45Y substitution. This contact is more frequent in GB98 compared to GA98 conformations by more than 4%. In contrast, the *i*, *i*+4 helical contact K46-L50 is favored in GA98 compared to GB98 conformations by about 4%. Further details of this analysis are provided in **S16 Fig.**

The centroid conformations of the two most sequence-biased clusters (nos. 17 and 22) in the G_B intermediate basin are shown in **S15 Fig**. Cluster no. 22 is most biased toward GB98. Both β-hairpins are formed in its centroid; and the conformations in this cluster closely resemble those in TS2-G_B (**Fig.4** of main text). In contrast, cluster no. 17 is most biased toward GA98. This cluster is a clear outlier in that its conformational population is biased towards GA98 conformers (negative vertical value) although its contact pattern is biased toward the G_B fold (positive horizontal value). Notably, only the first but not the second β-hairpin is formed in the centroid of this cluster, although stabilizing interactions in the second β-hairpin are important in the formation of the G_B fold. The ambivalence in sequence-structure relationship in this cluster underscores once again the bi-stable nature of the GA98 and GB98 sequences.

Related computational studies

It is instructive to compare and contrast our model predictions with computational results in the literature on G_A and G_B folding. A brief summary of select studies is provided below.

Extensive simulations of coarse-grained chain models have long been performed on GBwt (commonly referred to as protein GB1 or Protein G). The insights they provided into the folding process and their structural characterization of possible intermediate and transition states are of interest. An early Cα Gō-model predicted that GBwt folding is two-state (Karanicolas and Brooks, 2002). In an all-atom Gō-model simulation of GBwt performed around the same time, the major folding pathway was seen to involve an interaction between the helix and the first β-hairpin whereas alternative pathways, including the ones more similar to that suggested by our present GA98/GB98 simulations, have low probabilities (Shimada and Shakhnovich, 2002). However, a subsequent study using a simplified Cα chain model with a physics-based transferable potential found two alternative pathways of GBwt folding: a two-state transition via the interaction of the helix and the second β-hairpin, and a three-state transition where a third β-strand is added and forms a stable intermediate (Brown and Head-Gordon, 2004). Both pathways are similar to what we observed for GA98/GB98 folding (**Fig.4** of main text, and **S14 Fig**).

A subsequent study using a coarse-grained Cα+Cβ (two beads per residue) found that the first major event along the GBwt folding pathway is the formation of the second β-hairpin with strong aromatic interactions and its temperature-dependent association with the helix. The third step is the association of the first and fourth β-strands followed by incorporation of the Y3 residue into the hydrophobic core consisting of aromatic sidechains, resulting in a “fluctuating native-like globule” (Kmiecik and Kolinski, 2008).

Unfolding simulations on the G_A/G_B system have also been conducted. An all-atom explicit-water molecular dynamics study of high-temperature unfolding of GA59 and GB59 – a sequence pair that share 59% amino acid identity – suggested that the second β-hairpin forms early during GB59 folding and associates with the central helix, a process that appears to prevent folding into G_A-like structures (Scott and Daggett, 2007). Nonlocal hydrophobic interactions between aromatics were found to serve an important role in this process. The early unfolding transition state they identified (see Scott and Daggett, 2007, Fig. 8 therein) has lost the hydrogen bonds between the first and second β-strands, similar to some of the conformations in our G_B intermediate state (e.g. cluster no. 22 in **Fig.4** of main text) and TS2-G_B (**Fig.4** of main text and **S14 Fig**). They also found that the second β-hairpin was kinetically more stable against unfolding than the first β-hairpin. A subsequent joint experimental-computational study that also involved all-atom explicit-water molecular dynamics simulations of high-temperature GBwt (GB1) unfolding identified intermediate- and transition-state conformations as well (Morrone et al., 2011a, Fig 6 therein). These authors concluded that GBwt folding is not two-state, but they also stated that the partially folded state identified in their work did not accumulate and therefore was distinct from the intermediate states suggested by previous studies (Morrone et al., 2011a). An explicit-water molecular dynamics simulation of mechanical unfolding of GBwt suggested that the second β-hairpin tends to form early during the folding process as well (Wu et al., 2012).

In conjunction with experiment, all-atom explicit-water molecular dynamics simulations of the disordered unfolded states of the GA88/GB88 pair of sequence variants suggested that GB88 has higher helical content in its unfolded state than GA88 despite the helical folded structure of GA88. Moreover, sidechain hydrogen bonds in the GB88 unfolded ensemble were found to stabilize the second β-hairpin (Morrone et al., 2011b). A recent metadynamics study based on NMR data on protein GB3 (which differs from GBwt by only seven amino acids; see PDB:2OED) also found that “the second β-hairpin represents the initial native element in the folding process” (Granata et al., 2013).

A more recent sequence design study of the G_A/G_B system predicted that the N- and C-terminal regions of the protein are most sensitive to mutations and thus likely to harbor “switch” substitutions like L45Y that leads from GA98 to GB98 (Chen et al., 2016). Their emphasis on the importance of the termini region echoes our transition state analysis, which suggests that the first putative bottleneck for G_B folding, TS1-G_B (**Fig.4**, main text), often entails antiparallel alignment of the termini (see the two most frequent representatives of TS1-G_B in **S14 Fig**).

All the results outlined above are in broad agreement with our findings, based on an analysis of the present GA98/GB98 simulations, that the second β-hairpin forms early in G_B folding. In contrast, all-atom pure SBM (Whitford et al., 2009) simulations found instead that GB folding is seeded by the first β-hairpin. Langevin dynamics simulations based on these models for GA95/GB95 and GA98/GB98 also did not find any stable intermediate along the G_B folding pathway (Kouza and Hansmann, 2012; Sutto and Camilloni, 2012). These differences in results between models with a physical transferable potential (e.g. common molecular dynamics force fields) or a hybrid potential with a transferable component on one hand and pure SBM on the other are likely caused in part by the lack of favorable nonnative interactions in the pure SBM models. For example, the G_B intermediate is less populated for the ε_B = –0.5 cases in **S7 Fig** with a reduced transferrable interaction strength. In contrast, our simulations at ε_B = –0.37 frequently sampled nonnative contacts. Among the unfolded-state contacts most impacted by the L45Y mutation (**S16 Fig**), some nonnative contacts (black circles) are seen to respond to the mutation with a significant shift in favorability.

Possibility of a kinetic trap in G_B folding

Our simulations suggested a kinetic trap in the G_B folding pathway that features an inverted second hairpin (cluster no. 12 in **S15 Fig** and the 32.4%-populated TS1-G_B and 19%-populated TS2-G_B cluster centroid conformations in **S14 Fig**). While the sensitivity of this prediction to variation of model parameters remains to be ascertained, it is worth considering whether such “mirror image” conformations could potentially exist in general. An early high-coordination lattice model study succeeded in intentionally creating the mirror image of protein A by mutations (Olszewski et al., 1996). More mirror-image conformations of helical proteins were reported as well in subsequent investigations (Liwo et al., 1999; Yang et al., 2007). More recently, Onuchic and coworkers (Noel et al., 2012) as well as Scheraga and coworkers (Kachlishvili et al., 2014) have emphasized that mirror-image conformations with secondary structure and tertiary contacts similar to a native structure may sometimes compete with the true native state. In particular, in one of these simulation studies it was demonstrated that metastable mirror-image conformations of the native structure could exist for protein A (i.e. GAwt) and that the two alternative folds are energetically similar (Kachlishvili et al., 2014). Further examples of mirror images of native structures in molecular dynamics simulation were reported for the three-helix bundle α_3W (Shao, 2015). In fact, mirror-image conformations have long been noted in computational structure prediction and NMR refinement, but they have largely been treated as artifacts of a nonoptimal energy function (Pastore et al., 1991; Skolnick et al., 1997; Zhang, 2009). Since the native structure and its mirror image may share many characteristics, experimental probes with low spatial resolution such as circular dichroism may not always be able to resolve them. Ultimately, whether or not true mirror-image conformations of native structures exist, and their prevalence if they do, are questions that need to be settled by further experiments.

Are current experimental techniques sufficiently sensitive to distinguish between the alternative G_B β-strand orientations seen in the present simulations? A recent experimental and simulation study on the five-helix-bundle protein $\lambda_{6\text{-}85}$ describes an off-

pathway trapped state with high native contact fraction (Q=0.7) by using multiple fluorescence markers (Prigozhin et al., 2015). In the future, similar techniques may also be applied to β-rich proteins such as GBwt and its variants. In the folding simulations of GB98 presented here, we only observed mirror images of the intermediate state but not the native state. As it stands, it is an open question whether three-state folding reported for several GB variants (Giri et al., 2012) can be explained in part or in its entirety by a kinetically trapped mirror-image intermediate because it might be difficult for common experimental probes to distinguish such trapped states from a native-like intermediate.

The mirror-image G_B intermediate encountered in our simulations arises from an alternative form of the second β-hairpin. Of relevance here are several computational investigations on the possible occurrence of misfolded states in this hairpin of GBwt (GB1). An early implicit-solvent Monte Carlo simulation study of the 16-residue second β-hairpin region of GBwt found that the Y45-F52 interaction forms early, and that the peptide can form a significant number of nonnative hairpins (Dinner et al., 1999). Further theoretical information is available from subsequent molecular dynamics simulations of the second β-hairpin region in isolation, which has been found in some studies to populate a misfolded hairpin (one strand flipped relative to the other) that has to first unfold before it can proceed toward the correctly folded β-hairpin (Best and Mittal, 2011; Hatch et al., 2014). In another study, a completely misfolded hairpin similar to that in our mirror-image intermediate was found among other misfolded conformations (Pietrucci and Laio, 2009). The prediction of misfolding is not universal, however, as another computational study did not encounter misfolded structures (Juraszek and Bolhuis, 2009). Taken together, these molecular dynamics results on isolated peptides suggest strongly that misfolded second β-hairpins of GBwt do occur, albeit not always in the mirrored configuration found in our trapped intermediates. However, tertiary contacts between the β-hairpin with other parts of GBwt outside the hairpin could facilitate clustering of hydrophobic residues of the hairpin on the “wrong” side in the full G_B structural context.

In any event, potential difficulties in identifying the misfolded hairpin in simulation can be caused by the choice of reaction coordinates. On one hand, backbone RMSD, or the fraction of native hydrogen bonds may group a misfolded hairpin with other unfolded-state structures. On the other hand, some studies may count the misfolded hairpins as native or native-like when using the secondary structure content or the fraction of native contacts formed (Q) as reaction coordinate.

S1 Text References

Best, R.B., Mittal, J., 2011. Microscopic events in β-hairpin folding from alternative unfolded ensembles. Proc Natl Acad Sci USA 108, 11087–11092. doi:10.1073/pnas.1016685108

Brown, S., Head-Gordon, T., 2004. Intermediates and the folding of proteins L and G. Protein Sci 13, 958–70. doi:10.1110/ps.03316004

Chen, S.-H., Meller, J., Elber, R., 2016. Comprehensive analysis of sequences of a protein switch. Protein Sci 25, 135–146. doi:10.1002/pro.2723

Dinner, A.R., Lazaridis, T., Karplus, M., 1999. Understanding beta-hairpin formation. Proc Natl Acad Sci USA 96, 9068–9073. doi:10.1073/pnas.96.16.9068

Fukunishi, H., Watanabe, O., Takada, S., 2002. On the Hamiltonian replica exchange method for efficient sampling of biomolecular systems: Application to protein structure prediction. J Chem Phys 116, 9058–9067. doi:10.1063/1.1472510

Giri, R., Morrone, A., Travaglini-Allocatelli, C., Jemth, P., Brunori, M., Gianni, S., 2012. Folding pathways of proteins with increasing degree of sequence identities but different structure and function. Proc Natl Acad Sci USA 109, 17772–6. doi:10.1073/pnas.1201794109

Granata, D., Camilloni, C., Vendruscolo, M.H., Laio, A., 2013. Characterization of the free-energy landscapes of proteins by NMR-guided metadynamics. Proc Natl Acad Sci USA 110, 6817–6822. doi:10.1073/pnas.1218350110

Hatch, H.W., Stillinger, F.H., Debenedetti, P.G., 2014. Computational study of the stability of the miniprotein Trp-cage, the GB1 β-hairpin, and the AK16 peptide, under negative pressure. J Phys Chem B 118, 7761–7769. doi:10.1021/jp410651u

Irbäck, A., Mitternacht, S., Mohanty, S., 2009. An effective all-atom potential for proteins. BMC Biophys 2, 2. doi:10.1186/1757-5036-2-2

Juraszek, J., Bolhuis, P.G., 2009. Effects of a mutation on the folding mechanism of a β-hairpin. J Phys Chem B 113, 16184–16196. doi:10.1021/jp904468q

Kachlishvili, K., Maisuradze, G.G., Martin, O. a., Liwo, a., Vila, J. a., Scheraga, H. a., 2014. Accounting for a mirror-image conformation as a subtle effect in protein folding. Proc Natl Acad Sci USA. doi:10.1073/pnas.1407837111

Karanicolas, J., Brooks, C.L., 2002. The origins of asymmetry in the folding

transition states of protein L and protein G. Protein Sci 11, 2351–2361. doi:10.1110/ps.0205402

Kmiecik, S., Kolinski, A., 2008. Folding pathway of the B1 domain of protein G explored by multiscale modeling. Biophys J 94, 726–36. doi:10.1529/biophysj.107.116095

Kouza, M., Hansmann, U.H.E., 2012. Folding simulations of the A and B domains of protein G. J Phys Chem B 116, 6645–53. doi:10.1021/jp210497h

Liwo, A., Lee, J., Ripoll, D.R., Pillardy, J., Scheraga, H.A., 1999. Protein structure prediction by global optimization of a potential energy function. Proc Natl Acad Sci USA 96, 5482–5485. doi:10.1073/pnas.96.10.5482

Morrone, A., Giri, R., Toofanny, R.D., Travaglini-Allocatelli, C., Brunori, M., Daggett, V., Gianni, S., 2011a. GB1 is not a two-state folder: Identification and characterization of an on-pathway intermediate. Biophys J 101, 2053–2060. doi:10.1016/j.bpj.2011.09.013

Morrone, A., McCully, M.E., Bryan, P.N., Brunori, M., Daggett, V., Gianni, S., Travaglini-Allocatelli, C., 2011b. The denatured state dictates the topology of two proteins with almost identical sequence but different native structure and function. J Biol Chem 286, 3863–72. doi:10.1074/jbc.M110.155911

Noel, J.K., Schug, A., Verma, A., Wenzel, W., Garcia, A.E., Onuchic, J.N., 2012. Mirror images as naturally competing conformations in protein folding. J Phys Chem B 116, 6880–6888. doi:10.1021/jp212623d

Olszewski, K.A., Kolinski, A., Skolnick, J., 1996. Folding simulations and computer redesign of protein A three-helix bundle motifs. Proteins Struct Funct Genet 25, 286–299. doi:10.1002/(SICI)1097-0134(199607)25:3<286::AID-PROT2>3.0.CO;2-E

Pastore, A., Atkinson, R.A., Saudek, V., Williams, R.J., 1991. Topological mirror images in protein structure computation: An underestimated problem. Proteins 10, 22–32. doi:10.1002/prot.340100104

Pietrucci, F., Laio, A., 2009. A collective variable for the efficient exploration of protein beta-sheet structures: Application to SH3 and GB1. J Chem Theory Comput 5, 2197–2201. doi:10.1021/ct900202f

Prigozhin, M.B., Chao, S.-H., Sukenik, S., Pogorelov, T. V, Gruebele, M., 2015. Mapping fast protein folding with multiple-site fluorescent probes. Proc Natl Acad Sci USA 112, 7966–7971. doi:10.1073/pnas.1422683112

Scott, K.A., Daggett, V., 2007. Folding mechanisms of proteins with high sequence identity but different folds. Biochemistry 46, 1545–1556. doi:10.1021/bi061904l

Shao, Q., 2015. Important roles of hydrophobic interactions in folding and charge interactions in misfolding of α-helix bundle protein. RSC Adv 5, 4191–4199. doi:10.1039/C4RA14265A

Shimada, J., Shakhnovich, E.I., 2002. The ensemble folding kinetics of protein G from an all-atom Monte Carlo simulation. Proc Natl Acad Sci USA 99, 11175–11180. doi:10.1073/pnas.162268099

Skolnick, J., Kolinski, A., Ortiz, A.R., 1997. MONSSTER: a method for folding globular proteins with a small number of distance restraints. J Mol Biol 265, 217–241. doi:10.1006/jmbi.1996.0720

Sutto, L., Camilloni, C., 2012. From A to B: a ride in the free energy surfaces of protein G domains suggests how new folds arise. J Chem Phys 136, 185101. doi:10.1063/1.4712029

Whitford, P.C., Noel, J.K., Gosavi, S., Schug, A., Sanbonmatsu, K.Y., Onuchic, J.N., 2009. An all-atom structure-based potential for proteins: bridging minimal models with all-atom empirical forcefields. Proteins 75, 430–41. doi:10.1002/prot.22253

Wu, X., Yang, G., Zhou, L., 2012. Identifying the intermediates during the folding/unfolding of protein GB1 with MD simulations. Theor Chem Acc 131, 1–5. doi:10.1007/s00214-012-1229-4

Yang, J.S., Chen, W.W., Skolnick, J., Shakhnovich, E.I., 2007. All-atom ab initio folding of a diverse set of proteins. Structure 15, 53–63. doi:10.1016/j.str.2006.11.010

Zhang, Y., 2009. I-TASSER: Fully automated protein structure prediction in CASP8. Proteins Struct Funct Bioinform 77, 100–113. doi:10.1002/prot.22588
